# Lipid Profiling and Transcriptomic Analysis Reveals a Functional Interplay between Estradiol and Growth Hormone in Liver

**DOI:** 10.1371/journal.pone.0096305

**Published:** 2014-05-09

**Authors:** Leandro Fernández-Pérez, Ruymán Santana-Farré, Mercedes de Mirecki-Garrido, Irma García, Borja Guerra, Carlos Mateo-Díaz, Diego Iglesias-Gato, Juan Carlos Díaz-Chico, Amilcar Flores-Morales, Mario Díaz

**Affiliations:** 1 Department of Clinical Sciences, University of Las Palmas de Gran Canaria - Biomedical and Health Research Institute (IUIBS), Molecular and Translational Endocrinology Group, Las Palmas de Gran Canaria, Spain; 2 Cancer Research Institute of The Canary Islands (ICIC), Las Palmas de Gran Canaria, Canary Islands, Spain; 3 Department of Animal Biology, University of La Laguna, Laboratory of Membrane Physiology and Biophysics, La Laguna, Spain; 4 Molecular Endocrinology group, University of Copenhagen - Novo Nordisk Center for Protein Research, Copenhagen, Denmark; Massachusetts General Hospital, United States of America

## Abstract

17β-estradiol (E2) may interfere with endocrine, metabolic, and gender-differentiated functions in liver in both females and males. Indirect mechanisms play a crucial role because of the E2 influence on the pituitary GH secretion and the GHR-JAK2-STAT5 signaling pathway in the target tissues. E2, through its interaction with the estrogen receptor, exerts direct effects on liver. Hypothyroidism also affects endocrine and metabolic functions of the liver, rendering a metabolic phenotype with features that mimic deficiencies in E2 or GH. In this work, we combined the lipid and transcriptomic analysis to obtain comprehensive information on the molecular mechanisms of E2 effects, alone and in combination with GH, to regulate liver functions in males. We used the adult hypothyroid-orchidectomized rat model to minimize the influence of internal hormones on E2 treatment and to explore its role in male-differentiated functions. E2 influenced genes involved in metabolism of lipids and endo-xenobiotics, and the GH-regulated endocrine, metabolic, immune, and male-specific responses. E2 induced a female-pattern of gene expression and inhibited GH-regulated STAT5b targeted genes. E2 did not prevent the inhibitory effects of GH on urea and amino acid metabolism-related genes. The combination of E2 and GH decreased transcriptional immune responses. E2 decreased the hepatic content of saturated fatty acids and induced a transcriptional program that seems to be mediated by the activation of PPARα. In contrast, GH inhibited fatty acid oxidation. Both E2 and GH replacements reduced hepatic CHO levels and increased the formation of cholesterol esters and triacylglycerols. Notably, the hepatic lipid profiles were endowed with singular fingerprints that may be used to segregate the effects of different hormonal replacements. In summary, we provide *in vivo* evidence that E2 has a significant impact on lipid content and transcriptome in male liver and that E2 exerts a marked influence on GH physiology, with implications in human therapy.

## Introduction

17β-estradiol (E2), a major natural estrogen in mammals, has physiological actions not limited to reproductive organs in males [1], [2], [3], [4], [5], [6]. Studies in patients with natural mutations in the human estrogen receptor alpha (ERα) [7], [8] and aromatase [9], [10] genes, and in the ERα (ERKO) and aromatase (ArKO) null mice models have shown that E2 can play a critical physiological role in males [1]. In particular, an insufficient E2 signaling in the ERKO and ArKO null mice models results in a metabolic syndrome-like phenotype with fatty liver due to a disruption in β-oxidation and increased lipogenesis, a phenotype that is reversed by physiological doses of E2. Moreover, both of these models exhibit a sexually dimorphic fatty liver that, notably, is male specific [1].

The effects of E2 in the liver can be explained through the direct actions of ER [2], [11], [12], [13] or, indirectly, by modulating growth hormone (GH) physiology [14], [15]. E2 can influence pituitary GH secretion but also GH direct actions in the liver. In particular, E2 induces the expression of Suppressor of Cytokine Signaling (SOCS)-2, which is a negative regulator of the GHR-JAK2-STAT5 signaling pathway [16]. Recently, we have identified SOCS2 as an important regulator of hepatic homeostasis (i.e., lipid and glucose metabolism and inflammation) under conditions of high-fat dietary stress [17]. The ability of GHR-JAK2-STAT5 signaling pathway to regulate hepatic lipid metabolism has also been highlighted in recent mouse genetic studies showing that hepatic inactivation of the GHR [18], its associated kinase, JAK2 [19] or its downstream signaling intermediary, STAT5b [20], leads to fatty liver. The metabolic influence of GH deficiency has also been well documented in humans by the development of a metabolic syndrome (i.e, increased visceral obesity, reduced lean body mass and fatty liver), a phenotype that is ameliorated by GH replacement therapy [21]. Notably, oral administration of pharmacological doses of E2 in humans inhibits GH-regulated endocrine (e.g., IGF-I) and metabolic (e.g., lipid oxidation, protein synthesis) effects [22], [23] but these effects are attenuated when E2 is administered transdermally, suggesting that liver is the major target of regulatory cross-talk between estrogens and GH. However, the molecular characterization of the hepatic changes induced by long-term E2 treatment, when it is administered subcutaneously, and how they influence the liver response to male pattern of GH administration are not well understood.

Animal studies of hepatic effects of E2 or its interplay with GH actions have been focused on females [24], [25]. Nonetheless, it is unclear if males exhibit equivalent responses, and there are reasons why such equivalence should not be presumed. In particular, gender dimorphism in GH secretion patterns develops soon after birth and the pituitary GH release maintains a sexually dimorphic liver function in adulthood [15], which may influence the nature of E2 effects in the livers of males and females. Several GH deficient models can be used to study the interplay between E2 and GH in males. Notably, the hypothyroid-orchidectomized (TXOX) rat model reaches very low or undetectable blood levels of GH and E2, which can be readily restored by hormone replacement treatment (HRT) [25], [26], [27], and shows systemic and hepatic metabolic disturbances with features that mimic deficiencies in E2 [2] and GH [28] (e.g., hypercholesterolemia, adiposity, fatty liver). In this study, we hypothesized that functional interplay between E2 and GH influences liver physiology in male. To test this hypothesis, we investigated the mechanisms of E2 and GH to regulate liver function at the molecular level. We studied gene expression profiles in liver tissue and correlated them with the changes in hepatic lipid content in TXOX rats before and after E2 and/or GH replacement. The results show that the interactions with GH contribute to multiple effects of E2 in male rat liver. Indeed, we found that E2 significantly influenced the GH-regulated endocrine, metabolic, immune, and gender specific responses in the liver. E2- and GH-induced changes in hepatic gene expression profiles were associated with changes in hepatic lipid composition. Finally, hepatic lipid profiles from E2- and/or GH-treated TXOX rats substantially differ from those observed in intact animals, indicating that the normal functions of thyroid glands and testes are an absolute requirement for physiological hepatic lipid homeostasis.

## Material and Methods

### Materials

Recombinant human GH was kindly donated by Pfizer laboratories (Spain). E2 benzoate, Tri-Reagent and, unless otherwise indicated, the rest of the products cited in this work were purchased from the Sigma Chemical Co. (St. Louis, MO).

### Animal treatment

This study was carried out in strict accordance with the recommendations in the Guide for the Care and Use of Laboratory Animals of the University of Las Palmas de Gran Canaria and conducted in accordance with European and Spanish laws and regulations. The protocol was approved by the Committee on the Ethics of Animal Experiments of the University of Las Palmas de G.C. (permit number: 2006-07824). All efforts were made to minimize suffering. Adult (2–3 months old) male Sprague-Dawley rats (n = 6 per group) were used throughout these experiments. Animals were kept under a constant dark/light cycle, and in a controlled temperature (21–23°C) environment, and had free access to autoclaved standard chow (A04 SAFE Panlab, Barcelona, Spain) and tap water throughout the experiment. The generation of hypothyroid animals was performed as previously described [27], [29]. The goitrogenic drug methimazole (MMI; 0.05%) was added to the drinking water for 5 weeks starting on postnatal day (PND) 59 until sacrifice on PND94. Calcium chloride (1%) was included with MMI in the water to ensure adequate dietary calcium intake because hypothyroidism decreases food intake by up to 40% [25]. The MMI-containing water was changed twice per week. Two weeks after starting MMI administration, rats were orchidectomized (OX) or sham–operated to make TXOX or testis-intact hypothyroid (TX) groups, respectively. Six rats were not treated with MMI and were subjected to sham-surgery to provide euthyroid testis-intact controls (INTACT). Four days after OX, we began HRT with E2 benzoate (50 µg/kg; sc; 5 days per week, from Monday to Friday) (TXOXE2) or vehicle (0.2 ml corn oil; sc; 5 days per week, from Monday to Friday) (TXOX) to TXOX rats for 20 days [30], [31] before hormonal replacement for 7 days with either E2 plus GH (TXOXE2GH) or vehicle plus GH (TXOXGH). GH (0.3 mg/kg/day) was administered as two daily sc injections at 12-h intervals (08:00h and 20:00h) to mimic the male-specific GH secretion [32], [33]. TX and TXOX control animals received equivalent amounts of the vehicle alone. Hypothyroidism status was corroborated by monitoring the body weight gain at 7 day intervals and the serum levels of T4 and T3. Twenty-four hours (in the case of E2) or twelve hours (in the case of GH) after the last injection, the animals were killed by exsanguinations. On PND94, blood samples were collected and serum stored at −80°C until analysis. Portions of the liver were snap frozen in liquid nitrogen and stored at −80°C until processed for mRNA analysis.

### Serum analysis

The blood was analyzed for T3, T4, glucose, cholesterol (CHO), triacylglycerols (TG), leptin, IGF-I, E2, and testosterone (T). Serum free T3 and T4 concentrations were measured in duplicate by enzyme immunoassay (Access Systems, Beckman Coulter, Inc), with a detection limit of 0.60 ng/dl and 88 ng/dl, respectively. Serum levels of glucose, CHO, and TG were quantified by using an Olympus AU2700 chemistry analyzer (Beckman Coulter Inc.). The immunoassay method was also used to determine serum levels of E2 and T by using the UniCel DxI 800 immunoassay system (Beckman Coulter Inc). Serum levels of leptin and IGF-I were determined by using rat immunoassays (Quantikine, R&D systems) according to manufacturer recommendations. The IGF-I and leptin assays included quality controls provided by the manufacturer, and the standard curves of the assays were performed in accordance with the manufacturer's provided samples. All the samples were assayed together and each sample was assayed in duplicate.

### Hepatic lipid analysis

Liver lipids were analyzed following the procedures detailed in Fabelo et al. [34]. Briefly, total lipids were extracted with chloroform/methanol (2∶1 v/v) containing 0.01% butylated hydroxytoluene (BHT) as an antioxidant. Lipid classes were separated by one-dimensional double-development high-performance thin-layer chromatography (HPTLC) using methyl acetate/isopropanol/chloroform/methanol/0.25% (w/v) KCl (5∶5∶5∶2∶1.8 by vol.) as the developing solvent system for the polar lipid classes and hexane/diethyl ether/acetic acid (22.5∶2.5∶0.25 by vol.) for the neutral lipid classes. Lipid classes were quantified by densitometry using a Shimadzu CS-9001PC spot scanner. Total and neutral lipid fractions were subjected to acid-catalyzed transmethylation for 16 h at 50°C using 1 ml of toluene and 2 ml of 1% sulfuric acid (v/v) in methanol. The resultant fatty acid methyl esters (FAME) were purified by TLC, and visualized by spraying with 1% iodine in chloroform [35]. FAME were separated, and quantified by using a Thermo gas chromatograph equipped with a flame ionization detector (250°C) and a fused silica capillary column Supelcowax TM 10 (30 m×0.32 mm I.D.). Individual FAMEs were identified by referring to authentic standards. Equal amounts of total lipids were used in all analyses. Throughout the manuscript, lipid nomenclature adhered LIPID MAPS classification system (http://www.lipidmaps.org/data/structure/index.html).

### RNA isolation, cDNA microarray, probe preparation, and hybridization

Total RNA was isolated by homogenization of frozen rat tissues using a polytrone PT-2000 (Kinematica AG) and TriReagent (Sigma, St. Louis, MO) according to the protocol supplied by the manufacturer. All samples were treated with RNAse-free DNase (Promega, Madison, WI). RNA yields were measured by UV absorbance and the quality of total RNA was analyzed by using a 2100 Bioanalyzer (Agilent Technologies, Palo Alto, CA). A microarray containing 27000 rat 70-mer oligo probe sets produced at the KTH Microarray Center (www.biotech.kth.se) was used to evaluate the effects of hypothyroidism and hormonal replacement in TXOX animals on liver gene expression. Five µg of high-quality total RNA from the liver were reversed-transcribed, labeled, and hybridized following the manufacturer's protocol (Pronto Plus System, Promega). After 16 h of hybridization, the slides were washed and scanned using the GenePix Microarray Scanner (Axon Instruments, CA). Four independent hybridizations were performed comparing individual animals from the different experimental groups for a total of 4 analyses.

### Microarray data processing and analysis

Image analysis was performed using the GenePix Pro 6.0 software (Axon Instruments, Union City, CA) as previously described [36]. The LOWESS (Locally Weighted Scatter Plot Smoother) method was used to normalize the raw intensity data [37]. If the measured probe sets were not present in at least 3 of the 4 chips, they were assumed to contain no information and therefore were eliminated to reduce data complexity. Differentially expressed genes were identified by using the SAM (Significance Analysis for Microarrays) statistical technique [38]. A *q* value was assigned for each of the detectable genes in the array. This value is similar to a *P*-value, measuring the lowest false discovery rate (FDR) at which differential expression of a gene is considered significant. A minimal FDR of 0.05 was assigned for each gene. In this work, a completed list of regulated genes is available as supplementary (S) files. An additional selection requirement was added to FDR based on absolute changes in the gene expression ratios. A value of 1.5 (50%) (log_2_ ratio ≥|0.58|) was chosen to describe ratios as up- or down-regulated. The microarray data discussed in this publication have been deposited in NCBÍs Gene Expression Omnibus [39] and are accessible through GEO Series accession number GSE50014 (www.ncbi.nlm.nih.gov/geo). Functional and system biological network analyses were performed on the basis of the Gene Ontology (GO) enrichment of differentially expressed genes in liver using DAVID [40], and the results were depicted using Cytoscape [41]. For the graphical representation, the significance cut-off was set to a *p* value <0.05 and a corrected *q* value (Benjamini) <0.1. GO graphs interpretation: node (inner circle) size corresponds to the number of genes up-regulated by GH or E2; node border (outer circle) size corresponds to the number of genes down-regulated by GH or E2; color of the node and border corresponds to the significance of the gene set for up or down regulated genes, respectively (dark red = significantly enriched, light red = enriched no significantly; grey = absent); edge size corresponds to the number of genes that overlap between the two connected gene sets. Green edges correspond to shared up-regulated genes and blue edges correspond to shared down-regulated genes.

### Analysis of gene expression by real-time quantitative-PCR (qPCR)

The mRNA expression levels of genes were measured using qPCR. Briefly, 2 µg of total RNA was treated with RNase-free DNase I (Promega) to remove genomic DNA and reverse transcribed using iScript (Bio-Rad) according to the manufacturer's instructions. Two µl of cDNA served as a template in a 20 µl qPCR reaction mix containing the primers and SYBR Green PCR Master Mix (Diagenode, Belgium). Quantification of gene expression was performed according to the manufacturer's protocol using ABI PRISM 7000 SD RT-PCR. A relative standard curve was constructed with serial dilutions (1∶1, 1∶10, 1∶100) using a pool of the cDNA generated from all animals used in the study. The amplification program consisted of 1 cycle of 95°C for 10 min, followed by 45 cycles of 95°C for 15 s, annealing for 10 s, and 72°C for 30 s. The fluorescent intensity was measured at a specific acquisition temperature for each gene. A dissociation protocol was performed to assess the specificity of the primers and the uniformity of the PCR generated products. The amplified PCR products were subjected to agarose electrophoresis to confirm their predicted size. Data were extracted and amplification plots generated with ABI SDS software. All amplifications were performed in duplicate, and C_t_ scores were averaged for subsequent calculations of relative expression values. The level of individual mRNA measured by qPCR was normalized to the level of the housekeeping gene cyclophilin by using the Pfaffl method [42]. Exon-specific primers (Table S1) were designed by the Primer 3 program [43].

### Statistical analysis

The significance of differences between groups was tested by one-way ANOVA, followed by post hoc comparisons of group means according to the GraphPad Prism 5 program (GraphPad Software, San Diego, CA). Statistical significance was reported if *P*<0.05 was achieved. For graphing purposes in the qPCR analysis, the relative expression levels were scaled so that the expression level of the INTACT group equaled one. Lipids classes and main fatty acids were additionally submitted to factor analysis by means of Principal Component Analysis (PCA) [44]. Variable extraction was carried out based on the proportion of total variance explained by two principal components. Factor scores for principal component 1 in multivariate analyses of lipid classes, and fatty acids from total and neutral lipid are depicted. Factor scores were further analyzed by one-way and to assess statistical differences between treatments and by two-way ANOVA to evaluate the combined effects of hormonal treatments and their interactions, as we have previously reported [45].

## Results

### Estradiol inhibits the effects of GH on somatotropic-liver axis and induces negative regulators of GH-STAT5 signaling and a female-pattern of gene expression in adult hypothyroid-orchidectomized rat liver

**Figure 1:**
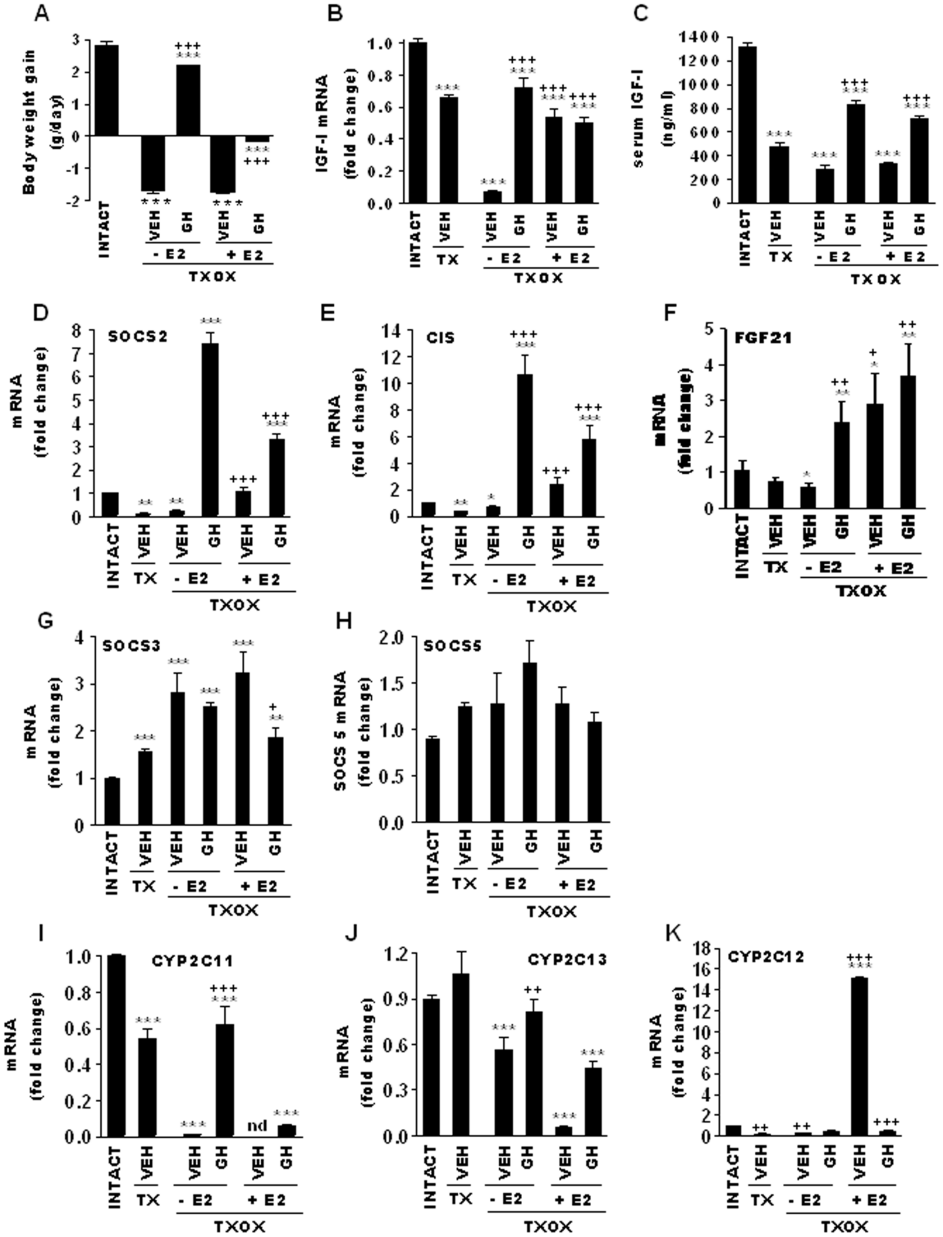
E2 inhibits the effects of GH on somatotropic-liver axis and induces negative regulators of GH-STAT5 signaling and a female-pattern of gene expression in hypothyroid-orchidectomized rat liver. Euthyroid testis-intact controls (INTACT) and hypothyroid-orchidectomized (TXOX) rats were described in Material and Methods. E2 or vehicle (VEH) administration to TXOX rats was performed for 20 days. Then, GH replacement during 7 days rats was carried out in TXOX in the absence (-E2) or in the presence (+E2) of E2. On PND90, body weight gain (A), hepatic IGF-I mRNA (B), circulating IGF-I (C), SOCS2 (D), CIS (E), FGF21 (F), SOCS3 (G), SOCS5 (H), CYP2C11 (I), CYP2C13 (J), and CYP2C12 (K) were measured by qPCR. Results are expressed as mean ± S.D. (n = 6). ***, P<0.001 for comparison with vehicle treated INTACT group; +, P<0.05, ++, P<0.01, +++, P<0.001 for comparison with vehicle treated TXOX group.

Upon sacrifice on PND94, biochemical hypothyroidism was shown, and significantly (*P* = 0.001) lower or undetectable serum levels of T3 (ng/dl) [36.17±5.43 (INTACT); 7.92±4.84 (TXOX); 1.48±2.14 (TXOXE2); 0 (TXOXGH); 0.14±0.33 (TXOXE2GH)] and T4 (ng/dl) [(1.90±0.17 (INTACT) *vs*. 0)] were found in all TXOX groups in comparison with the age-matched euthyroid control group (INTACT). Serum E2 levels (pg/ml) [6.33±5.57 (INTACT); 438.80±122.12 (TXOXE2); 363.10±107.95 (TXOXE2GH)] were increased (*P* = 0.001 *vs* INTACT) in E2-treated TXOX rats up to 5-10 times those observed in rats during pregnancy [46] or the proestrus phase of the reproductive cycle [12]. A first approach to assess the effects of E2 on TXOX rat liver was made through analysis of its influence on the somatotropic-liver axis. TXOX caused impaired growth, which was evident as a reduction in daily body weight gain (Fig. 1A). At the last measurement, on PND94, a significant difference (*P*<0.001) in body weight remained between INTACT (447±33 g) and TXOX (338±28 g) groups. Accordingly, TXOX showed reduced levels of hepatic IGF-I mRNA (Fig. 1B) and circulating IGF-I (Fig. 1C). Treatment of TXOX rats with GH, partially or totally, restored body weight gain (Fig.1A), liver IGF-I mRNA (Fig.1B), and circulating IGF-I (Fig.1C), whereas these effects were prevented in the presence of E2. Notably, E2 administration to TXOX rats increased hepatic IGF-I mRNA to the level of non-orchidectomized-hypothyroid (TX) rats (Fig.1B). Next, we carried out mRNA quantitative analysis of SOCS2, CIS, and FGF21, which are negative regulators of GHR-STAT5 signaling [47], [48]. E2 treatment of TXOX rats induced the mRNA expression of SOCS2 (Fig. 1D) and CIS (Fig.1E), but only to a fraction of the levels obtained after GH treatment. Indeed, when E2 was used in combination with GH, a 2-3-fold reduced mRNA expression levels of SOCS2 (Fig. 1D) and CIS (Fig.1E) were observed compared with GH treatment alone, again demonstrating the inhibitory actions of E2 on GH hepatic actions. In contrast, the positive effect of GH on FGF21 mRNA was not affected in the presence of E2 (Fig.1F). The level of SOCS3 mRNA was induced by hypothyroidism itself (Fig.1G), whereas neither hypothyroidism nor hormonal replacement altered SOCS5 (Fig.1H). Finally, we measured GH-regulated gene markers of liver sexual dimorphism [15]. Figure 1I shows that the mRNA expression level of CYP2C11, a male-specific gene, was completely abolished in TXOX liver, whereas it was recovered by intermittent GH replacement. In contrast, E2 prevented the GH-induced mRNA expression levels of CYP2C11 (Fig.1I) and CYP2C13 (Fig.1J). Unlike intermittent GH, E2 induced the female-specific CYP2C12 gene in TXOX liver (Fig.1K). Our microarray analysis (see below) also showed that male predominant genes (e.g., CYP2C11, CYP2C13, CYP2E1, alpha-2u-globulin) were down-regulated by E2, whereas female predominant genes (e.g., CYP2C12, CYP2A1, CYP2C7) were induced [15]. Overall, these findings demonstrate that E2 influences the transcription of GHR-STAT5 targeted genes and induces a female-pattern of gene expression in TXOX rat liver.

### Influence of estradiol on serum and hepatic lipids in hypothyroid-orchidectomized rats

The effects of E2 and GH on hepatic lipid composition were explored in TXOX rats by carrying out a quantitative analysis of lipid classes (Table 1) and fatty acids from total (Table 2) and neutral (Table 3) lipids. The development of a hypothyroid state in male rats was accompanied by altered circulating lipids: a 2-fold increase of total CHO and a 3-fold decrease of TG (Table 1). However, these changes were not prevented by E2 or GH replacement. Hypothyroidism increased the hepatic levels of CHO and decreased those of TG and diacylglycerols (DG) in comparison with the INTACT group (Table 1). However, the levels of free fatty acids (FFA) were not significantly affected by hypothyroidism, though the average values were lower than in the INTACT animals. Among fatty acids from neutral lipids (Table 3), TXOX rats contained increased levels of total saturated fatty acids (SFA) (due to the significant increase in18:0 content). Among monounsaturated fatty acids (MUFAs), 18:1n-9 (oleic acid), 16:1n-7 (palmitoleic acid), and 18:1n-7 (vaccenic acid) were all significantly reduced in neutral lipids (where these MUFAs are abundant) compared with INTACT animals. Interestingly, these changes paralleled the increase in 18:0. These findings suggest an alteration of Δ9 desaturase in TXOX animals. The most representative 18 carbon polyunsaturated fatty acids (PUFAs), 18:2n-6 (linoleic acid) and 18:3n-3 (linolenic acid) from total (Table 2) or neutral (Table 3) lipids, were unaffected in TXOX. Noticeably, TXOX rats exhibited increased levels of 20:4n-6 [arachidonic acid (AA)] and reduced levels of its elongated precursor 22:4n-6. This later effect on AA was a direct consequence of hypothyroidism and orquidectomy and could be restored by the hormonal therapies used here, especially when used in combination. Another physiologically relevant very long chain polyunsaturated fatty acid (VLCPUFA), namely 22:6n-3 (DHA), exhibited a significant reduction in total lipids from TXOX rats (Table 2). As expected, levels of essential fatty acids 20:4n-6 and 22:6n-3 in INTACT and TXOX animals were three-to-four times higher in total lipids than in neutral lipids, confirming their preferential location within membrane phospholipids. Moreover, the results from total lipids indicate that hypothyroidism and orquidectomy strongly affect the acylation of DHA-containing phospholipids in liver cells. These findings are physiologically relevant since depletion of membrane 22:6n-3 is known to severely impact physicochemical properties of cell membrane [45], [49].

**Table 1:** Effects of hormonal replacement on serum and hepatic lipid classes.

Lipid classes	INTACT		TXOX		TXOXE2		TXOXGH		TXOXE2GH	
	mean ± SEM		mean ± SEM		mean ± SEM		mean ± SEM		mean ± SEM	
Serum										
Glucose (g/dl)	141,2±4.7	a	126,8±6,2	ab	112,0±3,9	bc	107,7±7,1	bc	95,0±3,6	d
CHO (mg/dl)	67,2±3	a	126,5±10,1	b	141,2±12,9	b	129,3±12,9	b	143,5±6,4	b
TG (mg/dl)	133±16,3	a	44±5,1	b	52,6±7,5	b	43,8±6,5	b	77,3±7,3	b
Leptin (ng/ml)	2,8±0,5		2,6±1,1		1,1±0,4		2,1±0,3		1,2±0.6	
Hepatic (%)										
LPC	nd		nd		nd		nd		nd	
SM	0,9±0,0	a	1,0±0,0	a	0,5±0,0	b	0,7±0,2	ab	0,4±0,1	ab
PC	25,3±0,6	a	26,9±1,2	ab	30,6±0,6	b	28,5±0,7	ab	26,9±1,1	ab
PS	2,3±0,0	a	1,8±0,2	a	3,3±0,1	b	2,7±0,3	ab	2,9±0,2	ab
PG	5,5±0,1		5,3±0,3		5,3±0,3		4,5±0,5		5,6±0,4	
PE	17,4±0,3	a	18,1±0,6	a	16,1±0,5	ab	17,6±1,6	ab	13,5±0,5	b
PLE	0,8±0,2		0,4±0,0		0,3±0,1		0,3±0,0		0,3±0,1	
DG	1,7±0,3	a	0,9±0,2	b	0,6±0,1	b	1,8±0,1	a	1,7±0,2	a
CHO	14,7±0,7	bc	17,1±0,3	d	15,6±0,4	cd	13,2±0,2	b	11,3±0,7	a
FFA	7,9±0,5	b	7,1±0,9	b	1,8±0,3	a	2,5±0,5	a	2,0±0,3	a
TG	8,3±0,4	a	7,0±0,4	a	12,1±0,6	bc	11,5±1,0	bc	16,4±3,0	c
CE	4,1±0,3	a	4,6±0,1	a	6,0±0,1	b	6,5±0,1	b	9,5±1,1	c
										
TPL	63,2±0,6		62,9±1,7		63,7±0,9		64,6±0,7		59±2,8	
TNL	36,8±0,6		37,1±1,7		36,3±0,9		35,4±0,7		41±2,8	

Euthyroid testis-intact controls (INTACT) and hypothyroid-orquidectomized (TXOX) rats were performed as described in Material and Methods. Long-term administration of E2 (TXOXE2) or vehicle (VEH) to TXOX rats was performed for 20 days. Then, VEH (TXOX), E2 (TXOXE2), GH (TXOXGH) or E2 plus GH (TXOXE2GH) replacements were performed for additional 7 days. On PND90, the animals were sacrificed and serum and hepatic lipid classes were measured. Data are expressed as mean ± SEM for serum (n = 5) or hepatic (n = 5) independent samples (different animals) and each independent sample was tested twice. Values represent weight percent of total lipid. Values were submitted to ANOVA followed by post hoc Tukey's test. Values in the same row with different lowercase letters are significantly different with P<0.05.

**Table 2:** Effects of hormonal replacement on fatty acid composition from liver total lipids.

TOTAL LIPIDS	INTACT		TXOX		TXOXE2		TXOXGH		TXOXE2GH	
	mean ± SEM		mean ± SEM		mean ± SEM		mean ± SEM		mean ± SEM	
Fatty acids										
C 14: 0	0,5±0,1	ab	0,5±0,1	b	0,3±0,0	a	0,5±0,0	ab	0,3±0,0	ab
C 16: 0	19,8±0,5	ab	20,2±0,6	b	18,5±0,1	a	19,1±0,3	ab	18,5±0,6	a
C 16:1 n-9	0,3±0,0		0,4±0,0		0,3±0,0		0,4±0,0		0,3±0,0	
C 16: 1 n-7	2,2±0,4		1,4±0,1		1,6±0,1		1,5±0,3		1,7±0,2	
C 18: 0	13,2±0,2	a	15,8±0,4	c	14,5±0,2	b	14,7±0,2	b	12,7±0,3	a
C 18: 1 n-9	10,6±0,5	a	10,3±0,4	a	12±0,5	ab	10,4±0,5	a	13,9±0,6	b
C 18: 1 n-7	5,6±0,4	b	2,6±0,3	a	2,8±0,1	a	3,0±0,2	a	2,9±0,1	a
C 18: 2 n-6	15,8±0,9		18,2±0,7		19,1±0,3		17,1±0,9		18,7±0,9	
C 18: 3 n-6	0,2±0,0	a	0,8±0,1	b	1,4±0,1	c	0,6±0,0	b	1,3±0,3	abc
C 18: 3 n-3	0,3±0,0		0,3±0,0		0,4±0,0		0,3±0,0		0,3±0,1	
C 18: 4 n-3	0,0±0,0	a	0,0±0,0	ab	0,1±0,0	b	0,0±0,0	ab	0,1±0,0	b
C 20: 3 n-6	0,7±0,0	b	1,2±0,2	c	0,4±0,0	a	0,8±0,0	b	0,5±0,0	a
C 20: 4 n-6	19±0,9	ab	18,1±0,2	a	17,5±0,2	a	20,2±0,4	b	18,8±1,4	ab
C 20: 5 n-3	0,2±0,0	a	0,4±0,1	b	0,4±0,0	b	0,3±0,1	ab	0,2±0,1	ab
C 22: 4 n-6	0,8±0,1		0,5±0,0		0,7±0,1		0,8±0,1		0,6±0,1	
C 22: 5 n-6	1,0±0,2		0,5±0,0		1,0±0,1		0,8±0,1		0,8±0,1	
C 24: 0	0,4±0,0	a	0,6±0,0	b	0,6±0,0	b	0,6±0,1	b	0,5±0,1	ab
C 22: 6 n-3	4,4±0,2	b	3,4±0,2	a	4,0±0,0	ab	4,4±0,3	b	4,2±0,1	b
Totals										
SFA	35,3±0,3		38,7±0,8		35,2±0,2		36,1±0,4		33,2±0,6	
MUFA	19,6±1,1	b	17,3±1,9	a	17,3±0,4	ab	16,1±0,9	a	19,5±0,7	b
PUFA	43,7±1,5		43,2±2,0		46,9±0,3		47,0±10,9		46,9±0,8	
VLCPUFA	27±0,7		24,5±1,2		25,6±0,4		28,5±0,7		26,1±1,6	

Euthyroid testis-intact controls (INTACT) and hypothyroid-orquidectomized (TXOX) rats were performed as described in Material and Methods. Administration of E2 (TXOXE2) or vehicle (TXOX) to TXOX rats was performed for 20 days. Then, VEH (TXOX), E2 (TXOXE2), GH (TXOXGH) or E2 plus GH (TXOXE2GH) replacements were performed for additional 7 days. On PND90, animals were sacrificed and the liver extracted for fatty acid analyses as described in Material and Methods. Data are expressed as mean ± SEM for 5 independent samples (different animals) and each independent sample was tested twice. Values represent weight percent of total lipid. Values were submitted to ANOVA followed by post hoc Tukey's test. Values in the same row with different lowercase letters are significantly different with P<0.05.

**Table 3:** Effects of hormonal replacement on fatty acid composition from hepatic neutral lipids.

NEUTRAL LIPIDS	INTACT		TXOX		TXOXE2		TXOXGH		TXOXE2GH	
	mean ± SEM		mean ± SEM		mean ± SEM		mean ± SEM		mean ± SEM	
Fatty acids (%)										
C 14: 0	1,0±0,1	b	1,0±0,1	b	0,5±0,0	a	0,9±0,1	ab	0,6±0,1	ab
C 16: 0	25,2±0,8	b	25,4±1,0	b	22,2±0,4	ab	22,9±0,6	ab	21,6±1,1	b
C 16:1 n-9	0,6±0,1	ab	0,7±0,0	b	0,5±0,0	a	0,7±0,0	ab	0,5±0,0	ab
C 16: 1 n-7	4,1±0,8		2,5±0,1		2,8±0,2		2,8±0,5		3,3±0,6	
C 18: 0	4,3±0,3	ab	7,7±0,7	c	4,2±0,2	ab	5,2±0,5	b	3,3±0,3	a
C 18: 1 n-9	22,1±1,0	b	18,3±0,8	a	22,6±0,8	b	20,6±1,0	ab	26,1±0,3	c
C 18: 1 n-7	6,3± 0,7	b	3,3±0,3	a	3,1±0,1	a	3,5±0,3	a	3,7±0,2	a
C 18: 2 n-6	20,9±1,7	b	22,3±1,3	ab	25,2±0,8	a	23,3±1,7	ab	25,1±0,8	a
C 18: 3 n-6	0,2±0,0	a	1,1±0,1	b	2,3±0,1	c	1,1±0,1	b	1,8±0,5	abc
C 18: 3 n-3	0,7±0,1		0,7±0,1		0,8±0,0		0,7±0,1		0,8±0,1	
C 18: 4 n-3	0,0±0,0	a	0,1±0,0	abc	0,3±0,0	b	0,1±0,0	abc	0,2±0,0	c
C 20: 3 n-6	0,4±0,0	b	0,6±0,1	c	0,3±0,0	a	0,6±0,0	bc	0,5±0,1	bc
C 20: 4 n-6	5,6±0,8	a	8,6±0,3	b	7,3±0,3	ab	7,8±0,5	ab	6,0±1,2	ab
C 20: 5 n-3	0,3±0,0		0,4±0,1		0,5±0,1		0,5±0,2		0,3±0,1	
C 22: 4 n-6	1,0±0,2		0,5±0,1		0,7±0,1		1,2±0,2		0,7±0,1	
C 22: 5 n-6	0,6±0,1	a	0,3±0,1	a	0,4±0,1	ab	0,6±0,0	b	0,3±0,0	a
C 22: 6 n-3	1,5±0,2	b	1,2±0,1	ab	1,1±0,0	ab	1,4±0,2	b	0,8±0,1	a
C 24: 0	0,5±0,1	a	1,0±0,1	b	0,9±0,1	b	0,9±0,1	b	0,5±0,2	a
Totals										
SFA	32,1±0,8	b	36,5±1,2	c	28,9±0,3	ab	31,2±1,2	b	27,1±0,7	a
MUFA	34,4±2,3	b	25,8±1,1	a	29,8±0,8	ab	28,5±1,7	ab	34,6±0,8	b
PUFA	32,5±3,0		37,1±1,6		40,5±0,9		39,2±2,1		37,8±1,2	
VLCPUFA	10,1±1,4		12,6±0,4		11,2±0,5		13,0±0,7		9,5±1,4	

Euthyroid testis-intact controls (INTACT) and hypothyroid-orquidectomized (TXOX) rats were performed as described in Material and Methods. Administration of E2 (TXOXE2) or vehicle (TXOX) to TXOX rats was performed for 20 days. Then, VEH (TXOX), E2 (TXOXE2), GH (TXOXGH) or E2 plus GH (TXOXE2GH) replacements were performed for additional 7 days. On PND90, animals were sacrificed and the liver extracted for fatty acid analyses as described in Material and Methods. Data are expressed as mean ± SEM for 5 independent samples (different animals) and each independent sample was tested twice. Values represent weight percent of total lipid. Values were submitted to ANOVA followed by post hoc Tukey's test. Values in the same row with different lowercase letters are significantly different with P<0.05.

E2 treatment in TXOX rats brought about an important reduction in total SFA compared with the TXOX group, this effect was due to the significant reduction in 14:0 (myristic acid), 16:0 (palmitic acid), and 18:0 (stearic acid) from both total (Table 2) and neutral (Table 3) lipids. In parallel to these changes, FFA as a lipid class and DG were dramatically reduced by more than 75% and 38%, respectively, whereas TG increased significantly by 72% in comparison to TXOX rats. It is worth mentioning that E2 did not alter VLCPUFA metabolism in terms of the levels of 20:4n-6, 20:5n-3, and 22:6n-3, which remained similar to the values for neutral and total lipids in the TXOX group. However, E2 increased the levels of the essential n-6 precursor 18:2n-6 (but not the essential n-3 precursor 18:3n-3) in neutral lipids. Noticeably, E2 treatment partly reversed the effects of hypothyroidism on MUFAs. Thus, E2 significantly increased levels of 18:1n-9 (and also those of 16:1n-7 acid though to a lower extent) in total and neutral lipids (Table 2 and 3, respectively), which, together with the reductions in 16:0 and 18:0, point to an upregulation of stearoyl-CoA desaturase (Scd) genes in response to E2. Overall, these changes indicate that E2 stimulates β-oxidation of SFA in the liver while it increases the depots of n-6 polyunsaturated precursors in TG. Regarding other lipid classes, namely, CHO, CHO esters (CE), and phospholipids, we observed that E2 slightly reduces hepatic CHO levels (yet not statistically significant) compared with TXOX but induces a significant increase in the formation of CE (32% and 47% over TXOX and INTACT groups, respectively). This effect on CE formation is independent of hypothyroidism but totally determined by E2 and likely reflects an up-regulation of acyl:cholesterol acyltransferase (ACAT) activity (see discussion).

To some extent, GH administration to TXOX rats resembled the effects of E2 on hepatic lipid composition described above. Thus, GH increased TG and decreased FFA hepatic contents compared with TXOX rats. However, the levels of DG were notably increased by GH treatment in the TXOX group compared with the vehicle- or E2-treated TXOX group (Table 1). TG levels were also increased in serum from GH-treated rats (Table 1). Compared with the TXOX group, the hepatic levels of 18:0, 16:0 and total SFA in neutral lipids (Table 3) were reduced in GH-treated animals. Interestingly, unlike in E2-treated animals, the levels of 18:0 and 16:0 in neutral and total lipids in GH-treated rats approached those observed in INTACT animals. Taken together, these data suggest that GH induces a lipogenic effect in hypothyroid animals by mobilizing SFAs as FFA→DG→TG. Furthermore, when compared with vehicle- or E2-treated TXOX group, GH treatment induced a significant increase in 20:4n-6 and 22:6n-3 in total lipids (Table 2), which is reflected in the high levels of VLCPUFA observed in this group of animals. Because these later effects on LCPUFA were not observed in neutral lipids, the results point to a significant effect of GH on phospholipid remodeling, though the effect of E2 in modulating phospholipid acylation-reacylation has been established [34], similar effects induced by GH or by the combined treatment E2+GH represent a novel finding. Finally, CHO levels in the GH-treated TXOX group were returned to values observed in the INTACT group, but as in the E2-treated TXOX group, an increased CE level was present in response to GH (Table 1).

In the presence of E2, GH gave rise to a complex hepatic lipid phenotype. Thus, total SFA, 14:0, 16:0, and 18:0 in total lipids (Table 2), were reduced compared with TXOX and reached the levels observed in INTACT animals. In neutral lipids (Table 3), however, the contents of these fatty acids were significantly lower than in TXOX. Among VLCPUFA, AA was also decreased well below TXOX animals to values similar to those found in the INTACT group in neutral lipids (Table 3), while as in the TXOXGH group, DHA was significantly increased in the phospholipids of the TXOXE2GH group to achieve the levels in INTACT animals. A striking effect of the combined effects of E2 and GH is the complete restoration of MUFA levels from total and neutral lipids, an effect attributable to the increase in 18:1n9, likely through alteration of Δ9 desaturase expression. Interestingly, the combined E2+GH treatment gave raise to significantly higher MUFA levels compared to E2 and GH treatments individually. Given that MUFA levels of in GH-treated rats were identical to those in TXOX rats, and that E2 treatment increased MUFA (especially 18:1n-9) as compared for GH, the results suggest a permissive action of GH on E2 effect. We have described above that E2 and GH increased hepatic CHO and CE, but the combined effect of the two hormones seemed to be additive with regard to CE, because its levels doubled those found in INTACT animals and were approximately 30% higher than in the E2 and GH groups (Table 1). Conversely, in the presence of E2, GH reduced the hepatic CHO content compared not only to the TXOX group but also in relation to the E2- or GH-treated TXOX groups, indicating an antagonistic hormonal interaction. Finally, the significant increase in hepatic TG by GH in the presence of E2, with the largest content among all groups (>96% compared with the INTACT and >133% compared with the TXOX group), points to a substantial stimulation of hepatic lipogenesis by the combination of E2 and GH.

**Figure 2:**
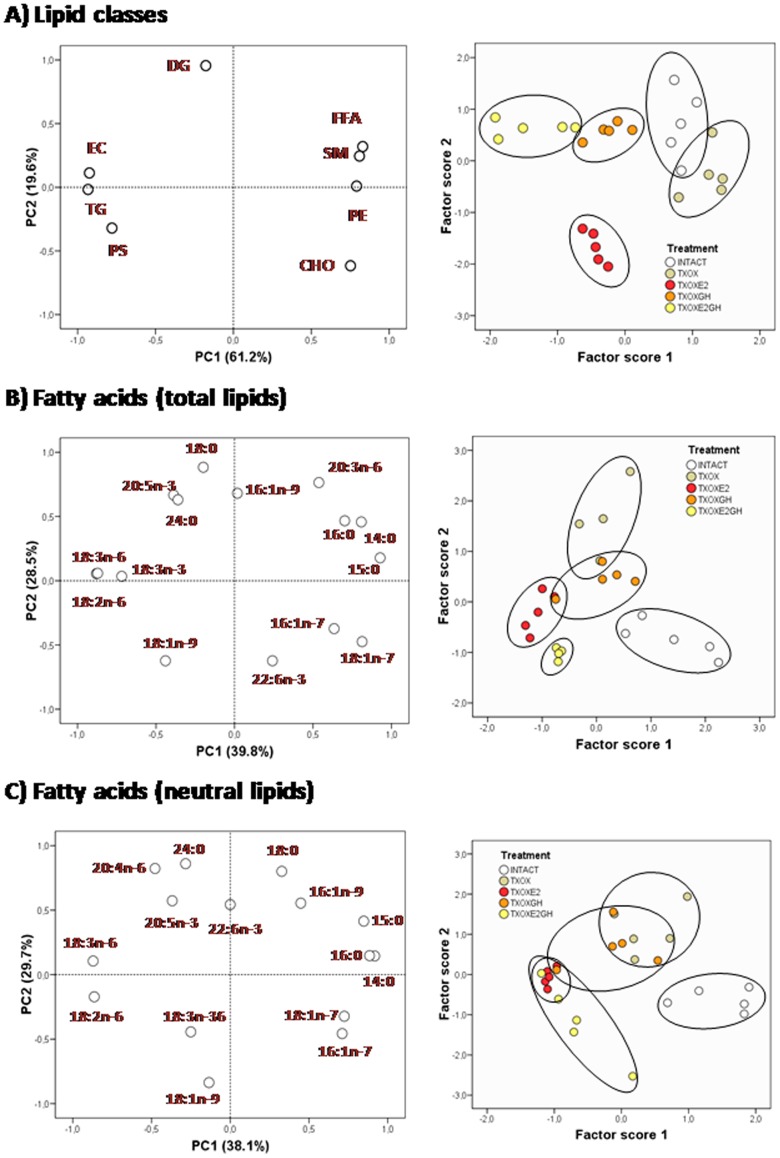
Principal component analysis for liver lipid composition. (A) Lipid classes, (B) Total lipids and (C) Neutral lipids. Left panels represent the factor loadings of principal components 1 (PC1) and 2 (PC2), and right panels the factor scores plots for PC1. Percent values in parentheses indicate the proportion of overall variance explained by each principal component. Each ellipse denotes a hormonal cluster. SM: sphingomyelin, PS: phosphatidylserine, PE: phosphatidylethanolamine, CHO: cholesterol, SE: sterol esters, FFA: free fatty acids, DG: diacylglycerols, TG: triacylglycerols.

Finally, we performed PCA to identify data that discriminate groups. Figure 2 shows the outcomes of PCA on lipid profiles for lipid classes and total and neutral lipids. For lipid classes (Fig. 2A), PC1 (principal component 1) was positively related to FFA, CHO, sphingomyelin (SM) and phosphatidylethanolamine (PE), and negatively correlated with CE and TG. On the other hand, for fatty acids from neutral (Fig. 2C) and total (Fig. 2B) lipids, PC1 was negatively related to C18 PUFA (18:2n-6, 18:3n-6 and 18:3n-3) in total lipids, and positively to saturated (14:0, 15:0 and 16:0) and n-7 MUFA. Therefore, PCA allowed a substantial simplification of lipid data for group discrimination. When we computed factor scores 1 and 2 from PC1 to obtain a group simplification for lipid classes (Fig. 2A) and fatty acids from total (Fig. 2B), and neutral lipids (Fig. 2C), these results allowed a neat discrimination between HRT conditions for all three analyses. INTACT rats are represented by a discrete cluster whereas the effects of E2 or GH on TXOX rats can be distinguished from the untreated TXOX group and display some degree of overlap with the combined E2 and GH treatments. Thus, in contrast with somatic growth in which E2 clearly prevented GH actions, the quantitative lipid analysis displays a more complex picture of the molecular changes induced by the different hormonal treatments. The E2 and GH effects on lipid contents display significant similarities but also treatment-dependent specific effects, such those leading to the regulation of DG content by GH.

### Estradiol influences liver transcriptome in hypothyroid male rats

**Figure 3:**
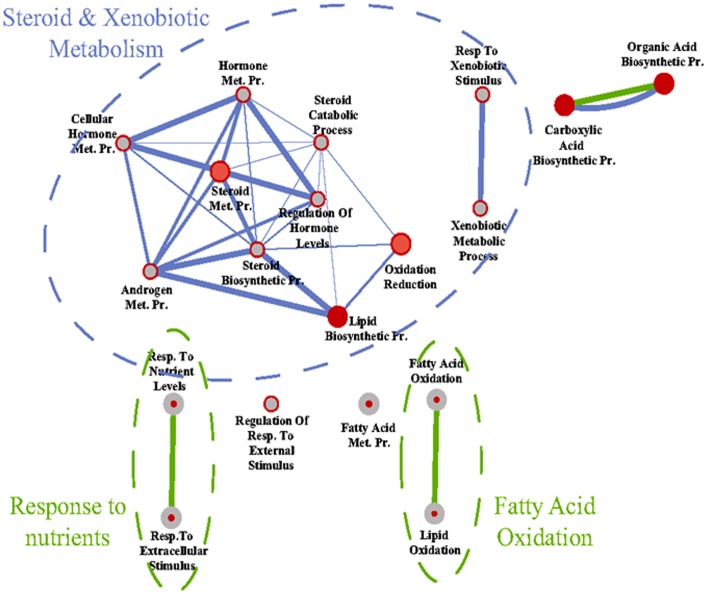
System biological network analyses of the effects of E2 on liver transcriptome in hypothyroid-orchidectomized rats. The genes differentially-expressed in the livers were identified by DNA microarrays as described under Material and Methods. Then, functional and system biological network analysis were performed on the basis of the GO enrichment of differentially-expressed genes in liver using DAVID, and the results depicted using Cytoscape. Node (inner circle) size corresponds to the number of genes up-regulated by E2; node border (outer circle) size corresponds to the number of genes down-regulated by E2; color of the node and border corresponds to the significance of the gene set for up or down regulated genes, respectively (dark red  =  significantly enriched, light red  =  enriched no significantly; grey  =  absent); edge size corresponds to the number of genes that overlap between the two connected gene sets. Green edges correspond to shared up-regulated genes and blue edges correspond to shared down-regulated genes.

Next, we performed a genome wide gene expression analysis to better understand the influence of E2 on liver physiology. This experiment identified 634 genes that were differentially regulated in TXOX rats after E2 treatment (Table S2). Next, we identified the active biological processes from expression profiles by GO enrichment analysis [40] and system biological network [41] analysis. GO enrichment analysis of the 352 genes up-regulated by E2 revealed a significant over-representation of genes related to fatty acid metabolism whereas among the 282 down-regulated genes, over-representation of genes involved in steroid and xenobiotic metabolism was observed (Fig. 3). Accordingly, the genes up-regulated by E2 were clustered in cellular pathways (KEEG) related to the PPARα signaling (P = 1,4E-05; Bonferroni  = 0.002) and biosynthesis of unsaturated fatty acids (P =  8,2E-04; Bonferroni  = 0.02). Among the genes up-regulated by E2 we observed PPARα and PPARα target genes [48], [50] such as CYP4A1, CYP4A3, FGF21, carnitine palmitoyltransferase 2 (CPT-2), Scd1, LCFA-CoA ligase 4, acyl-CoA oxidase (ACOX1), acyl-CoA synthetase (ACS), fatty acid translocase (FAT/CD36), angiopoietin-like 4 (ANGPTL4), ELOVL5, and BAAT. The fatty acid desaturases FAD6 and FAD1 were also upregulated by E2. In contrast, genes involved in the metabolism of C21-steroid hormones (P = 1,6E-05; Bonferroni  = 0.001), metabolism of xenobiotic by cytochrome 450 (P =  1,2E-04; Bonferroni  = 0.006), glutathione (P = 0.002; Bonferroni  = 0.04), and androgen and estrogen (P = 0.002; Bonferroni  = 0.05) were significantly down-regulated by E2 [i.e., aldo-keto reductase 1D1 (Akr1d1), epoxide hydrolase 1; glutathione S-transferases (GST) (mu 2, mu 3, mu 4, mu 7, pi 1), CYP3A18, CYP2C23, CYP2E1, CYP17A1, 3β-HSD, 11β-HSD1, and estrogen sulfotransferase (SULT1E)]. Overall, these results reveal an extensive re-programing of liver's transcriptome by long-term E2 treatment of TXOX rats, particularly genes involved in the metabolism of fatty acids and endo-xenobiotics.

### Estradiol influences GH-regulated liver transcriptome in hypothyroid male rats

**Figure 4:**
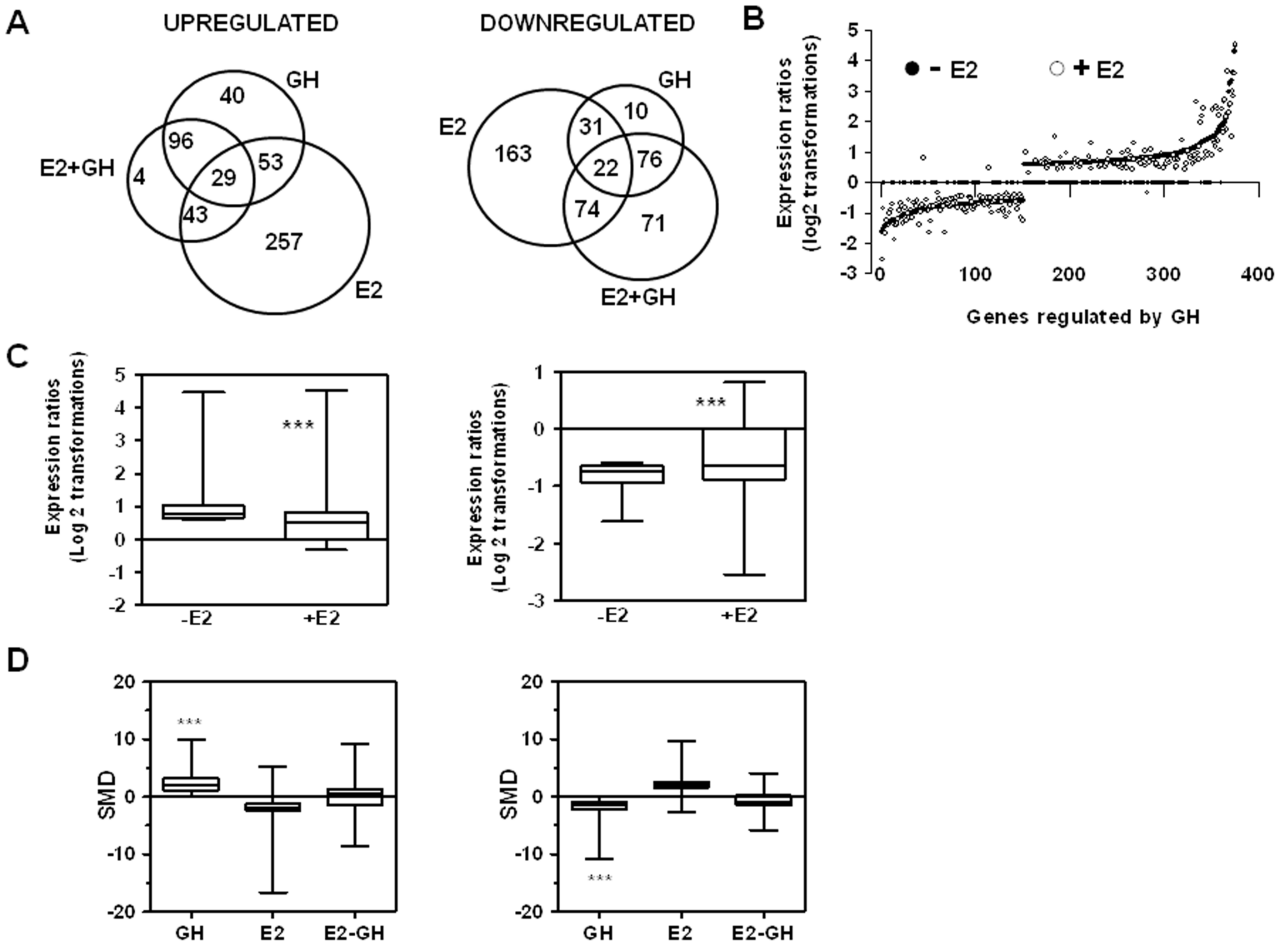
E2 influences the gene expression profiling regulated by GH in hypothyroid-orchidectomized rat liver. Hypothyroid-orchidectomized (TXOX) rats were injected with GH for 7 days in the absence (−E2) or in the presence (+E2) of E2. Differently expressed genes in the livers were identified by DNA microarrays as described under Material and Methods. (A) The number of genes regulated by GH in the absence of E2, by E2, or GH in the presence of E2. The overlapping areas show genes for which expression was altered by GH in the absence or presence of E2. (B) Individual genes are arranged along the X axis according to the value order of decreases and increases in gene expression measured in GH-treated TXOX rats in the absence of E2. The Y axis shows the log 2 ratio of the transcript signals in GH-treated TXOX rats in the absence (−E2) and in the presence (+E2) of E2. (C) Box plot shows a statistical evaluation of the differences in the mean expression changes induced by GH in the absence (−E2) or in the presence (+E2) of E2 for the set of genes induced and repressed by GH treatment in the absence of E2. (D) SAM multiclass analysis was performed to identify GH regulated genes whose mean expression (SMD) values were significantly different from E2 or E2 plus GH-treated TXOX rats. In Box plots, the lines connect the medians, the boxes cover the 25th to 75th percentiles, and the minimum and maximum values are shown by the ends of the bars. ***, *P*<0.001.

Finally, we performed a genome wide gene expression analysis to better understand the interplay between E2 and GH in liver. We first defined the gene expression changes induced by GH replacement in TXOX rats (Table S3). Second, we analyzed the similarities in gene expression changes induced by treatment with E2 or GH in TXOX rats and identified 869 significantly (FDR <5%) regulated transcripts in both cases. A Spearman rank correlation test comparing the E2 and GH effects on these genes yields a positive correlation (r = 0.6287; *P*<0.0001) indicative of strong similarities between E2 and GH effects in the TXOX liver. Accordingly, 94% of these genes were regulated by E2 and GH in the same direction. Third, we obtained transcription profiles from TXOX rats simultaneously treated with both E2 and GH (Table S4). A comparative analysis of genes with altered expression levels in TXOXGH or TXOXE2GH groups revealed considerable reduction in the presence of E2 of the effects induced by GH in TXOX rats (Fig. 4A–B). This was a general phenomenon that affected a large fraction of GH-regulated genes. Accordingly, in the absence of E2, the average expression changes (log_2_) across four independent hybridizations were 0.98±0.04 and −0.82±0.02 for GH-induced (Fig. 4C) and GH-repressed (Fig. 4D) genes, respectively, whereas the average fold regulation for the same set of genes in the presence of E2 was 0.60±0.05 (Fig. 4C) and −0.61±0.04 (Fig. 4D). These differences were significant (*P*<0.001) and demonstrate an inhibition by E2 of the hepatic response to GH treatment. Finally, SAM multiclass analysis [38] identified genes regulated by GH whose mean expression values were significantly different from those in E2- or E2 plus GH-treated TXOX rats (Fig. 4E and 4F and Table S5) (e.g., Snapc2, SLC13A2, Apo-H, TNFR, SULT1E1, CYP2C11, CYP2C12, EGFR, Hsd3b6, SPI, alpha 2u-globulin, FTO, Acacb, PPARα, ACOX1, SOCS5, Akr1c14).

**Figure 5:**
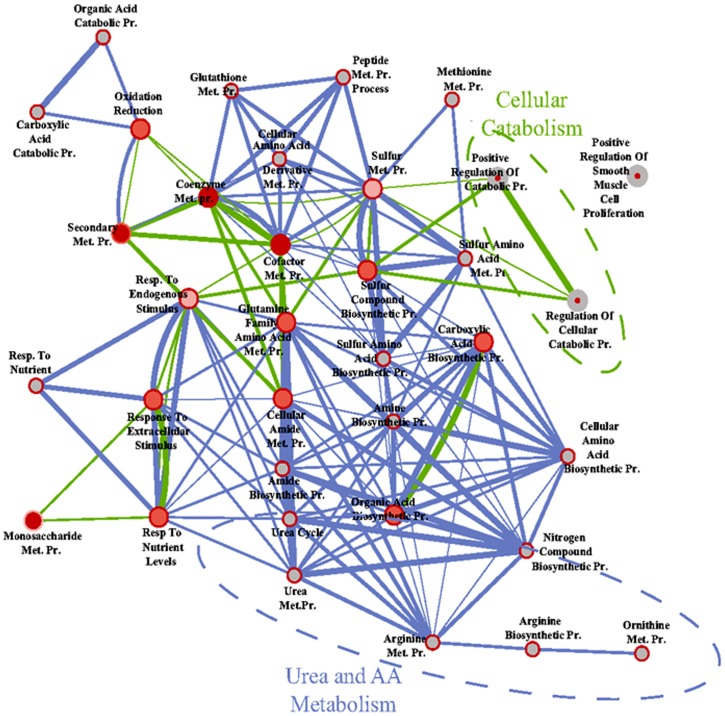
System biological network analyses on GH effects on liver transcriptome in hypothyroid-orchidectomized rats. The differentially-expressed genes in the livers were identified by DNA microarrays as described under Material and Methods. Then, functional and system biological network analysis were performed on the basis of the GO enrichment of differentially-expressed genes in liver using DAVID, and the results depicted using Cytoscape. Node (inner circle) size corresponds to the number of genes up-regulated by GH; node border (outer circle) size corresponds to the number of genes down-regulated by GH; color of the node and border corresponds to the significance of the gene set for up or down regulated genes, respectively (dark red  =  significantly enriched, light red  =  enriched no significantly; grey  =  absent); edge size corresponds to the number of genes that overlap between the two connected gene sets. Green edges correspond to shared up-regulated genes and blue edges correspond to shared down-regulated genes.

**Figure 6:**
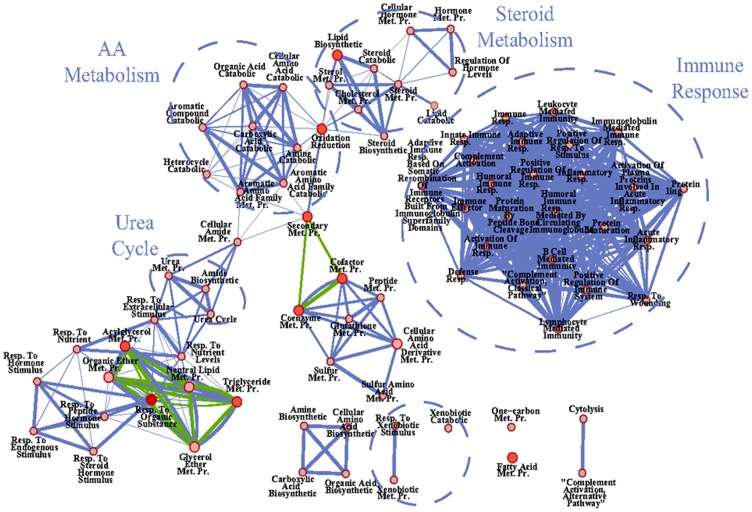
System biological network analyses on GH effects on liver transcriptome in E2-treated hypothyroid-orchidectomized rats. The differentially-expressed genes in the livers were identified by DNA microarrays as described under Material and Methods. Then, functional and system biological network analysis were performed on the basis of the GO enrichment of differentially-expressed genes in liver using DAVID, and the results depicted using Cytoscape. Node (inner circle) size corresponds to the number of genes up-regulated by E2-GH; node border (outer circle) size corresponds to the number of genes down-regulated by E2-GH; color of the node and border corresponds to the significance of the gene set for up or down regulated genes, respectively (dark red  =  significantly enriched, light red  =  enriched no significantly; grey  =  absent); edge size corresponds to the number of genes that overlap between the two connected gene sets. Green edges correspond to shared up-regulated genes and blue edges correspond to shared down-regulated genes.

Functional analysis [40] revealed that the biological processes over-represented in the list of genes up-regulated by GH in TXOX liver, were connected with the positive regulation of cellular catabolism (e.g., GCLC, GCK, ABHD5, IGF-I, APOC2, HSP90AB1) whereas the metabolism of aminoacids and urea (e.g., CTH, ARG1, ASS1, OTC, ASL, CPS1, MAT1A, MTR, BHMT, PAH) was significantly down-regulated (Fig. 5). When these changes were mapped to cellular pathways (KEEG), we confirmed a significant connection with the metabolism of aminoacids and urea, metabolism of xenobiotics by P450, or PPARα signaling (Table 4). In contrast to E2, GH repressed the PPARα signaling pathway. Notably, in the presence of E2, the biological functions and network regulated by GH varied notably (Fig. 6). Thus, whereas GH still reduced the expression of genes involved in urea and amino acid metabolism in the presence of E2 [e.g., carbamoyl-phosphate synthetase-1 (CPS1), ornithine carbamoyltransferase (OTC); argininosuccinate synthase (ASS1)], it was no longer able to induce the expression of genes related to cell catabolism [e.g., glutamate-cysteine ligase (GCLC) and APOC2] (Table 5 and Table S4). In contrast, genes involved in the metabolism of steroids and xenobiotics (e.g., Gst2, Gst-m3, Gst-m4, EPHX1, CYP2E1, Gst-p1, Gst-m7) downregulated by E2 but not by GH alone are even further inhibited by the combined treatment. Finally, when E2 was present, GH exerted a significant influence on immune system response (Fig. 6). In particular, the mRNA expression levels of genes involved in complement activation, lymphocyte immune response, wounding or acute inflammatory response were significantly reduced (e.g., C9, C4a, AHCY, ASS1, MASP1, C5, CLU, CST3, A1I3, C4BPB, SERPING1, C1S, C4BPA, C8B, SDC1, IL10RB, IldR1, SLC7A2, PIGR, sialomucin, IGFBP1, or CFI) (Table 5 and Table S4). Taken together, these data indicate that E2 and GH may be effective regulators of immune system contributing to the maintenance of immune homeostasis under conditions of immunological stress to reduce the susceptibility to stress-induced disease by negative immunoregulators.

**Table 4:** Cellular pathways regulated by GH in hypothyroid-orchidectomized rat liver.

Pathway	up-regulated genes	down-regulated genes	*P*	B
Arginine and proline metabolism	GLUD-1	CPS-1, mitochondrial; OCT; ASS-1; arginase; aminotransferase	2,6E-05	3,1E-03
				
Urea cycle and metabolism of amino groups		CPS-1, mitochondrial; OCT; ASS-1; arginase	1,3E-03	3,8E-02
				
Glycine, serine and threonine metabolism	choline kinase α; glycyl-tRNA synthetase	serine dehydratase; betaine-homocysteine methyltransferase; cystathionase; glycine N-methyltransferase	1,6E-03	3,7E-02
				
Drug metabolism - Metabolism of xenobiotics by P450	CYP2C11; CYP2D2	CYP2A1; CYP2C23; ADH 1; GST (k1 and A3)	1,0E-03	4,0E-02
				
Methionine metabolism	methionine-tRNA synthetase	methionine adenosyltransferase I, alpha; betaine-homocysteine methyltransferase; cystathionase; 5-methyltetrahydrofolate-homocysteine methyltransferase	9,4E-04	5,4E-02
Retinol metabolism	CYP2C11	CYP2A1; CYP2C23; ADH 1; Dgat2	4,8E-03	9,1E-02
Caffeine metabolism	NAT-2	CYP2A1; urate oxidase	1,1E-02	1,7E-01
PPAR signaling	CPT-2; ANGPTL4	CYP27A1; CYP8B1; PEPK 1; ACOX-1	1,3E-02	1,8E-01
Cysteine metabolism	LDH-A	serine dehydratase; cystathionase	2,8E-02	2,9E-01
p53 signaling pathway	IGF-1; GADD-inducible, α and γ	cyclin B3; sestrin 1	4,4E-02	3,9E-01

The genes differentially-expressed in the livers were identified by DNA microarrays as described under Material and Methods. DAVID was used to identify the hepatic KEEG pathways that were affected by GH. The table shows pathway name, up- and down-regulated genes, P value, and corrected P value (Benjamini). Abbreviations: carbamoyl-phosphate synthetase-1 (CPS1); ornithine carbamoyltransferase (OTC); argininosuccinate synthase (ASS1); acyl-CoA oxidase 1, palmitoyl (ACOX-1); angiopoietin-like 4 (ANGPTL4); diacylglycerol O-acyltransferase 2 (Dgat2); glutamate dehydrogenase 1 (GLUD-1); Lactate dehydrogenase A (LDH-A); stearoyl-CoA desaturase (Scd); estrogen sulfotransferase (SULT).

**Table 5:** E2 influences the cellular pathways regulated by GH in hypothyroid-orchidectomized rat liver.

Pathway name	up-regulated genes	down-regulated genes	*P*	B
Drug metabolism-Metabolism of xenobiotics by P450	FMO 3; CYP2C11; CYP2C24; NAT	CYP2A1; CYP2C23; CYP2E1; UGT (2B17; 2B36 and 1A); ADH 1; GST (mu 2; mu 3; mu 4; mu 7; pi 1; A3); uridine phosphorylase 2; epoxide hydrolase 1, microsomal	9,6E-10	1,2E-07
				
Complement and coagulation cascades	fibrinogen α chain	complement [C1; C4; C5; C8; C9]; serine (or cysteine) peptidase inhibitor; mannan-binding lectin serine peptidase 1	6,5E-07	2,8E-05
				
Retinol metabolism	CYP2C11; CYP2C24	CYP2A1; CYP2C23; CYP2A1; UGT (2B17; 2B36; and 1A); ADH A1; retinal pigment epithelium 65	1,0E-06	3,3E-05
				
Nitrogen metabolism	GLUD 1	CPS-1, mitochondrial; carbonic anhydrase (3 and 8); cystathionase; histidine ammonia lyase	1,7E-04	4,5E-03
PPAR signaling	CPT-1a; CPT-2; ANGPTL4	CYP7A1; CYP8B1; PEPK 1; CD36; LCA-CoA synthetase 1; Scd2	2,6E-04	5,7E-03
Fatty acid metabolism	CPT-1a; CPT-2	glutaryl-CoA dehydrogenase; ADH 1; enoyl CoA hydratase, short chain, 1, mitochondrial; ADH 2 (mitochondrial); LCA-CoA synthetase 1	4,4E-04	8,0E-03
				
Arginine and proline metabolism	GLUD1; spermidine synthase	CPS-1, mitochondrial; OCT; ASS-1; aminotransferase; ADH-2	1,5E-03	2,2E-02
Steroid hormone biosynthesis		CYP7A1; UGT (2B17; 2B36; 1A); SULT; HSD11B1	3,4E-03	4,3E-02
Tryptophan metabolism		tryptophan 2,3-dioxygenase; glutaryl-CoA dehydrogenase; kynurenine 3-monooxygenase; catalase; enoyl CoA hydratase, short chain, 1, mitochondrial; ADH-2	3,4E-03	4,3E-02
				
Glutathione metabolism	spermidine synthase	isocitrate dehydrogenase 1 (NADP+), soluble; GST (A3; mu2; mu 3; mu 4; mu 7; pi 1)	6,6E-03	7,4E-02

The genes differentially-expressed in the livers were identified by DNA microarrays as described under Material and Methods. DAVID was used to identify the hepatic KEEG pathways that were affected by GH in the presence of E2. The table shows pathway name, up- and down-regulated genes, P value, and corrected P value (Benjamini). Abbreviations: carbamoyl-phosphate synthetase-1 (CPS1); argininosuccinate synthase (ASS1); acyl-CoA oxidase 1, palmitoyl (ACOX-1); angiopoietin-like 4 (ANGPTL4); diacylglycerol O-acyltransferase 2 (Dgat2); glutamate dehydrogenase 1 (GLUD1); ornithine carbamoyltransferase (OTC); stearoyl-CoA desaturase (Scd); estrogen sulfotransferase (SULT).

## Discussion

In this study, we show that E2 and GH replacements in hypothyroid male rats have a significant impact on lipid content and transcriptome in the liver and that E2 exerts a marked influence on GH-regulated endocrine, metabolic, immune, and gender specific responses in the liver.

Hypothyroidism impaired body weight gain and decreased circulating levels of IGF-I and biological markers of GH-STAT5b signaling activity in the liver (i.e., mRNA levels of IGF-I, ASL, SOCS2, CIS, and CYP2C11) [51]. These changes were totally or partially restored by intermittent GH administration to TXOX rats. However, the effects of GH were largely prevented by E2 which is in line with the negative effects of estrogens on continuously GH administration in hypophysectomized female rats [52]. The positive effects of E2 on hepatic SOCS2, CIS, and FGF21 transcripts (see Fig.1) suggest that E2 might prevent the activation of GH-STAT5b signaling in liver through induction of these negative regulators of GH signaling [14], [48]. Similarly, estrogen administration in humans can prevent the GH-induced increase in IGF-I, IGFBP-3, lipid oxidation, and protein synthesis [22], [23]. The effects of hypothyroidism on growth are associated, in part, with an increased hepatic amino acid catabolism and urea synthesis [53]. Biological network analysis shows that intermittent GH administration to TXOX rats causes a positive regulation of cellular catabolism, whereas the genes involved in the metabolism of amino acids and urea (i.e., OTC, ASS1, aminotransferases, and methyltransferases) are significantly down-regulated. This is in line with the positive effects of GH on nitrogen balance, which have been previously studied in hypophysectomized rats [54], [55], [56].

GH serves as an anabolic hormone that promotes lipolysis and prevents lipogenesis in adipose tissue, which increases the availability of FFA for energy expenditure [56]. E2 is also able to interfere with this process by preventing the induction of some genes related to fat utilization, such as ApoC2, which activates the enzyme LPL that hydrolyzes TG. Therefore, E2 actions in liver can impact the peripheral metabolic actions of GH.

Lipogenesis is often increased in situations of reduced energy expenditure such as hypothyroidism, GH deficiency, E2 deficiency, or aging [57]. Accordingly, our analysis of the hepatic lipid content revealed that TXOX rats contained significantly increased levels of total SFA compared to INTACT rats. E2 replacement did not modify the mRNA expression levels of key regulators of hepatic lipogenesis [i.e., Sterol regulatory element binding protein (SREBP)1c, acetyl-Co A carboxylase alpha (ACC), fatty-acid synthase (FAS)] [58], whereas it activated a PPARα transcriptional program that promotes fatty acid catabolism in liver [50], [59]. This was evidenced by the E2 increased expression of the PPARα gene itself and the PPARα target genes involved in the β/ω-oxidation of fatty acids (i.e., CTE-I, CPT-2, Fasd6, Fasd1, Fasd2, Scd1, ACOX1, ECH1, BAAT, FGF21, CYP4A1, CYP4A3) (Table S2). Accordingly, E2 replacement caused a significant reduction in SFAs. Overall these findings are indicative of a positive crosstalk between E2 and PPARα that is supported by multiple independent studies [2], [60], [61], [62]. Interestingly, despite the increased expression of genes involved in β-oxidation, we detected a significant increase in hepatic TG content in E2 treated TXOX rats, which is likely explained by effects on lipid transport. The first step of long chain fatty acids uptake is its translocation across the plasma membrane. Notably, E2 increased the transcription of several known PPARα activated genes encoding proteins that have been implicated in fatty acids uptake and activation such as CD36, ACSL4 and SLC27A5 (FATP5) [63], [64]. We have previously demonstrated that the fatty acid transporter CD36 is predominantly expressed in female rat livers and proposed that this sexual dimorphism depends on the GH secretion pattern, which can be influenced by E2 treatment. E2 also increased transcripts of the SLC27A5 gene which encodes FATP5, an fatty acid transporter that is an acyl-CoA synthetase (bile acid ligase) that catalyzes the conjugation of bile acids with amino acids before excretion into bile canaliculi [65]. Following fatty acids uptake, the first step for the intracellular use of long chain fatty acids is its esterification with CoA. This reaction is catalyzed by acyl-CoA synthetases such as ACSL4 which was also induced by E2 in TXOX rat liver. The produced acyl-CoAs are substrates for β-oxidation but also can prime the synthesis of TG, phospholipids, CE, and ceramides and therefore are also a primary source of signaling molecules [66]. The notion that E2 may regulate the formation of lipid signaling intermediaries is supported by the stimulation of fatty acids elongase-5 (Elovl5). Elovl functions with fatty acid desaturases to generate many of the long-chain PUFAs assimilated into cellular lipids (i.e., 20:4n-6 and 22:6n-3). However, it is worth mentioning that E2 administration did not alter VLCPUFA metabolism because the levels of 20:4n-6, 20:5n-3 and 22:6n-3 remained similar to values in the TXOX group. It has been reported that E2 might play a critical role in lipogenesis and Scd1 transcription [2], a gene that encodes a rate-limiting enzyme to generate MUFAs such as 18:1 n−9 and 16:1 n−7. Previous studies have reported that the absence of E2 or ERα in rats provoked a profound increase in lipogenesis and Scd1 transcription [67], which suggests that E2 inhibits Scd1 transcription. Interestingly, the antilipogenic effect of E2 therapy, while maintaining efficient TG export and reduced phospholipid transfer protein, has been reported to depend on hepatic ERα [13], [61]. Our study, however, shows that E2 increased the Scd1 gene expression and that this effect was paralleled by reduced hepatic content of 18:0 and increased of 18:1 n−9 (the main product of SCD reaction) contents, in total and, especially, in neutral lipids compared with TXOX animals, which indicates that E2 modulates SCD1 activity in TXOX liver. Surprisingly, E2 also downregulated Scd2 gene expression in TXOX rat livers. The significance of this opposed transcriptional regulation of Scd genes is unknown, but given that transcript levels of Scd1 are about 1800 times higher than that of Scd2 in the rat liver [68], changes in 18:1n-9 and 18:0 must be entirely attributed to variations in Scd1 gene expression. Overall, the changes in the lipid composition and gene expression profile seen in E2-treated TXOX rats support the finding that E2-PPARα functional interactions play a physiological role in the regulation of hepatic lipid metabolism.

E2 has the ability to reduce circulating CHO in women and in animal models fed on a high-fat diet [69]. However, E2 was unable to efficiently reverse hypercholesterolemia or hypotriglyceridemia in TXOX rats. This result may be due to the fact that E2 reduced expression levels of several transporters of CHO (and CE), including ApoB and ABCA1 in TXOX rats, which most likely contributed to maintaining an increased hepatic level CE. E2 may also induce intracellular CHO mobilization by modulating enzymes involved in CE and CHO synthesis and/or turnover [57], [70]. Distinct enzymes can catalyze the CHO to CE conversion in liver: lecithin:cholesterol acyltransferase (LCAT), which uses phosphatidylcholine (PC) as a source of acyl changes and ACAT, which uses acyl-CoA. Because the levels of lysophosphatidylcholine (LPC) were undetectable in all groups, our initial conclusion was that E2 stimulated the ACAT2 reaction to increase CE. However, we did not detect changes in the expression level of the ACAT gene, which did not discard posttranslational modification of enzymes in the CE cycle in the liver from E2-treated TXOX rats.

An increased level of hepatic CE, together with the increased TG and decreased FFA hepatic contents in GH-treated TXOX rats, resemble the effects of E2 on hepatic lipid composition and suggest that some effects of E2 might be GH mediated. A striking consequence of the combined replacement with E2 and GH is the complete restoration of MUFA levels from total and neutral lipids, an effect attributable to the increase in 18:1n9, likely through alteration of Δ9 desaturase expression. Moreover, GH and E2 increased hepatic CE and the combined effect of the two hormones were additive with regard to CE because its levels doubled those found in INTACT animals and were approximately 30% higher than in the E2 or GH groups which indicates a more efficient hepatic CHO metabolism. Accordingly, in the presence of E2, GH reduced hepatic CHO content compared not only to the TXOX group but also in relation to the E2- or GH-treated TXOX groups. The hepatic content of TG was, however, significantly increased by GH in E2-pretreated TXOX rats, which suggests that combined treatment by E2 and GH dramatically enhances lipogenesis. It is known that in contrast with its lipolytic effects in adipose tissue, GH exerts lipogenic actions in liver through stimulation of SREBP1, which is usually accompanied by increased hepatic TG (VLDL) secretion [56]. Indeed, our lipid profiling analysis suggested that intermittent GH administration to TXOX rats increased lipogenesis in the liver. However, in contrast to the effects of a continuous infusion of GH in hypophisectomized rats [55], intermittent GH administration to TXOX rats did not increase SERBP1, whereas several genes involved in fatty acids transport (e.g., FABP) and the biosynthesis of unsaturated fatty acids from 18:2n-6 and 18:3n-3 (e.g., fatty acid desaturases 4, 5 and 6) were induced. Interestingly, intermittent GH administration to TXOX rats down-regulated the expression of the lipin gene, an SREBP1c target gene, which is critical in the regulation of cellular levels of DG and TG and a key regulator of fatty acid oxidation in adipose tissue, skeletal muscle, and liver tissue [71]. These findings support the hypothesis that the female pattern of GH administration is a more efficient stimulus to induce lipogenic effects in the liver than the male pattern [72], [73]. Another mechanism whereby GH might promote lipogenesis in the liver is through the down-regulation of lipid oxidation. We have previously shown that continuous GH administration to hypophysectomized [55] and to old-intact [74] male rats inhibited PPARα. Accordingly, our lipidomic and genomic analysis showed that intermittent GH administration to TXOX rats also leads to down-regulation of the PPARα signaling pathway. In particular, GH represses the expression of PPARα itself, ACOX-1, CPT-1, FGF21, and several members of the CYP4A family, which are involved in fatty acid oxidation.

In summary, our study adds novel data that highlight the impact of subcutaneous E2 administration on liver physiology and its interplay with GH. These results highlight the role of E2 as a critical regulator of liver metabolism in mammals and add further weight to the hypothesis that E2 acts as an important regulator of GH actions in the liver. The E2-GH interplay in the liver is relevant because of the physiological roles that these hormones have in mammals and the widespread use of estrogen and estrogen-related compounds in human. Notably, this is the first study to demonstrate that hepatic lipid profiles are endowed with singular fingerprints that may be used to segregate different groups with altered hormone status. This includes different hormonal replacements (E2 or GH) that induced overlapping changes in gene expression. Therefore, liver lipid profiling can serve to identify cryptic hormone deficiencies or exposure to hormones or hormone-like substances.

## Supporting Information

Table S1 **Genes and primer sequences (5′- 3′) used for real-time PCR.**
(PDF)

Table S2 **Hepatic genes regulated by E2 in hypothyroid-orchidectomized rats.** The administrations of vehicle (TXOX) or E2 (TXOXE2) to TXOX rats were described in Material and Methods. Then, differently expressed genes in the livers were identified by DNA microarrays. The analysis is based on the SAM statistical technique and differentially-expressed genes were discovered using a FDR less than 5% and a mean ratio of log2>|0.58|. The table shows ENSEMBL gene ID, Unigene/Refseq, gene symbol, gene description, R (TXOXE2/TXOX), SD, and q (%).
(PDF)

Table S3 **Hepatic genes regulated by GH in hypothyroid-orchidectomized rats.** The administrations of vehicle (TXOX) or GH (TXOXGH) to TXOX rats were described in Material and Methods. Then, differently expressed genes in the livers were identified by DNA microarrays. The analysis is based on the SAM statistical technique and differentially-expressed genes were discovered using a FDR less than 5% and a mean ratio of log2>|0.58|. The table shows ENSEMBL gene ID, gene symbol, gene description, R (TXOXGH/TXOX), SD, and q (%).
(PDF)

Table S4 **Hepatic genes regulated by GH in E2-treated hypothyroid-orchidectomized rats.** The administrations of **v**ehicle (TXOX) or E2 plus GH (TXOXE2GH) in TXOX rats were described in Material and Methods. Then, differently expressed genes in the livers were identified by DNA microarrays. The analysis is based on the SAM statistical technique and differentially-expressed genes were discovered using a FDR less than 5% and a mean ratio of log2>|0.58|. The table shows ENSEMBL gene ID, gene symbol, gene description, R (TXOXE2GH/TXOX), SD, and q (%).
(PDF)

Table S5 **Hepatic genes regulated by GH whose mean expression values are different from those in E2- and E2 plus GH-treated hypothyroid-orchidectomized rats.** Hormonal replacements with GH (TXOXGH), E2 (TXOXE2) or E2 plus GH (TXOXE2GH) in TXOX rats were described in Material and Methods. Then, differently expressed genes in the livers were identified by DNA microarrays. SAM multiclass analysis was performed to identify GH regulated genes (TXOXGH) whose mean expression values were significantly different from E2 (TXOXE2)- or E2 plus GH (TXOXE2GH)-treated TXOX rats. The table shows ENSEMBL ID, Unigene/Refseq, gene symbol, gene description, R (mean expression), and q (%).
(PDF)
