# Supplementary material for: Lipid Profiling and Transcriptomic Analysis Reveals a Functional Interplay between Estradiol and Growth Hormone in Liver
Source: PLoS One. 2014 May 9;9(5):e96305. doi: 10.1371/journal.pone.0096305 (PMC4015979; doi:10.1371/journal.pone.0096305)
Supplement: Table S1 — Genes and primer sequences (5′- 3′) used for real-time PCR. (PDF) [file pone.0096305.s001.pdf]

Table S1. Gene names and primer sequences (5´- 3´) used for real-time PCR

Genbank Acc. Nº	Gene Product	Gene Symbol	Forward primer	Reverse primer
NM_178095.2	ATP-binding cassette, sub-family A (ABC1)	ABCA1	GGTAGTGTGGCCACTTTCGT	TCTGGGCCTGATGAAAAATC
NM_022193.1	Acetyl-coenzyme A carboxylase alpha	ACC1	TTGGCTGAGCGACTGGGCAC	TCTCCTGCATCCGGCCTGGG
NM_053922.1	Acetyl-Coenzyme A carboxylase beta	ACC2	CGGCTGCTGCTGGAAGCCAT	TGACCATGGCACGCACCGAC
NM_017340	Acyl CoA oxidase 1, peroxisomal (Palmitoyl-CoA oxidase)	AOX	TCGGGCAAGTGAGGCGCATT	AGCAACAGCATTGGGGCGGA
NM_013112	Apolipoprotein A-II	Apoa2	GGCAAGGATTTGATGGAGAA	ACAAGCAGAAAGTCGGGAGA
NM_019287	Apolipoprotein B	ApoB	CAGGGCACCAACCAGATAGT	GAGGGATCCAGAAAGAAGGG
NM_031804	Cytokine inducible SH2-containing protein	CIS	CGTACAGGAGGCCACGTAAT	CCACCCCAGCTACCTGTTTA
NM_031559.1	Carnitine palmitoyltransferase 1a	CPT1	GCAAGAGGAGTGAGACCCTG	TTAGCCCATTGGATGCTTTC
NM_001004085.2	carnitine acetyltransferase	CRAT	CCCAAAGTGCCGGAGCAGCA	GTGCCGGTCAAAGGCCTCCC
NM_178847	Cytochrome P450, family 27, subfamily a, polypeptide 1	Cyp27a1	ACCCTGCGCCTCTACCCTGT	GCAATCCTGCGACCCAGGCA
NM_019184.2	Cytochrome P450, family 2, subfamily c, polypeptide 11	Cyp2c11	TCGGATCCAGTGAACATCAA	ACTGCATGGGAATGTCACAA
NM_031572.1	Cytochrome P450, family 2, subfamily c, polypeptide 12	Cyp2c12	ACCAAAGGCTCACCCTGTGATCC	CCTGAAATGCAGGAGTGCTCACAA
NM_138514	Cytochrome P450, family 2, subfamily c, polypeptide 77	Cyp2c13	CCTTTCTCAGCAGGAAAACGGATGT	GCGCATCTGGAAAGTGGGAGG
NM_017158.1	Cytochrome P450, family 2, subfamily c, polypeptide 7	Cyp2c7	CATGGATCTCATTGGTGCAG	GGTTGGTCGGGACAAAGTTA
NM_012942.1	Cytochrome P450, family 7, subfamily a, polypeptide 1	Cyp7a1	CCTCCTGGCCTTCCTAAATC	AGACCTGGTCCCTCACACAC
NM_031241	Cytochrome P450, family 8, subfamily b, polypeptide 1	Cyp8b1	AGGGAATGCGGGCCAAGCAC	CCAGCATGGCCCGGTTGAGG
NW_047693	Fatty acid-Binding Protein	FACBP	ATGTCACCCAGTGTCATGGT	GAGGAGTGCGAACTGGAGAC
NM_031344	Fatty acid desaturase 2	Fads2	ATCTGCCCTACAACCACCAG	AGTTGAGGAAAACCAGGGCT
NM_017332	Fatty acid Synthase	FAS	TCGAGACACATCGTTTGAGC	TCAAAAAGTGCATCCAGCAG
NM_031561	Thrombospondin receptor	FAT/CD36	TCTCTCCTCGGATGGCTAGCTGA	TGGCCTGGTTCAACTAATTCATGCA
NM_130752.1	Fibroblast Growth Factor 21	FGF21	CTTCCTGCTGGGGGTGTGCG	CCAGGAGACTTTCTGGACTGCGG
NM_012597.2	Hepatic triacylglycerol lipase precursor (Hepatic lipase)	HL	CCACGGGTGGTCGGTGGATG	CTCCTGGCCCACAACACGGG
NM_013134.2	3-Hydroxy-3-methylglutaryl-CoA reductase	HMGCR	TGTGGCCAGGATGCAGCACAG	TGGTCCCACCACCCACGGTT
NM_017268.1	3-Hydroxy-3-methylglutaryl-CoA synthase 1	HMGCS	CTATGGGGGCACAGCCGCAG	TGTCCCACGAAGCCCTCGGT
NM_178866	Insulin-Like Growth Factor 1	IGF-1	GTTCCGATGTTTTGCAGGTT	TCTGAGGAGGCTGGAGATGT
NM_012588	Insulin-like growth factor binding protein 3	IGFBP-3	TACCAGGGTCTCCAACAAGG	GGCCCAGCAGAAATATCAAA
NM_022392.1	Insulin induced gene 1	INSIG1	ATATCCGTTCTTGGCTCCCT	TCCTTGCCTCCACTTGCTAT
NM_012592	Isovaleryl coenzyme A dehydrogenase	Ivd	TGGACCACACCATTCCCTAT	CCATCCAGGGCTACTTGTGT
NM_175762	Low density lipoprotein receptor	LDLR	TTCTTCAGGTTGGGGATCAG	CAGCTCTGTGTGAACCTGGA
NM_012738	Fatty acid-binding protein, liver 1	L-FAB	TGAAGGCGATGGGTCTGCCTGA	CGCACTCCTCCCCCAAGGTGA
NM_198780	Phosphoenolpyruvate carboxykinase 1	Pck1	GGAATTCTGGGAGAAGGAGG	CCCTAGCCTGTTCTCTGTGC
NM_013196.1	Peroxisome proliferator activated receptor alpha	PPAR α	TCACACAATGCAATCCGTTT	ACAAGCGTCTTCTCAGCCAT
NM_017101	Peptidylprolyl isomerase A, Ciclophilin A.	Ppia	GGTGACTTCACACGCCATAA	AGCCACTCAGTCTTGGCAGT
NM_058208	Suppressor of cytokine signaling 2	SOCS-2	AATGCTGAGTCGGCAGAAGT	GACGGGAAATTCAGATTGGA
NM_053565	Suppressor of cytokine signaling 3	SOCS-3	CGTTGACAGTCTTCCGACAA	CCTTTGAGGTTCAGGAGCAG
NM_001109274	Suppressor of cytokine signaling 5	SOCS-5	CGCAGTAGGCTCTCGCTCTTCTAA	CCTCGCCCAGACTGACGCTT
