# Supplementary material for: Lipid Profiling and Transcriptomic Analysis Reveals a Functional Interplay between Estradiol and Growth Hormone in Liver
Source: PLoS One. 2014 May 9;9(5):e96305. doi: 10.1371/journal.pone.0096305 (PMC4015979; doi:10.1371/journal.pone.0096305)
Supplement: Table S2 — Hepatic genes regulated by E2 in hypothyroid-orchidectomized rats. The administrations of vehicle (TXOX) or E2 (TXOXE2) to TXOX rats were described in Material and Methods. Then, differently expressed genes in the livers were identified by DNA microarrays. The analysis is based on the SAM statistical technique and differentially-expressed genes were discovered using a FDR less than 5% and a mean ratio of log2>|0.58|. The table shows ENSEMBL gene ID, Unigene/Refseq, gene symbol, gene description, R (TXOXE2/TXOX), SD, and q (%). (PDF) [file pone.0096305.s002.pdf]

ENSEMBL	Unigene/Refseq	Symbol	Gene description	R		q(%)
				mean ± SD		
Up-regulated genes						
ENSRNOG00000021354	NM_017158.2	Cyp2c7	cytochrome P450, family 2, subfamily c, polypeptide 7	3,45	0,59	0,00
ENSRNOG00000013093	NM_017158.2	Cyp2c7	cytochrome P450, family 2, subfamily c, polypeptide 7	3,43	0,35	0,00
ENSRNOG00000010410	NM_012657.2	Serpina3k	serine (or cysteine) peptidase inhibitor, clade A, member 3K	3,16	0,96	0,00
ENSRNOG00000028734	NM_017158	Cyp2c7	cytochrome P450, family 2, subfamily c, polypeptide 7	3,08	0,60	0,00
ENSRNOG00000021310	XM_220441	Slc36a3	solute carrier family 36 (proton/amino acid symporter), member 3	3,06	0,48	0,00
ENSRNOG00000004692	NM_022258	A1bg	alpha-1-B glycoprotein	2,97	0,46	0,00
ENSRNOG00000021336	XM_230313	XM_230313	tetratricopeptide repeat domain 17 (predicted)	2,75	0,33	0,00
ENSRNOG00000021380	XM_221105	Fads6	fatty acid desaturase domain family, member 6 (predicted)	2,75	0,38	0,00
ENSRNOG00000011696	NM_031048	Lifr	leukemia inhibitory factor receptor alpha	2,61	0,85	0,00
ENSRNOG00000027024	NM_001077589	RGS16	regulator of G-protein signaling 16	2,43	0,59	0,00
ENSRNOG00000014116	NM_133616	Sult2a1	Alcohol sulfotransferase (hyroxysteroid sulfotransferase)	2,35	0,91	0,00
ENSRNOG00000011708	NM_031572	Cyp2c12	Cytochrome P450 2C12, female specific (CYPIIC12)(15-BETA)	2,18	1,07	0,00
ENSRNOG00000010224	NM_001015012	RAB30	RAB30, member RAS oncogene family	1,97	0,43	0,00
ENSRNOG00000017727	NM_012630	PRLR	prolactin receptor	1,81	0,54	0,00
ENSRNOG00000004294	NM_022384	Ascl1	achaete-scute complex homolog 1 (Drosophila)	1,79	0,55	0,00
ENSRNOG00000001442	NM_031576	Por	P450 (cytochrome) oxidoreductase	1,71	0,52	0,00
ENSRNOG00000003020	NM_001001509	Hdmcp	mitochondrial hepatocellular carcinoma-downregulated carrier protein	1,70	0,57	0,00
ENSRNOG00000010478	NM_182474	SPI	Serine protease inhibitor	1,60	0,97	0,00
ENSRNOG00000007545	NM_199115	Angptl4	angiopoietin-like 4	1,53	0,41	0,00
ENSRNOG00000010635	NM_001004274	Igfbp4	insulin-like growth factor binding protein 4	1,50	0,20	0,00
ENSRNOG00000014365	NM_053765	Gne	glucosamine (UDP-N-acetyl)-2-epimerase/N-acetylmannosamine kinase	1,47	0,26	0,00
ENSRNOG00000019568	NM_138875	Jund	Jun D proto-oncogene	1,37	0,61	0,00
ENSRNOG00000027784	NM_001009965	Eiih	tsukushin; similar to hepatic protein EIIH	1,33	0,23	0,00
ENSRNOG00000026293	NM_021835	Jun	Jun oncogene	1,30	1,10	0,38
ENSRNOG00000018450	XM_347185	Slc25a22	solute carrier family 25 (mitochondrial carrier, glutamate), member 22	1,30	0,47	0,00
ENSRNOG00000010198	XM_234398	LACH2	cytosolic acyl CoA thioester hydrolase, inducible (long chain acyl-CoA hydrolase) (CTE-I) (LACH2)	1,29	0,52	0,00
ENSRNOG00000005661	NM_024351	nd	heat shock cognate 71 KDA protein	1,29	0,20	0,00
ENSRNOG00000020266	NM_017245	LOC305181	similar to Elongation factor 2 (EF-2); eukaryotic translation elongation factor 2	1,24	0,16	0,00
ENSRNOG00000018145	NM_001004085	CrAT	carnitine O-acetyltransferase (mitochondrial protein)	1,21	0,60	0,00
ENSRNOG00000020217	NM_001013901	PA1	similar to RIKEN cDNA 2900092E17	1,20	0,64	0,00
ENSRNOG00000019834	NM_001004082	hsp90-beta	similar to heat shock protein 1, beta; similar to HSP 90-beta (HSP 84); HSP 90kDa alpha (cytosolic), class B member	1,18	0,22	0,00
ENSRNOG00000017601	NM_017070	Srd5a1	steroid-5-alpha-reductase, alpha polypeptide 1 (3-oxo-5 alpha-steroid delta 4-dehydrogenase alpha 1)	1,14	0,43	0,00
ENSRNOG00000017749	NM_133540	Nkg7	natural killer cell group 7 sequence	1,13	0,76	0,19
ENSRNOG00000013788	XM_003754118.1	LOC100360180	6-phosphogluconate dehydrogenase, decarboxylating-like (LOC100360180),	1,13	0,47	0,00
ENSRNOG00000005341	XM_229938	Upp2	uridine phosphorylase 2	1,13	0,35	0,00
ENSRNOG00000020194	NM_001013179	Hes6	hairy and enhancer of split 6 (Drosophila)	1,12	0,72	0,11
ENSRNOG00000019312	NM_001013070	Tspan4	tetraspanin 4	1,09	0,35	0,00
ENSRNOG00000024496	NM_001009717	NM_001009717	similar to leucine-rich alpha-2-glycoprotein	1,09	0,19	0,00
ENSRNOG00000016170	XM_215293	XM_215293	vanin 1 (predicted)	1,08	0,37	0,00
ENSRNOG00000022157	XM_225266	RGD1561697	similar to NK13	1,08	0,96	0,51
ENSRNOG00000012605	NM_053976.1	Krt18	keratin 18	1,07	0,30	0,00
ENSRNOG00000022138	NM_024351	nd	heat shock cognate 71 KDA protein	1,06	0,33	0,00
ENSRNOG00000022699	XM_220739	LOC303341	hypothetical LOC303341	1,06	0,28	0,00
ENSRNOG00000027967	NM_001079709.1	Acot5	acyl-CoA thioesterase 5	1,03	0,38	0,00
ENSRNOG00000014914	NM_001008527	nd	aldehyde oxidase 3	1,03	0,41	0,00
ENSRNOG00000015483	NM_019340	nd	regulator of G-protein signaling 3 (RGS3) (fragment)	1,03	0,37	0,00
ENSRNOG00000014338	NM_145677	Slc25a25	solute carrier family 25 (mitochondrial carrier, phosphate carrier), member 25	1,00	1,08	0,80
ENSRNOG00000020480	NM_053445	Fads1	fatty acid desaturase 1	1,00	0,31	0,00
ENSRNOG00000020067	NM_182675	Rab40c	Rab40c, member RAS oncogene family	1,00	0,75	0,38
ENSRNOG00000019538	NM_001000194	NM_001000194	olfactory receptor gene Olr208	0,99	0,94	0,63
ENSRNOG00000009434	XM_236574	Aa2-001	similar to RIKEN cDNA 1300017J02	0,98	0,18	0,00
ENSRNOG00000021364	Rn.131314	LOC691311	hypothetical protein LOC691311; hypothetical protein LOC687852	0,97	0,46	0,08
ENSRNOG00000028347	NM_183403	Gpx2	glutathione peroxidase 2	0,97	0,43	0,00
ENSRNOG00000022180	XM_002725097.2	LOC100364618	hypothetical protein LOC100364618	0,96	0,74	0,45
ENSRNOG00000008215	XM_221124	LOC690374	tripartite motif-containing 47	0,96	0,27	0,00
ENSRNOG00000017914	NM_134449	Prkcdbp	protein kinase C, delta binding protein	0,95	0,49	0,11
ENSRNOG00000013552	NM_139192.2	Scd1	acyl-CoA desaturase 1 (stearoyl-Co A desaturase 1) (Fatty acid desaturase 1) (Delta(9)-desaturase 1)	0,95	1,59	2,99
ENSRNOG00000027116	XR_146223.1	LOC100362689	keratin 8-like (LOC100362689)	0,94	0,24	0,00
ENSRNOG00000018736	NM_001108509.2	Pnpla2	patatin-like phospholipase domain containing 2	0,94	0,14	0,00
ENSRNOG00000024713	XM_003749431.1	LOC100911966	ribosomal protein L7a-like	0,93	0,23	0,00
ENSRNOG00000022075	NM_001114391.1	Rpl7a	ribosomal protein L7a	0,93	0,25	0,00
ENSRNOG00000023633	XM_236253	Crabp1	cellular retinoic acid binding protein 1	0,93	0,05	0,00
ENSRNOG00000024042	XM_215513	XM_215513	similar to cytokeratin	0,92	0,21	0,00
ENSRNOG00000016097	Rn.110564	Ccbl1	cysteine conjugate-beta lyase, cytoplasmic	0,92	0,24	0,00
ENSRNOG00000019630	NM_053493	HACL1	2-hydroxyacyl-CoA lyase 1	0,91	0,50	0,09
ENSRNOG00000022721	XM_003751878.1	RGD1559899	PREDICTED: Rattus norvegicus C-type lectin domain family 18 member A-like (RGD1559899)	0,90	0,29	0,00
ENSRNOG00000005906	NM_031561	FAT/CD36	fatty acid translocase/CD36 (thrombospondin receptor)	0,90	0,48	0,11
ENSRNOG00000012622	XM_238034	Mmp15	matrix metallopeptidase 15	0,89	0,39	0,08
ENSRNOG00000022047	XM_224924.5	RGD1561928	60S ribosomal protein L9-like (RGD1561928)	0,89	0,03	0,00
ENSRNOG00000018649	XM_230856	Slc35C2	solute carrier family 35, member C2	0,88	0,43	0,11
ENSRNOG00000019638	NM_001002016.1	Lmna	lamin A (Lmna), transcript variant 2	0,88	0,24	0,00
ENSRNOG00000028377	XM_345572	Mpl	myeloproliferative leukemia virus oncogene	0,88	0,13	0,00
ENSRNOG00000016827	NM_145776	Slc38a3	solute carrier family 38, member 3	0,87	0,46	0,11
ENSRNOG00000020308	NM_022594	Ech1	enoyl coenzyme A hydratase 1, peroxisomal	0,87	0,39	0,08
ENSRNOG00000017093	XM_341518	MGC116297	similar to 1300014I06Rik protein	0,87	0,23	0,04
ENSRNOG00000023638	NM_031769.1	Pdxk	pyridoxal (pyridoxine, vitamin B6) kinase (Pdxk)	0,87	0,19	0,00
ENSRNOG00000027674	NM_031579.2	Ptp4a1	protein tyrosine phosphatase type IVA, member 1	0,86	0,27	0,00
ENSRNOG00000028139	XM_346272	XM_346272	similar to ferritin heavy chain - chicken	0,86	0,63	0,45
ENSRNOG00000013251	NM_001044266.1	Trim24	transcription intermediary factor 1-alpha	0,86	0,27	0,00
ENSRNOG00000019180	NM_053623	ACSL4	acyl-CoA synthetase long-chain family member 4	0,85	0,42	0,11
ENSRNOG00000018567	Rn.162702	Slc20a1	solute carrier family 20 (phosphate transporter), member 1	0,85	0,52	0,26

ENSRNOG00000025850	XM_001072134.2	LOC684838	60S ribosomal protein L7a-like	0,85	0,12	0,00
ENSRNOG00000019283	NM_017255	P2RY2	purinergic receptor P2Y, G-protein coupled 2	0,84	0,41	0,11
ENSRNOG00000022879	NM_001033684.1	Cct6a	chaperonin containing Tcp1, subunit 6A (zeta 1) (Cct6a)	0,84	0,30	0,00
ENSRNOG00000009597	NM_175837	Cyp4a1	cytochrome P450 4A1	0,84	0,70	0,51
ENSRNOG00000021115	XM_219534	nd	similar to 2610524G07Rik protein	0,84	0,33	0,00
ENSRNOG00000028037	XM_343758	XM_343758	similar to protein tyrosine phosphatase 4a1	0,84	0,32	0,00
ENSRNOG00000019626	NM_024143	Slc27a5	solute carrier family 27 (fatty acid transporter), member 5	0,84	0,41	0,11
ENSRNOG00000020029	XM_340774	LOC682248	hypothetical protein LOC682248; similar to RIKEN cDNA 9530058B02	0,83	0,21	0,00
ENSRNOG00000016030	XM_001059941.3	RGD1559149	similar to 60S ribosomal protein L7a	0,83	0,23	0,00
ENSRNOG00000019484	NM_053818	Slc6a9	solute carrier family 6 (neurotransmitter transporter, glycine), member 9	0,83	0,18	0,00
ENSRNOG00000025879	XM_341252	nd	similar to ribosomal protein L13	0,83	0,21	0,00
ENSRNOG00000006612	XM_239239	Map2k3	mitogen activated protein kinase kinase 3	0,83	0,39	0,26
ENSRNOG00000019858	NM_001009690	Rom1	retinal outer segment membrane protein 1	0,82	0,27	0,00
ENSRNOG00000016131	NM_133602	nd	MAP2 RNA trans-acting protein marta 1	0,81	0,33	0,08
ENSRNOG00000026871	XR_147054.1	LOC503131	PREDICTED: Rattus norvegicus glyceraldehyde-3-phosphate dehydrogenase-like (LOC503131)	0,81	0,43	0,19
ENSRNOG00000020989	NM_001013071	Tm7sf2	transmembrane 7 superfamily member 2	0,81	0,34	0,08
ENSRNOG00000020144	XM_341935	RGD1310127	similar to cDNA sequence BC017158	0,81	0,44	0,19
ENSRNOG00000021246	XM_342521	CENPB	centromere protein B	0,81	0,25	0,00
ENSRNOG00000023459	NM_001109263.1	Lurap1	leucine rich adaptor protein 1 (Lurap1)	0,81	0,66	0,51
ENSRNOG00000017962	NM_199085	Serpinb6a	serine (or cysteine) peptidase inhibitor, clade B, member 6a	0,81	0,28	0,00
ENSRNOG00000028121	XM_221107	OTOP2	otopetrin 2 (predicted)	0,81	0,10	0,00
ENSRNOG00000016957	NM_013122	Igfbp2	insulin-like growth factor binding protein 2	0,80	0,61	0,51
ENSRNOG00000014791	XM_218226	PEG3	paternally expressed 3	0,79	0,30	0,08
ENSRNOG00000017243	NM_053420	BNIP3	BCL2/adenovirus E1B 19 kDa-interacting protein 3	0,79	0,31	0,08
ENSRNOG00000020293	NM_022639	Chrna10	cholinergic receptor, nicotinic, alpha 10	0,79	0,09	0,00
ENSRNOG00000015502	XM_215693	Hiat1	hippocampus abundant gene transcript 1	0,79	0,37	0,11
ENSRNOG00000018666	NM_144745	AGS3	G-protein signaling modulator 1 (AGS3-like, C. elegans)	0,79	0,32	0,11
ENSRNOG00000026899	XM_001076433.2	RGD1561520	PREDICTED: Rattus norvegicus ferritin light chain 1-like (RGD1561520)	0,78	0,38	0,11
ENSRNOG00000022514	NM_001007744	Tor2a	torsin family 2, member A	0,78	0,26	0,00
ENSRNOG00000016520	XM_236582	XM_236582	similar to 5730439E10Rik protein (predicted)	0,78	0,39	0,14
ENSRNOG00000011635	XM_341636	Ces5	carboxylesterase 5	0,78	0,72	1,32
ENSRNOG00000008190	Rn.162643	Ntel1	patatin-like phospholipase domain containing 7	0,78	0,11	0,00
ENSRNOG00000021102	NM_017288	SCN1B	sodium channel, voltage-gated, type I, beta	0,78	0,34	0,11
ENSRNOG00000021393	XM_233976	XM_233976	similar to B(0,)-type amino acid transporter 1 (B(0,)AT) (Glycoprotein-associated amino acid transporter b0,L')	0,78	0,27	0,00
ENSRNOG00000001259	XM_222184	CUX2	similar to Homeobox protein cut-like 2 (Homeobox protein Cux-2) (Cut-like 2)	0,77	0,29	0,08
ENSRNOG00000020704	XM_342023	DAK	dihydroxyacetone kinase 2 homolog (S. cerevisiae)	0,77	0,46	0,38
ENSRNOG00000021027	NM_012543	Dbp	D site of albumin promoter (albumin D-box) binding protein	0,77	0,41	0,19
ENSRNOG00000003758	XM_215591	LOC363224	similar to heat shock protein 1, beta; similar to HSP 90-beta (HSP 84) (Tumor-specific transplantation 84 kDa antigen	0,77	0,13	0,00
ENSRNOG00000021008	XM_225971	KCTD16	similar to potassium channel tetramerisation domain containing 16	0,77	0,35	0,11
ENSRNOG00000020345	XM_224736	LOC689414	solute carrier family 25, member 42	0,77	0,08	0,00
ENSRNOG00000005642	XM_235164	frs2	fibroblast growth factor receptor substrate 2	0,76	0,64	0,63
ENSRNOG00000020420	NM_012624	pk1	pyruvate kinase, liver and RBC	0,76	0,62	0,63
ENSRNOG00000024572	NM_001000478	NM_001000478	olfactory receptor gene Olr1334	0,76	0,27	0,08
ENSRNOG00000007395	NM_017300	BAAT	bile acid Co A: amino acid N-acyltransferase (glycine N-choloyltransferase)	0,76	0,14	0,00
ENSRNOG00000011526	NM_012999	Pace4	proprotein convertase subtilisin/kexin type 6	0,76	0,17	0,00
ENSRNOG00000021370	Rn.163094	Ccdc69	coiled-coil domain containing 69	0,76	0,22	0,00
ENSRNOG00000025567	NM_178103.2	Eddm3b	epididymal secretory protein 3	0,75	0,57	0,51
ENSRNOG00000014008	XM_216525	MFSD2	major facilitator superfamily domain containing 2	0,75	0,76	0,87
ENSRNOG00000020238	XM_340909	Plekhh3	pleckstrin homology domain containing, family H (with MyTH4 domain) member 3	0,75	0,13	0,00
ENSRNOG00000020177	NM_001191951.1	Ahnak	AHNAK nucleoprotein (Ahnak)	0,75	0,39	0,26
ENSRNOG00000021463	NM_013196	Ppara	peroxisome proliferator activated receptor alpha	0,75	0,27	0,08
ENSRNOG00000026958	NM_001024325.1	LOC500077	similar to RIKEN cDNA 3110062M04 (LOC500077)	0,75	0,57	0,51
ENSRNOG00000027833	XM_227287	RGD1562174	similar to mKIAA1450 protein	0,75	0,58	0,51
ENSRNOG00000018413	NM_023978.2	Per3	period circadian clock 3 (Per3)	0,75	0,63	0,63
ENSRNOG00000016410	NM_001009636	Preli	PRELI domain containing 1; similar to 2610524G07Rik protein	0,75	0,37	0,19
ENSRNOG00000021750	NM_012797	Id1	inhibitor of DNA binding 1	0,74	0,21	0,00
ENSRNOG00000026094	NM_001099495.1	Vom2r54	vomeronasal 2 receptor, 55 (Vom2r55)	0,74	0,46	0,38
ENSRNOG00000012626	XM_234443	nd	similar to Expressed sequence AI324246	0,74	0,32	0,11
ENSRNOG00000018944	XM_215283	Pank1	pantothenate kinase 1	0,74	0,18	0,00
ENSRNOG00000015822	NM_001135094.1	Klf14	Kruppel-like factor 14 (Klf14)	0,74	0,56	0,51
ENSRNOG00000022166	XM_002730211.2	Ammecr1	AMME syndrome candidate gene 1 protein homolog, transcript variant 1 (RGD1561004),	0,73	0,17	0,00
ENSRNOG00000019383	NM_019194	Tef	thyrotrophic embryonic factor	0,73	0,38	0,26
ENSRNOG00000005615	NM_024127	Gadd45a	growth arrest and DNA-damage-inducible, alpha	0,73	0,32	0,11
ENSRNOG00000023719	XM_340769	XM_340769	similar to hypothetical protein FLJ12681 (predicted)	0,73	0,24	0,08
ENSRNOG00000027142	ENSRNOG00000027142	RGD1565680	similar to Hypothetical protein DJ1198H6.2	0,72	0,17	0,00
ENSRNOG00000008755	NM_017340	ACOX1	acyl-CoA oxidase 1, palmitoyl	0,72	0,49	0,45
ENSRNOG00000020591	NM_021598	nd	mast cell protease VIII precursor (RMCP-VIII) (RMCP-8)	0,72	0,51	0,51
ENSRNOG00000018132	XM_346958	LOC361335	LOC361334	0,72	0,53	0,51
ENSRNOG00000008167	NM_001007680	Abhd6	abhydrolase domain containing 6	0,72	0,33	0,19
ENSRNOG00000017683	XM_341456	Adam18	ADAM metallopeptidase domain 18	0,72	0,32	0,19
ENSRNOG00000023879	XM_233225	XM_233225	similar to 40S RIBOSOMAL PROTEIN SA (P40) (34/67 KD LAMININ RECEPTOR)	0,72	0,13	0,00
ENSRNOG00000017210	XM_341519	Nritp	solute carrier family 22, member 23	0,72	0,25	0,08
ENSRNOG00000011281	XM_003749251.1	EIF4G1	eukaryotic translation initiation factor 4 gamma 1-like (LOC100911693)	0,71	0,13	0,00
ENSRNOG00000002642	XM_343135	PTGES3	Prostaglandin E synthase 3	0,71	0,09	0,00
ENSRNOG00000017997	XM_224610	NEK4	NIMA (never in mitosis gene a)-related expressed kinase 4 (predicted)	0,71	0,50	0,51
ENSRNOG00000023159	XM_221123	nd	40S ribosomal protein S18 (KE-3)	0,71	0,17	0,00
ENSRNOG00000008116	NM_138905	Ppap2b	phosphatidic acid phosphatase type 2B	0,71	0,48	0,51
ENSRNOG00000023308	XM_217585	nd	ferritin light chain (ferritin L subunit).	0,71	0,36	0,26
ENSRNOG00000007141	XM_216656	Preb	Prolactin regulatory element-binding protein	0,70	0,17	0,00
ENSRNOG00000028484	XM_228162	Rshl3	radial spokehead-like 3	0,70	0,24	0,08
ENSRNOG00000018259	XM_217346	XM_217346	serum response factor (predicted)	0,70	0,45	0,45
ENSRNOG00000022364	NM_199490	Dsg4	desmoglein 4	0,70	0,31	0,19
ENSRNOG00000021139	NM_001008511	Esrra	estrogen related receptor, alpha	0,70	0,08	0,00
ENSRNOG00000021375	XM_344413	XM_344413	similar to putative E1-E2 ATPase	0,70	0,20	0,00

ENSRNOG00000011289	XM_215538	nd	similar to eukaryotic translation initiation factor 4G I	0,70	0,41	0,38
ENSRNOG00000025114	NM_024125	Cebpb	CCAAT/enhancer binding protein (C/EBP), beta	0,70	0,60	0,71
ENSRNOG00000008095	NM_022671	Onecut1	one cut homeobox 1 (HNF-6)	0,70	0,39	0,38
ENSRNOG00000020990	NM_130752	FGF21	fibroblast growth factor 21	0,69	0,57	0,63
ENSRNOG00000010326	NM_145090	Arf GAP1	ADP-ribosylation factor GTPase activating protein 1; collagen, type XX, alpha 1	0,69	0,33	0,26
ENSRNOG00000019936	XM_215003	RGD1311634	similar to RIKEN cDNA 3200002M19	0,69	0,45	0,51
ENSRNOG00000020321	XM_214175	RGD1308396	similar to chromosome 14 open reading frame 21	0,69	0,29	0,19
ENSRNOG00000023660	XM_342702	nd	similar to Fam13a1 protein (predicted)	0,69	0,13	0,00
ENSRNOG00000012683	XM_341879	FAM108C1	family with sequence similarity 108, member C1	0,69	0,27	0,11
ENSRNOG00000008109	NM_022694	nd	P105 coactivator	0,69	0,24	0,11
ENSRNOG00000019428	NM_080902.4	Higd1a	HIG1 hypoxia inducible domain family, member 1A (Higd1a)	0,69	0,17	0,00
ENSRNOG00000011330	NM_144743	Ces6	carboxylesterase 6	0,69	0,68	0,87
ENSRNOG00000027773	XM_234484	SMEK1	SMEK homolog 1, suppressor of mek1 (Dictyostelium)	0,68	0,47	0,51
ENSRNOG00000003872	NM_133315	Slc40a1	solute carrier family 39 (iron-regulated transporter), member 1	0,68	0,19	0,10
ENSRNOG00000015701	XM_225250	Rreb1	ras responsive element binding protein 1	0,68	0,19	0,08
ENSRNOG00000001496	XM_341791	nd	similar to Alcohol sulfotransferase (Hydroxysteroid sulfotransferase) (ST) (ST-60)	0,68	0,42	0,45
ENSRNOG00000023268	XM_227276	XM_227276	similar to Nucleolar GTP-binding protein 1 (Chronic renal failure gene protein) (GTP-binding protein NGB)	0,68	0,24	0,11
ENSRNOG00000015654	NM_017094.1	Ghr	growth hormone receptor	0,68	0,24	0,11
ENSRNOG00000026919	NM_001270038.1	RGD1565367	similar to Solute carrier family 23, member 2 (Sodium-dependent vitamin C transporter 2) (RGD1565367)	0,67	0,24	0,11
ENSRNOG00000027329	XM_345625	Dhx57	DEAH (Asp-Glu-Ala-Asp/His) box polypeptide 57	0,67	0,47	0,51
ENSRNOG00000021317	NM_001005905	Cct2	chaperonin containing TCP1, subunit 2 (beta)	0,67	0,07	0,00
ENSRNOG00000023208	XM_345524	RGD1309821	similar to KIAA1161 protein	0,67	0,17	0,00
ENSRNOG00000028553	NM_001001361.1	Olr903	olfactory receptor 903 (Olr903)	0,67	0,14	0,00
ENSRNOG00000015195	XM_216417	HDHD3	haloacid dehalogenase-like hydrolase domain containing 3	0,67	0,21	0,08
ENSRNOG00000011414	NM_031595	Psmc3	proteasome (prosome, macropain) 26S subunit, ATPase 3	0,67	0,28	0,19
ENSRNOG00000023332	XM_227369	nd	similar to hornerin (predicted)	0,67	0,28	0,19
ENSRNOG00000025613	NM_022693	nd	SH3-domain binding protein 4	0,67	0,20	0,08
ENSRNOG00000025923	XM_214087	ZMIZ2	zinc finger, MIZ-type containing 2	0,67	0,35	0,38
ENSRNOG00000028530	XM_344783	ZFPM1	zinc finger protein, multitype 1	0,67	0,22	0,11
ENSRNOG00000014742	XM_001080794.3	Ankrd13b	PREDICTED: Rattus norvegicus ankyrin repeat domain 13B (Ankrd13b)	0,67	0,53	0,63
ENSRNOG00000025052	XM_218848	STARD5	StAR-related lipid transfer (START) domain containing 5	0,67	0,43	0,51
ENSRNOG00000024752	NM_172222	NM_172222	complement component 2	0,67	0,35	0,38
ENSRNOG00000010931	XM_342338	nd	similar to hypothetical protein PRO0971 (predicted)	0,67	0,31	0,26
ENSRNOG00000018621	XM_214812	Leng9	leukocyte receptor cluster (LRC) member 9	0,67	0,50	0,63
ENSRNOG00000017276	NM_001001000	Olr167	olfactory receptor 167; olfactory receptor 162	0,67	0,53	0,71
ENSRNOG00000007758	XM_345770	Hsd17b6	hydroxysteroid (17-beta) dehydrogenase 6	0,66	0,28	0,19
ENSRNOG00000028191	NM_001014162.4	ACSm5	acyl-CoA synthetase medium-chain family member 5 (Acsm5)	0,66	0,65	1,22
ENSRNOG00000016818	NM_053429	Fgfr3	fibroblast growth factor receptor 3	0,66	0,23	0,11
ENSRNOG00000010222	NM_012570	GLUD1	glutamate dehydrogenase 1	0,66	0,26	0,19
ENSRNOG00000010543	NM_001034150.1	Srpr	signal recognition particle receptor ('docking protein')	0,66	0,16	0,00
ENSRNOG00000026638	NM_031726.1	scamp5	secretory carrier membrane protein 5 (Scamp5)	0,66	0,35	0,38
ENSRNOG00000023639	XM_213253	Rpusd1	RNA pseudouridylate synthase domain containing 1	0,66	0,13	0,00
ENSRNOG00000027124	NM_053729	Tdg	thymine-DNA glycosylase	0,66	0,23	0,11
ENSRNOG00000026722	NM_001025131.1	Sult2a2	sulfotransferase family 2A, dehydroepiandrosterone (DHEA)-preferring, member 2 (Sult2a2)	0,66	0,49	0,63
ENSRNOG00000020315	XM_227586	Taf13	TAF13 RNA polymerase II, TATA box binding protein (TBP)-associated factor	0,66	0,17	0,08
ENSRNOG00000010958	NM_022540	Prdx3	peroxiredoxin 3	0,66	0,49	0,63
ENSRNOG00000028447	NM_001000361	NM_001000361	olfactory receptor gene Olr718	0,66	0,24	0,11
ENSRNOG00000011305	NM_019193	Sox10	SRY (sex determining region Y)-box 10	0,66	0,61	0,87
ENSRNOG00000021033	NM_001012001	NM_001012001	leucine rich repeat containing 2 (predicted)	0,65	0,47	0,63
ENSRNOG00000011205	NM_013025	Ccl3	chemokine (C-C motif) ligand 3	0,65	0,26	0,19
ENSRNOG00000015026	NM_023982	Arhgef11	Rho guanine nucleotide exchange factor (GEF) 11	0,65	0,40	0,45
ENSRNOG00000016573	NM_001012345	Dgat2	diacylglycerol O-acyltransferase homolog 2 (mouse)	0,65	0,38	0,45
ENSRNOG00000015496	NM_012678	TPM4	tropomyosin 4	0,65	0,32	0,38
ENSRNOG00000010067	NM_031342	Lypla2	lysophospholipase 2; Acyl-protein thioesterase 2	0,65	0,31	0,26
ENSRNOG00000025627	XM_235384	XM_235384	Cohen syndrome 1 (predicted)	0,65	0,19	0,08
ENSRNOG00000020876	Rn.10668	Bax	similar to BAX protein, cytoplasmic isoform delta; Bcl2-associated X protein	0,65	0,23	0,11
ENSRNOG00000004448	NM_001108091.1	Acss3	acyl-CoA synthetase short-chain family member 3, mitochondrial	0,65	0,08	0,00
ENSRNOG00000027339	XM_342164	nd	similar to Heph protein	0,65	0,19	0,08
ENSRNOG00000023348	XM_232990	TBC1D2	TBC1 domain family, member 2	0,65	0,20	0,11
ENSRNOG00000001009	NM_001009604	BRI3	brain protein I3	0,65	0,31	0,26
ENSRNOG00000008553	XM_342975	Mthfr	5,10-methylenetetrahydrofolate reductase (NADPH)	0,65	0,49	0,63
ENSRNOG00000016924	NM_016987	ACLY	ATP citrate lyase	0,65	1,07	3,73
ENSRNOG00000008946	NM_024139	RGD1565588	similar to calcium binding protein P22	0,65	0,28	0,19
ENSRNOG00000005479	NM_017215	Slc1a2	solute carrier family 1 (glial high affinity glutamate transporter), member 2	0,65	0,32	0,38
ENSRNOG00000010918	NM_012524	Cebpa	CCAAT/enhancer binding protein (C/EBP), alpha	0,65	0,73	1,71
ENSRNOG00000021154	NM_053549	nd	Vascular endothelial growth factor B (VEGF-B)	0,65	0,31	0,38
ENSRNOG00000009447	Rn.161839	LOC500118	similar to RIKEN cDNA D330028D13	0,65	0,23	0,11
ENSRNOG00000020930	XM_213493	RGD1311493	similar to CG13379-PA	0,64	0,20	0,11
ENSRNOG00000021330	XM_226564	RGD1559496	Gm505	0,64	0,30	0,26
ENSRNOG00000019634	NM_001000824.1	Olr421	olfactory receptor 421 (Olr421)	0,64	0,32	0,38
ENSRNOG00000012073	NM_001007664	Abhd14b	abhydrolase domain containing 14b	0,64	0,33	0,38
ENSRNOG00000019798	NM_001025625	STUB1	STIP1 homology and U-Box containing protein 1	0,64	0,28	0,26
ENSRNOG00000024385	XM_217111	BARS-38	Glyceraldehide 3-phosphate dehydrogenase (38 KDA BFA-dependent ADP-robosylation substrate) (BARS-38)	0,64	0,35	0,45
ENSRNOG00000017437	XM_003751278.1	LOC100359583	PREDICTED: Rattus norvegicus hypothetical protein LOC100359583	0,64	0,06	0,00
ENSRNOG00000019393	XM_217039	eplin	LIM domain and actin binding 1	0,64	0,27	0,26
ENSRNOG00000021306	NM_001000342	NM_001000342	olfactory receptor gene Olr655	0,64	0,28	0,26
ENSRNOG00000016167	NM_001109133.1	Spata2L	spermatogenesis associated 2-like (Spata2L)	0,64	0,21	0,11
ENSRNOG00000027367	XM_225091	XM_225091	similar to testicular serine protease 1	0,64	0,11	0,00
ENSRNOG00000020229	NM_053310	homer3	homer homolog 3 (Drosophila)	0,64	0,20	0,11
ENSRNOG00000008855	NM_001004099	CXN-26	gap junction protein, beta 2	0,64	0,44	0,51
ENSRNOG00000025514	XM_001070873.3	Zfp335	PREDICTED: Rattus norvegicus zinc finger protein 335, transcript variant 2 (Zfp335)	0,64	0,39	0,51
ENSRNOG00000025620	NM_017142.1	Adcy8	adenylate cyclase 8 (brain) (Adcy8)	0,64	0,18	0,11
ENSRNOG00000022747	XM_003752284.1	LOC100362470	PREDICTED: Rattus norvegicus MKL/myocardin-like 2 (LOC100362470)	0,63	0,32	0,38
ENSRNOG00000010103	NM_001008324	EIF4B	eukaryotic translation initiation factor 4B	0,63	0,18	0,11

ENSRNOG00000009565	NM_053551	Pdk4	pyruvate dehydrogenase kinase, isozyme 4	0,63	0,66	1,22
ENSRNOG00000016218	NM_133541	Gtf3c1	general transcription factor III C 1	0,63	0,27	0,26
ENSRNOG00000024781	XM_237984	XM_237984	similar to low density lipoprotein receptor-related protein 2; glycoprotein 330; low density lipoprotein-related proteir	0,63	0,36	0,45
ENSRNOG00000024500	NM_017008.4	Gapdh	glyceraldehyde-3-phosphate dehydrogenase (Gapdh)	0,63	0,38	0,51
ENSRNOG00000021164	NM_138911	LOC691340	similar to stress-induced phosphoprotein 1	0,63	0,14	0,08
ENSRNOG00000026631	XM_001062428.2	LOC685125	PREDICTED: Rattus norvegicus disks large homolog 5-like (LOC685125)	0,63	0,49	0,71
ENSRNOG00000028404	XM_340891	Ppp1r1b	protein phosphatase 1, regulatory (inhibitor) subunit 1B	0,63	0,18	0,11
ENSRNOG00000014650	XM_342055	Sema4g	sema domain, immunoglobulin domain (Ig), transmembrane domain (TM) and short cytoplasmic domain, (semaphori	0,63	0,53	0,87
ENSRNOG00000028103	NM_001008281	Psmd3	proteasome (prosome, macropain) 26S subunit, non-ATPase, 3	0,63	0,17	0,11
ENSRNOG00000007133	XM_243652	Plxnb2	plexin B2	0,62	0,26	0,26
ENSRNOG00000001379	NM_147206	Cyp3a9	Cytochrome P450 3A9	0,62	0,49	0,71
ENSRNOG00000026605	NM_206846	Ifi27l2b	interferon, alpha-inducible protein 27 like 2B	0,62	0,47	0,71
ENSRNOG00000021083	NM_001000251	Olr329	olfactory receptor 329	0,62	0,19	0,11
ENSRNOG00000004101	NM_001100811.1	Marc1	mitochondrial amidoxime reducing component 1 (Marc1), nuclear gene encoding mitochondrial protein	0,62	0,45	0,63
ENSRNOG00000016150	XM_217254	Ifrd2	interferon-related developmental regulator 2	0,62	0,25	0,26
ENSRNOG00000028636	NM_001012228.1	Xkr5	XK, Kell blood group complex subunit-related family, member 5	0,62	0,35	0,45
ENSRNOG00000026025	Rn.123410	Capn11	calpain 11	0,62	0,10	0,00
ENSRNOG00000023679	XM_342704	Prdm5	PR domain containing 5 (predicted)	0,62	0,16	0,11
ENSRNOG00000016344	XM_342286	Slc39a1	solute carrier family 39 (zinc transporter), member 1	0,62	0,30	0,38
ENSRNOG00000022769	XM_343611	LOC363269	SP100 nuclear antigen	0,62	0,24	0,19
ENSRNOG00000008843	NM_017306	Dci	dodecenoyl-Co A delta isomerase (3,2 trans-enoyl-CoA isomerase)	0,62	0,36	0,45
ENSRNOG00000026000	ENSRNOG00000026000	Vom2r7	similar to putative pheromone receptor (Go-VN5); vomeronasal 2 receptor, 7	0,62	0,05	0,00
ENSRNOG00000022392	NM_053612	Hspb8	heat shock protein 8	0,62	0,24	0,26
ENSRNOG00000020206	NM_134334	Ctsd	cathepsin D	0,62	0,34	0,45
ENSRNOG00000025383	NM_001004072	LOC685778	pyruvate dehydrogenase E1 alpha 1 pseudogene; pyruvate dehydrogenase (lipoamide) alpha 1	0,62	0,25	0,26
ENSRNOG00000006619	XM_344286	Dnajc9	DnaJ (Hsp40) homolog, subfamily C, member 9	0,62	0,65	1,71
ENSRNOG00000012443	NM_012930	CPT-2	carnitine palmitoyltransferase 2	0,62	0,23	0,19
ENSRNOG00000020253	NM_001008370	RAB1B	similar to Ras-related protein Rab-1B	0,62	0,27	0,26
ENSRNOG00000028629	NM_033230	Akt1	v-akt murine thymoma viral oncogene homolog 1	0,61	0,30	0,38
ENSRNOG00000000465	NM_001008885	RT1-Ke4	solute carrier family 39 (zinc transporter), member 7	0,61	0,17	0,11
ENSRNOG00000020507	NM_001127654.1	Tm6sf2	transmembrane 6 superfamily member 2 (Tm6sf2)	0,61	0,27	0,38
ENSRNOG00000009028	XM_234880	Rnf126	ring finger protein 126	0,61	0,26	0,26
ENSRNOG00000023885	XM_344587	nd	similar to triosephosphate isomerase 1	0,61	0,27	0,38
ENSRNOG00000014187	NM_052807	Igf1r	insulin-like growth factor 1 receptor	0,61	0,45	0,63
ENSRNOG00000010216	XM_214237	RGD1308772	similar to KIAA0564 protein	0,61	0,23	0,19
ENSRNOG00000022993	XM_236595	XM_236595	similar to RIKEN cDNA E330026B02 (predicted)	0,61	0,38	0,51
ENSRNOG00000022031	XM_343232	XM_343232	similar to 60S ribosomal protein L7a (Surfeit locus protein 3) (PLA-X polypeptide)	0,61	0,15	0,08
ENSRNOG00000013089	NM_213626	kif13B	kinesin 13B	0,61	0,32	0,45
ENSRNOG00000020039	NM_022590	Slc5a2	solute carrier family 5 (sodium/glucose cotransporter), member 2	0,61	0,29	0,38
ENSRNOG00000011078	NM_053464	Srm	spermidine synthase	0,61	0,38	0,51
ENSRNOG00000027670	XM_216629	XM_216629	ADP-ribosylation factor-like 6 interacting protein 2 (predicted)	0,61	0,18	0,11
ENSRNOG00000025748	XM_222038	nd	similar to putative pheromone receptor	0,61	0,25	0,26
ENSRNOG00000004793	XM_234929	Ncln	nicalin homolog (zebrafish)	0,61	0,09	0,00
ENSRNOG00000017882	XM_344631	RGD1560691	Camk1d	0,61	0,20	0,11
ENSRNOG00000015591	NM_001010920	Cndp2	CNDP dipeptidase 2 (metallopeptidase M20 family)	0,61	0,27	0,38
ENSRNOG00000025459	XM_216910	MARS	methionine-tRNA synthetase (predicted)	0,61	0,13	0,08
ENSRNOG00000020426	XM_218336	Erf	Ets2 repressor factor	0,61	0,53	0,87
ENSRNOG00000016760	XM_226403	KCTD19	similar to hypothetical protein MGC2628	0,61	0,30	0,45
ENSRNOG00000025327	NM_053423	Tert	telomerase reverse transcriptase	0,61	0,23	0,19
ENSRNOG00000009754	NM_177928	Nampt	nicotinamide phosphoribosyltransferase	0,61	0,48	0,71
ENSRNOG00000027263	XM_001076509.3	RGD1562550	PREDICTED: Rattus norvegicus transmembrane protein C5orf28 homolog (RGD1562550)	0,61	0,44	0,63
ENSRNOG00000021012	XM_227426	RFX5	regulatory factor X, 5 (influences HLA class II expression) (predicted)	0,61	0,40	0,63
ENSRNOG00000026559	XM_214440	XM_214440	protein tyrosine phosphatase domain containing 1 (predicted)	0,60	0,31	0,45
ENSRNOG00000023449	Rn.125816	ATG9B	ATG9 autophagy related 9 homolog B (S. cerevisiae)	0,60	0,19	0,11
ENSRNOG00000019369	NM_001009292	Giyd2	GIY-YIG domain containing 2	0,60	0,21	0,19
ENSRNOG00000021483	XM_226563	XM_226563	similar to TRIM9-like protein TNL	0,60	0,19	0,11
ENSRNOG00000009090	NM_138910	DAD1	defender against cell death 1	0,60	0,27	0,38
ENSRNOG00000019956	XM_233441	JMJD2A	jumonji domain containing 2A (predicted)	0,60	0,35	0,51
ENSRNOG00000008067	XM_234546	Rcor1	REST corepressor 1	0,60	0,40	0,63
ENSRNOG00000018648	XM_341627	Mppe1	metallophosphoesterase 1	0,60	0,23	0,26
ENSRNOG00000019651	NM_019229	Slc12a4	solute carrier family 12 (potassium/chloride transporters), member 4	0,60	0,11	0,00
ENSRNOG00000015082	XM_219247	Nalp10	NACHT, leucine rich repeat and PYD containing 10 (predicted)	0,60	0,20	0,11
ENSRNOG00000027490	NM_001000191	NM_001000191	olfactory receptor gene Olr203	0,60	0,30	0,38
ENSRNOG00000009709	Rn.140607	LOC287569	similar to FLJ34922 protein	0,60	0,13	0,08
ENSRNOG00000018751	XM_215051	Cog7	component of oligomeric golgi complex 7	0,60	0,28	0,38
ENSRNOG00000006787	XM_216452	Dhcr24	24-dehydrocholesterol reductase	0,60	0,37	0,51
ENSRNOG00000010550	NM_001008376	Trappc3	trafficking protein particle complex 3	0,60	0,31	0,45
ENSRNOG00000002390	NM_130734	gnb2l1	guanine nucleotide binding protein (G protein), beta polypeptide 2 like 1	0,60	0,32	0,45
ENSRNOG00000018074	NM_133295.3	Ces1d	carboxylesterase 1D (Ces1d)	0,60	0,22	0,19
ENSRNOG00000019837	XM_343482	XM_343482	similar to GDP-mannose pyrophosphorylase B isoform 2; mannose-1-phosphate guanylyltransferase; amphoterin in	0,60	0,12	0,08
ENSRNOG00000022967	XM_216462	Slc35d1	solute carrier family 35 (UDP-glucuronic acid/UDP-N-acetylgalactosamine dual transporter), member D1	0,60	0,19	0,11
ENSRNOG00000011875	NM_012879	Slc2A2	solute carrier family 2 (facilitated glucose transporter), member 2	0,60	0,26	0,38
ENSRNOG00000015302	XM_343472	Cis	Cytokine-inducible SH2-containing protein (CIS) (CIS-1) (SOCS)	0,60	0,57	1,22
ENSRNOG00000021343	XM_228529	LOC680578	similar to hypothetical protein FLJ13611	0,60	0,24	0,26
ENSRNOG00000016692	XM_232942	hsdl2	hydroxysteroid dehydrogenase like 2	0,60	0,59	0,92
ENSRNOG00000018886	1110018J18Rik	LOC498685	RIKEN cDNA 1110018J18 gene	0,60	0,22	0,19
ENSRNOG00000019705	NM_017143	nd	coagulation factor 10	0,60	0,22	0,19
ENSRNOG00000028144	NM_022504	nd	ribosomal protein L36	0,60	0,29	0,38
ENSRNOG00000023468	NM_020098	NM_020098	piccolo (presynaptic cytomatrix protein)	0,60	0,40	0,63
ENSRNOG00000020546	NM_012859	Lipe	lipase, hormone sensitive	0,60	0,19	0,19
ENSRNOG00000026140	XM_213131	XM_213131	similar to 60S RIBOSOMAL PROTEIN L13	0,60	0,09	0,00
ENSRNOG00000019754	XM_003750951.1	Ttyh2	tweety homolog 2 (Drosophila), transcript variant 1 (Ttyh2)	0,60	0,31	0,45
ENSRNOG00000013292	XM_003750647.1	LOC100910336	PREDICTED: Rattus norvegicus 40S ribosomal protein S19-like (LOC100910336)	0,60	0,16	0,11
ENSRNOG00000009005	NM_053557.1	Prmt3	protein arginine methyltransferase 3 (Prmt3)	0,60	0,40	0,63

ENSRNOG00000026963	NM_001012197	Tra1	tumor rejection antigen gp96	0,60	0,32	0,45
ENSRNOG00000019335	NM_001012199	NM_001012199	RAN GTPase activating protein 1 (predicted)	0,59	0,25	0,26
ENSRNOG00000021133	XM_341841	Lsm14a	LSM14A, SCD6 homolog A (S. cerevisiae)	0,59	0,33	0,45
ENSRNOG00000015236	NM_031668	Mybbp1a	MYB binding protein (P160) 1a	0,59	0,30	0,45
ENSRNOG00000022655	ENSRNOG00000022655	LOC501166	similar to Hnrpa3 protein	0,59	0,21	0,19
ENSRNOG00000026109	NM_024363	PRMT1	protein arginine methyltransferase 1	0,59	0,13	0,08
ENSRNOG00000001288	NM_001109062.1	Gpr146	G protein-coupled receptor 146 (Gpr146)	0,59	0,10	0,00
ENSRNOG00000008577	XM_213526	FBF1	Fas (TNFRSF6) binding factor 1 (predicted)	0,59	0,29	0,45
ENSRNOG00000020599	XM_224740	Tssk6	testis-specific serine kinase 6	0,59	0,41	0,63
ENSRNOG00000006331	NM_134382	Elovl5	ELOVL family member 5, elongation of long chain fatty acids (yeast)	0,59	0,15	0,11
ENSRNOG00000015460	XM_217458	LOC691066	transmembrane 4 L six family member 20	0,59	0,36	0,51
ENSRNOG00000020635	XM_218343	Exosc5	exosome component 5	0,59	0,34	0,51
ENSRNOG00000018166	NM_022627	PRKAB2	protein kinase, AMP-activated, beta 2 non-catalytic subunit	0,59	0,13	0,11
ENSRNOG00000016975	NM_172223	Pxmp4	peroxisomal membrane protein 4	0,59	0,57	1,22
ENSRNOG00000015699	XM_217385	LMAN2L	lectin, mannose-binding 2-like (predicted)	0,59	0,45	0,71
ENSRNOG00000025185	XM_345335	Nup188	nucleoporin 188	0,59	0,28	0,45
ENSRNOG00000009458	XM_221202	nd	similar to mitochondrial ribosomal protein L12	0,59	0,20	0,19
ENSRNOG00000020011	NM_001013072	sfxn2	sideroflexin 2	0,59	0,44	0,71
ENSRNOG00000010728	NM_001109307.2	Stradb	STE20-related kinase adaptor beta (Stradb)	0,59	0,28	0,45
Down-regulated genes						
ENSRNOG00000000033	XM_223107	Tmcc2	transmembrane and coiled-coil domain family 2	-0,59	0,19	0,26
ENSRNOG00000005905	NM_017031	Pde4b	phosphodiesterase 4B, cAMP specific	-0,59	0,53	1,71
ENSRNOG00000000983	NM_145724	ZNF394	zinc finger protein 394	-0,59	0,20	0,26
ENSRNOG00000011630	NM_017135	Ak3l1	adenylate kinase 3-like 1	-0,59	0,26	0,45
ENSRNOG00000018214	NM_017312	Bok	BCL2-related ovarian killer	-0,59	0,12	0,13
ENSRNOG00000013291	NM_031839	Cyp2c23	cytochrome P450 2C23	-0,59	0,42	0,71
ENSRNOG00000002397	XM_223046	Slc30a10	solute carrier family 30, member 10	-0,59	0,08	0,00
ENSRNOG00000017150	XM_214417	MED10	mediator complex subunit 10	-0,59	0,30	0,51
ENSRNOG00000019467	NM_001106534.2	Fer1l4	fer-1-like 4 (C. elegans) (Fer1l4)	-0,59	0,08	0,00
ENSRNOG00000028517	NM_001007649	MGC94282	similar to 5930416I19Rik protein	-0,59	0,28	0,45
ENSRNOG00000016648	NM_022220	Gulo	gulonolactone (L-) oxidase	-0,59	0,43	0,87
ENSRNOG00000000445	NM_001002805.2	C4b	complement component 4B (Chido blood group) (C4b)	-0,59	0,35	0,63
ENSRNOG00000016339	NM_053768	Uox	urate oxidase	-0,59	0,37	0,63
ENSRNOG00000009977	NM_001009693	Thrap3	thyroid hormone receptor associated protein 3	-0,59	0,20	0,26
ENSRNOG00000010788	NM_001100851.1	Kmt2e	lysine (K)-specific methyltransferase 2E (Kmt2e)	-0,59	0,15	0,13
ENSRNOG00000002100	XM_214017	Srp72	similar to signal recognition particle,72 kDa subunit	-0,59	0,15	0,13
ENSRNOG00000003370	NM_013078	OTC	ornithine carbamoyltransferase	-0,59	0,26	0,38
ENSRNOG00000011413	NM_138508	scp2	sterol carrier protein 2	-0,59	0,44	0,87
ENSRNOG00000001804	NM_031046	Itpr2	inositol 1,4,5-triphosphate receptor, type 2	-0,60	0,15	0,13
ENSRNOG00000002203	XM_223187	LIN54	lin-54 homolog (C. elegans)	-0,60	0,17	0,13
ENSRNOG00000014376	NM_012803	Proc	protein C	-0,60	0,23	0,26
ENSRNOG00000014566	NM_001013105	FKBP11	FK506 binding protein 11	-0,60	0,07	0,00
ENSRNOG00000002258	XM_341190	RGD1306105	similar to RIKEN cDNA 2610318G18	-0,60	0,18	0,19
ENSRNOG00000017059	XM_215222	Cetn2	centrin 2	-0,60	0,18	0,19
ENSRNOG00000010895	NM_001004248	TMEM30A	transmembrane protein 30A	-0,60	0,15	0,13
ENSRNOG00000021084	Rn.162497	Mpeg1	macrophage expressed gene 1	-0,60	0,34	0,51
ENSRNOG00000001853	NM_053842	ERK-2	mitogen-activated protein kinase 1 (extracelular signal-regulated kinase 2) (P42-MAPK)	-0,60	0,22	0,26
ENSRNOG00000002636	Rn.10090	Abat	4-aminobutyrate aminotransferase	-0,60	0,18	0,13
ENSRNOG00000001736	Rn.36635	Bdh1	3-hydroxybutyrate dehydrogenase, type 1	-0,60	0,20	0,19
ENSRNOG00000018293	Rn.136895	LOC362012	family with sequence similarity 40, member A	-0,60	0,28	0,45
ENSRNOG00000008374	Rn.103205	Nipsnap1	4-nitrophenylphosphatase domain and non-neuronal SNAP25-like protein homolog 1 (C. elegans)	-0,60	0,48	0,87
ENSRNOG00000010352	Rn.162234	Dnajc3	DnaJ (Hsp40) homolog, subfamily C, member 3	-0,60	0,17	0,13
ENSRNOG00000023491	NM_001013185.1	Adck3	aarF domain containing kinase 3 (Adck3), nuclear gene encoding mitochondrial protein	-0,60	0,54	1,22
ENSRNOG00000004702	Rn.10347	Cpg1	candidate plasticity gene 1	-0,60	0,15	0,13
ENSRNOG00000017003	Rn.199986	PLXNA1	Plxna1	-0,60	0,24	0,38
ENSRNOG00000008427	Rn.56102	Necap2	NECAP endocytosis associated 2	-0,60	0,13	0,13
ENSRNOG00000010765	NM_001107248.1	Vcl	vinculin (Vcl)	-0,60	0,19	0,19
ENSRNOG00000009740	Rn.45744	Slco1c1	solute carrier organic anion transporter family, member 1c1	-0,60	0,16	0,13
ENSRNOG00000016000	Rn.3689	Atp5f1	ATP synthase, H+ transporting, mitochondrial F0 complex, subunit B1	-0,60	0,20	0,19
ENSRNOG00000023035	Rn.16772	RGD1305158	similar to RIKEN cDNA 1810030N24	-0,61	0,32	0,45
ENSRNOG00000003095	Rn.6346	Fgfbp1	fibroblast growth factor binding protein 1	-0,61	0,26	0,38
ENSRNOG00000000964	Rn.162293	Frg1	FSHD region gene 1; similar to FRG1 protein (FSHD region gene 1 protein)	-0,61	0,22	0,26
ENSRNOG00000018368	NM_001000403	Olr847	olfactory receptor 847 (Olr847)	-0,61	0,21	0,19
ENSRNOG00000013246	NM_001107317.2	Mboat4	membrane bound O-acyltransferase domain containing 4 (Mboat4)	-0,61	0,36	0,63
ENSRNOG00000023091	Rn.195964	RGD1561704	similar to 60S ribosomal protein L27a (L29)	-0,61	0,26	0,38
ENSRNOG00000022239	Rn.18571	Tusc5	tumor suppressor candidate 5	-0,61	0,27	0,38
ENSRNOG00000001301	NM_053845.1	Upb1	ureidopropionase, beta (Upb1)	-0,61	0,37	0,63
ENSRNOG00000027392	Rn.123033	RGD1565512	similar to hypothetical protein 4932418E24	-0,61	0,25	0,26
ENSRNOG00000004245	Rn.1916	Mgst3	microsomal glutathione S-transferase 3	-0,61	0,36	0,51
ENSRNOG00000018204	Rn.10140	Slc28a2	similar to solute carrier family 28, member 2; solute carrier family 28 (sodium-coupled nucleoside transporter), meml	-0,61	0,13	0,13
ENSRNOG00000014438	XM_001057586.2	Atp5hl1	PREDICTED: Rattus norvegicus ATP synthase, H+ transporting, mitochondrial Fo complex, subunit d-like 1 (Atp5hl1	-0,61	0,15	0,13
ENSRNOG00000000962	Rn.10770	Slc15a4	solute carrier family 15, member 4	-0,61	0,05	0,00
ENSRNOG00000002208	Rn.15243	Shrm3	shroom family member 3	-0,61	0,08	0,00
ENSRNOG00000019094	NM_177426	Gstm2	glutathione S-transferase mu 2	-0,61	0,20	0,19
ENSRNOG00000014540	RGD1309621	RGD1309621	PREDICTED: Rattus norvegicus similar to hypothetical protein FLJ10652 (RGD1309621)	-0,61	0,15	0,13
ENSRNOG00000017064	Rn.161910	TDRD1	tudor domain containing 1	-0,61	0,14	0,13
ENSRNOG00000026511	NM_001048075	Clec2d11	similar to osteoclast inhibitory lectin	-0,61	0,28	0,38
ENSRNOG00000015812	Rn.24712	Tm4sf1	transmembrane 4 L six family member 1	-0,61	0,20	0,19
ENSRNOG00000017156	Rn.200834	ANKLE1	Ankle1	-0,61	0,07	0,00
ENSRNOG00000015772	Rn.154877	Metap2	similar to Methionine aminopeptidase 2 (MetAP 2)	-0,62	0,12	0,08
ENSRNOG00000012946	Rn.198614	CAPZA1	Moloney leukemia virus 10	-0,62	0,19	0,19
ENSRNOG00000012091	Rn.37581	Ppa2	pyrophosphatase (inorganic) 2	-0,62	0,27	0,38
ENSRNOG00000007172	XM_343006	MAP4K3	mitogen-activated protein kinase kinase kinase kinase 3	-0,62	0,15	0,17
ENSRNOG00000005627	XM_216369.3	LOC297970	PREDICTED: Rattus norvegicus similar to LRRGT00057 (LOC297970)	-0,62	0,19	0,13

ENSRNOG00000017107	XM_226468.7	Hydin	PREDICTED: Rattus norvegicus Hydin, axonemal central pair apparatus protein (Hydin)	-0,62	0,22	0,19
ENSRNOG00000002132	XM_003751325.1	LOC100912618	PREDICTED: Rattus norvegicus ubiquitin-conjugating enzyme E2 variant 1-like (LOC100912618)	-0,62	0,29	0,38
ENSRNOG00000015989	Rn.13489	Mrpl32	39S ribosomal protein L32, mitochondrial	-0,62	0,22	0,19
ENSRNOG00000003709	Rn.35029	Kmo	Kynurenine 3-monooxygenase	-0,62	0,37	0,51
ENSRNOG00000019441	Rn.109394	Hsd3b6	hydroxy-delta-5-steroid dehydrogenase, 3 beta- and steroid delta-isomerase 6	-0,62	0,28	0,38
ENSRNOG00000003592	Rn.34860	Med28	mediator complex subunit 28	-0,62	0,12	0,08
ENSRNOG00000019585	Rn.127898	MYST1	MYST histone acetyltransferase 1	-0,62	0,16	0,13
ENSRNOG00000002783	Rn.11698	SerpinC1	serine (or cysteine) peptidase inhibitor, clade C (antithrombin), member 1	-0,63	0,20	0,13
ENSRNOG00000017872	Rn.154031	LOC688778	fatty aldehyde dehydrogenase-like	-0,63	0,21	0,19
ENSRNOG00000009330	Rn.74591	Slc17a5	solute carrier family 17 (anion/sugar transporter), member 5	-0,63	0,32	0,45
ENSRNOG00000002265	Rn.10019	Casr	calcium-sensing receptor	-0,63	0,14	0,08
ENSRNOG00000009696	Rn.90967	Mdm4	Mdm4 p53 binding protein homolog (mouse)	-0,63	0,12	0,08
ENSRNOG00000003492	Rn.17160	Gas7	growth arrest specific 7	-0,63	0,08	0,00
ENSRNOG00000012146	Rn.99801	LOC681879	protein-O-mannosyltransferase 2; serine palmitoyltransferase, long chain base subunit 2	-0,63	0,15	0,13
ENSRNOG00000025653	NM_001134886.1	Vps13b	vacuolar protein sorting 13 homolog B (yeast) (Vps13b)	-0,63	0,19	0,13
ENSRNOG00000028568	XM_002727906.3	Myh15	PREDICTED: Rattus norvegicus myosin, heavy chain 15 (Myh15)	-0,63	0,27	0,26
ENSRNOG00000016689	XM_002728762.2	Fanci	PREDICTED: Rattus norvegicus Fanconi anemia, complementation group I (Fanci)	-0,63	0,33	0,45
ENSRNOG00000006709	Rn.11295	Pzp	pregnancy-zone protein	-0,63	0,32	0,45
ENSRNOG00000001823	NM_147205	siat1	ST6 beta-galactosamide alpha-2,6-sialyltranferase 1	-0,63	0,30	0,38
ENSRNOG00000013683	Rn.109455	Edg1	sphingosine-1-phosphate receptor 1	-0,63	0,51	0,87
ENSRNOG00000001756	Rn.101441	LOC690043	UBX domain protein 7; ring finger protein 168	-0,63	0,26	0,26
ENSRNOG00000000669	NM_001108340.1	Hscb	HscB iron-sulfur cluster co-chaperone homolog (E. coli) (Hscb)	-0,63	0,21	0,13
ENSRNOG00000002236	NM_057147.2	Sec22a	SEC22 vesicle trafficking protein homolog A (S. cerevisiae) (Sec22a)	-0,63	0,21	0,13
ENSRNOG00000007218	NM_001126081.1	Leap2	liver-expressed antimicrobial peptide 2	-0,63	0,59	1,22
ENSRNOG00000019485	Rn.31976	Bckdk	branched chain ketoacid dehydrogenase kinase	-0,63	0,13	0,08
ENSRNOG00000004844	Rn.3694	Thrap6	mediator complex subunit 30	-0,63	0,17	0,13
ENSRNOG00000001056	Rn.162080	Snapc2	small nuclear RNA activating complex, polypeptide 2	-0,63	0,04	0,00
ENSRNOG00000000982	Rn.24025	Znf498	zinc finger protein 498; zinc finger protein 655; zinc finger with KRAB and SCAN domains 5	-0,64	0,13	0,08
ENSRNOG00000018760	Rn.34512	Mpp7	membrane protein, palmitoylated 7 (MAGUK p55 subfamily member 7)	-0,64	0,49	0,71
ENSRNOG00000018186	Rn.3763	Hbxip	hepatitis B virus x interacting protein	-0,64	0,16	0,08
ENSRNOG00000021407	NM_001135600.1	Cyp4v3	cytochrome P450, family 4, subfamily v, polypeptide 3 (Cyp4v3)	-0,64	0,49	0,87
ENSRNOG00000020748	Rn.211012	MAP4	microtubule-associated protein 4	-0,64	0,19	0,13
ENSRNOG00000019058	NM_020540	GST	glutathione S-transferase mu 3; GST mu 4; GST mu 7	-0,64	0,06	0,00
ENSRNOG00000000981	Rn.29435	Cd36l1	scavenger receptor class B, member 1	-0,64	0,19	0,13
ENSRNOG00000028545	Rn.162901	RGD1309696	similar to KIAA2019 protein	-0,64	0,26	0,26
ENSRNOG00000006423	Rn.129677	cycA	peptidylprolyl isomerase A (cyclophilin A) (P31) (p1B15)	-0,64	0,23	0,19
ENSRNOG00000013118	Rn.11992	Atox1	ATX1 antioxidant protein 1 homolog (yeast)	-0,65	0,42	0,63
ENSRNOG00000003626	NM_019383	atp5h	ATP synthase D chain, mitochondrial	-0,65	0,18	0,13
ENSRNOG00000004685	Rn.94848	MTA3	metastasis associated 1 family, member 3	-0,65	0,20	0,13
ENSRNOG00000002104	NM_001037347	SFRS15	splicing factor, arginine/serine-rich 15	-0,65	0,17	0,17
ENSRNOG00000014739	NM_173109	Wfdc2	WAP four-disulfide core domain 2	-0,65	0,72	2,38
ENSRNOG00000000453	NM_001004084.2	RT1-Bb	RT1 class II, locus Bb (RT1-Bb)	-0,65	0,31	0,38
ENSRNOG00000012121	Rn.3965	Sdf2	stromal cell derived factor 2	-0,65	0,27	0,19
ENSRNOG00000002176	Rn.56373	Pvrl3	poliovirus receptor-related 3	-0,65	0,16	0,08
ENSRNOG00000004329	NM_199208.1	Rdh16	retinol dehydrogenase 16 (all-trans) (Rdh16)	-0,66	0,14	0,00
ENSRNOG00000017776	XM_003752949.1	LOC100912731	PREDICTED: Rattus norvegicus zinc finger protein 120-like (LOC100912731)	-0,66	0,17	0,08
ENSRNOG00000008421	Rn.13334	Klhl5	kelch-like 5 (Drosophila)	-0,66	0,28	0,26
ENSRNOG00000014664	Rn.102580	RGD1559904	1110037F02Rik	-0,66	0,17	0,00
ENSRNOG00000008050	Rn.38669	Stac3	SH3 and cysteine rich domain 3	-0,66	0,95	4,78
ENSRNOG00000028494	XR_147004.1	LOC366782	PREDICTED: Rattus norvegicus 60S ribosomal protein L7-like (LOC366782)	-0,66	0,27	0,19
ENSRNOG00000002878	Rn.214035	Afm	afamin	-0,66	0,33	0,38
ENSRNOG00000007177	Rn.98686	Kdelc2	KDEL (Lys-Asp-Glu-Leu) containing 2	-0,66	0,14	0,00
ENSRNOG00000001061	Rn.12933	MGC94142	Rab interacting lysosomal protein-like 2	-0,66	0,13	0,00
ENSRNOG00000001730	Rn.198269	Centb2	ArfGAP with coiled-coil, ankyrin repeat and PH domains 2	-0,67	0,30	0,26
ENSRNOG00000007233	Rn.3472	Qpc	ubiquinol-cytochrome c reductase, complex III subunit VII	-0,67	0,18	0,08
ENSRNOG00000020035	Rn.10172	Cyp17a1	cytochrome P450 17A1	-0,67	0,86	2,99
ENSRNOG00000018340	Rn.68511	Marveld2	MARVEL domain containing 2	-0,67	0,19	0,08
ENSRNOG00000015660	Rn.11773	Pex3	peroxisomal biogenesis factor 3	-0,67	0,49	0,71
ENSRNOG00000019466	Rn.55456	Agpat2	1-acylglycerol-3-phosphate O-acyltransferase 2 (lysophosphatidic acid acyltransferase, beta)	-0,67	0,10	0,00
ENSRNOG00000013296	XM_215562	mgst2	microsomal glutathione S-transferase 2	-0,67	0,22	0,11
ENSRNOG00000002240	Rn.137795	Dirc2	disrupted in renal carcinoma 2 (human)	-0,67	0,23	0,13
ENSRNOG00000012904	NM_001108792.1	Bend6	BEN domain containing 6 (Bend6)	-0,67	0,19	0,13
ENSRNOG00000003941	NM_057114.1	Prdx1	peroxiredoxin 1 (Prdx1)	-0,67	0,16	0,08
ENSRNOG00000002182	NM_001107208.2	Sept11	septin-11 (Sept11)	-0,67	0,17	0,08
ENSRNOG00000000451	Rn.25717	HLA-DMB	RT1 class II, locus Ba	-0,67	0,43	0,51
ENSRNOG00000013704	Rn.53968	Cps1	carbamoyl-phosphate synthetase 1, mitochondrial	-0,67	0,30	0,26
ENSRNOG00000017820	Rn.7882	Nqo2	NAD(P)H dehydrogenase, quinone 2	-0,67	0,03	0,00
ENSRNOG00000015518	Rn.108214	Rbp4	retinol binding protein 4, plasma	-0,67	0,27	0,19
ENSRNOG00000018267	Rn.165018	B3GNT7	UDP-GlcNAc:betaGal beta-1,3-N-acetylglucosaminyltransferase 7	-0,67	0,28	0,19
ENSRNOG00000001388	Rn.9918	Sds	serine dehydratase	-0,68	0,27	1,25
ENSRNOG00000000901	Rn.9449	Crcp	calcitonin gene-related peptide-receptor component protein	-0,68	0,29	0,19
ENSRNOG00000019924	Rn.9490	THOP1	thimet oligopeptidase 1	-0,68	0,69	1,71
ENSRNOG00000007142	Rn.88274	B3gat1	beta-1,3-glucuronyltransferase 1 (glucuronosyltransferase P)	-0,68	0,16	0,08
ENSRNOG00000004679	Rn.67791	Fign	fidgetin	-0,68	0,15	0,00
ENSRNOG00000024005	XM_001058335.3	LOC501421	PREDICTED: Rattus norvegicus hypothetical gene supported by BC082068 (LOC501421)	-0,68	0,45	0,63
ENSRNOG00000007793	Rn.10967	Pnrc1	proline-rich nuclear receptor coactivator 1	-0,68	0,35	0,38
ENSRNOG00000017843	Rn.92563	Polr3k	polymerase (RNA) III (DNA directed) polypeptide K, 12.3 kDa	-0,68	0,11	0,00
ENSRNOG00000003624	XM_216369.3	LOC297970	PREDICTED: Rattus norvegicus similar to LRRGT00057 (LOC297970)	-0,68	0,35	0,38
ENSRNOG00000026078	NM_001000238	Olr302	olfactory receptor 302 (Olr302)	-0,68	0,45	0,51
ENSRNOG00000026487	XM_001078374.2	LOC686736	PREDICTED: Rattus norvegicus histone H3.3-like, transcript variant 1 (LOC686736)	-0,68	0,12	0,00
ENSRNOG00000001426	Rn.44767	LOC498171	Prkr interacting protein 1 (IL11 inducible)	-0,69	0,20	0,08
ENSRNOG00000007241	XM_230291	FNBP4	formin binding protein 4	-0,69	0,13	0,00
ENSRNOG00000002212	Rn.25104	Hsd17b13	hydroxysteroid (17-beta) dehydrogenase 13	-0,69	0,11	0,00
ENSRNOG00000025510	NM_198776	LOC100134871	beta-globin; beta globin minor gene	-0,69	0,40	0,45

ENSRNOG00000018722	NM_001109569.1	Wdr74	WD repeat domain 74 (Wdr74)	-0,69	0,86	2,99
ENSRNOG00000027441	XM_003752050.1	LOC100911008	PREDICTED: Rattus norvegicus histone-lysine N-methyltransferase MLL3-like (LOC100911008)	-0,69	0,94	3,73
ENSRNOG00000011864	Rn.98783	Gm2a	GM2 ganglioside activator	-0,69	0,38	0,38
ENSRNOG00000012971	NM_130409.2	Cfh	complement factor H (Cfh), nuclear gene encoding mitochondrial protein	-0,69	0,26	0,13
ENSRNOG00000022588	Rn.145502	Akr1c12	aldo-keto reductase family 1, member C12	-0,70	0,11	0,00
ENSRNOG00000003606	Rn.195622	RGD1561381	similar to microsomal glutathione S-transferase 3	-0,70	0,43	0,45
ENSRNOG00000011184	Rn.162743	Slc13a4	solute carrier family 13 (sodium/sulfate symporters), member 4	-0,70	0,23	0,13
ENSRNOG00000000955	Rn.162084	Lnx2	ligand of numb-protein X 2	-0,70	0,23	0,13
ENSRNOG00000025625	Rn.1742	Rnase4	ribonuclease, RNase A family 4	-0,71	0,13	0,00
ENSRNOG00000001926	Rn.24293	Cldn1	claudin 1	-0,71	0,36	0,26
ENSRNOG00000022992	NM_001024753.1	Ifit2	interferon-induced protein with tetratricopeptide repeats 2 (Ifit2)	-0,71	0,15	0,00
ENSRNOG00000015774	Rn.163080	MREG	melanoregulin	-0,71	0,43	0,45
ENSRNOG00000000533	NM_001025727.1	Rnf8	ring finger protein 8, E3 ubiquitin protein ligase (Rnf8)	-0,72	0,34	0,26
ENSRNOG00000020793	NM_001025405	RUVBL2	RuvB-like 2 (E. coli); similar to RuvB-like 2; similar to RuvB-like 2 (p47 protein)	-0,72	0,25	0,13
ENSRNOG00000003253	Rn.241	Qdpr	quinoid dihydropteridine reductase	-0,72	0,42	0,38
ENSRNOG00000005437	Rn.6987	Hrsp12	heat-responsive protein 12	-0,72	0,15	0,00
ENSRNOG00000000931	XM_222111	SFRS8	splicing factor, arginine/serine-rich 8	-0,72	0,17	0,00
ENSRNOG00000028587	XM_003752566.1	LOC100911504	PREDICTED: Rattus norvegicus uncharacterized LOC100911504 (LOC100911504)	-0,72	0,24	0,13
ENSRNOG00000002234	XM_223390.7	Atp8a1	PREDICTED: Rattus norvegicus ATPase, aminophospholipid transporter (APLT), class I, type 8A, member 1 (Atp8a1	-0,72	0,23	0,08
ENSRNOG00000001000	NM_001105907.2	Trrap	transformation/transcription domain-associated protein (Trrap)	-0,72	0,20	0,08
ENSRNOG00000001852	XM_216309	RGD1310606	Ergic2	-0,72	0,22	0,08
ENSRNOG00000017063	Rn.20051	Fcna	ficolin (collagen/fibrinogen domain containing) 1	-0,73	0,12	0,00
ENSRNOG00000019556	Rn.2091	Cd9	CD9 molecule	-0,73	0,39	0,26
ENSRNOG00000002289	Rn.178819	ILDR1	immunoglobulin-like domain containing receptor 1	-0,74	0,11	0,00
ENSRNOG00000005513	NM_019257	SRP40	splicing factor, arginine/serine-rich 5	-0,74	0,24	0,08
ENSRNOG00000003142	Rn.154415	Fcgr3	Fc fragment of IgG, low affinity IIb, receptor (CD32); Fc fragment of IgG, low affinity IIa, receptor (CD32)	-0,75	0,24	0,08
ENSRNOG00000019425	Rn.189363	GABARAPL2	GABA(A) receptor-associated protein like 2	-0,75	0,27	0,13
ENSRNOG00000028563	XM_216369.3	LOC297970	PREDICTED: Rattus norvegicus similar to LRRGT00057 (LOC297970)	-0,75	0,31	0,13
ENSRNOG00000000534	NM_001025059	TBC1D22B	TBC1 domain family, member 22B	-0,75	0,18	0,00
ENSRNOG00000020676	Rn.73852	Ppp1r14a	protein phosphatase 1, regulatory (inhibitor) subunit 14A	-0,75	0,06	0,00
ENSRNOG00000018742	Rn.10732	Klk11	kallikrein related-peptidase 11	-0,75	0,63	0,71
ENSRNOG00000025815	Rn.145144	CDR2L	cerebellar degeneration-related protein 2-like	-0,76	0,61	0,71
ENSRNOG00000014155	XM_215564	spg20	spastic paraplegia 20, spartin (Troyer syndrome) homolog (human)	-0,76	0,28	0,13
ENSRNOG00000003662	NM_024381	GK	glycerol kinase (ATP:Glycerol 3-phosphotransferase) (glycerokinase) (GK) (ASTP)	-0,76	0,36	0,19
ENSRNOG00000019482	Rn.90161	Gnao	guanine nucleotide binding protein (G protein), alpha activating activity polypeptide O	-0,77	0,04	0,00
ENSRNOG00000027448	XM_243815.5	Foxr1	PREDICTED: Rattus norvegicus forkhead box R1 (Foxr1)	-0,77	0,47	0,38
ENSRNOG00000018735	Rn.193175	CD74	Cd74 molecule, major histocompatibility complex, class II invariant chain	-0,77	0,47	0,49
ENSRNOG00000016660	Rn.6686	Cox5b	cytochrome c oxidase subunit Vb	-0,77	0,48	0,45
ENSRNOG00000016286	Rn.34836	IYD	iodotyrosine deiodinase	-0,77	0,52	0,45
ENSRNOG00000026116	Rn.99341	Sall3	sal-like 3 (Drosophila)	-0,78	0,19	0,00
ENSRNOG00000004147	XM_221100	ABCA-1	ATP-binding cassette, sub-family A (ABC1), member 8a	-0,78	0,36	0,13
ENSRNOG00000005669	Rn.22066	Car8	carbonic anhydrase 8	-0,78	0,30	0,13
ENSRNOG00000019414	Rn.13463	TMEM79	transmembrane protein 79	-0,78	0,07	0,00
ENSRNOG00000001344	Rn.101781	Aldh2	aldehyde dehydrogenase 2 family (mitochondrial)	-0,78	0,24	0,00
ENSRNOG00000014806	Rn.50364	PNKD	Pnkd	-0,78	0,33	0,13
ENSRNOG00000017531	Rn.10030	Akr1c18	aldo-keto reductase family 1, member C18	-0,78	0,36	0,19
ENSRNOG00000014610	Rn.11132	Anpep	alanyl (membrane) aminopeptidase	-0,78	0,21	0,00
ENSRNOG00000010906	Rn.8019	Ccl5	chemokine (C-C motif) ligand 5	-0,79	0,31	0,13
ENSRNOG00000018701	Rn.163109	Tpsg1	tryptase gamma 1	-0,80	0,78	0,87
ENSRNOG00000005931	Rn.165717	Pgcp	plasma glutamate carboxypeptidase	-0,80	0,26	0,00
ENSRNOG00000007258	Rn.144410	RPESP	RPE-spondin	-0,81	0,24	0,00
ENSRNOG00000017619	Rn.6132	ALDH2	aldehyde dehydrogenase 1 family, member A1	-0,81	0,24	0,00
ENSRNOG00000014635	Rn.112599	Clta	clathrin, light chain (Lca)	-0,81	0,27	0,00
ENSRNOG00000000025	NM_131906.1	Slco1a2	solute carrier organic anion transporter family, member 1A2 (Slco1a2)	-0,82	0,21	0,00
ENSRNOG00000000814	NM_030832	FABP7	fatty acid binding protein 7, brain	-0,82	0,80	0,87
ENSRNOG00000009096	Rn.16469	Pon3	paraoxonase 3	-0,82	0,10	0,00
ENSRNOG00000000064	NM_080481	ATP5IAtp5k	ATP synthase, H+ transporting, mitochondrial F0 complex, subunit E	-0,82	0,13	0,00
ENSRNOG00000016271	Rn.107335	Itm2b	integral membrane protein 2B	-0,82	0,06	0,00
ENSRNOG00000007507	NM_001100963.1	Aass	aminoadipate-semialdehyde synthase (Aass), nuclear gene encoding mitochondrial protein	-0,82	0,52	0,38
ENSRNOG00000027508	NM_001108906.1	Chmp2a	charged multivesicular body protein 2A (Chmp2a)	-0,82	0,65	0,63
ENSRNOG00000011794	Rn.11547	Tcea3	transcription elongation factor A (SII), 3	-0,82	0,71	0,71
ENSRNOG00000016641	Rn.103609	Actl7a	actin-like 7a	-0,83	0,44	0,19
ENSRNOG00000006042	Rn.198813	Rup2	urinary protein 2	-0,83	0,76	0,87
ENSRNOG00000005612	NM_019287	ApoB	apolipoprotein B (fragment)	-0,83	0,32	0,08
ENSRNOG00000009019	Rn.9968	Slc6a6	solute carrier family 6 (neurotransmitter transporter, taurine)	-0,83	0,40	0,13
ENSRNOG00000001062	NM_001246665.1	Setd8	SET domain containing (lysine methyltransferase) 8 (Setd8)	-0,83	0,28	0,00
ENSRNOG00000014568	Rn.2867	LOC681418	NADH dehydrogenase (ubiquinone) 1 beta subcomplex, 10	-0,84	0,23	0,00
ENSRNOG00000001060	Rn.79076	SNRNP35	U11/U12 snRNP 35K	-0,84	0,18	0,00
ENSRNOG00000016684	Rn.30163	RGD1307284	similar to protein kinase, lysine deficient 1; kinase deficient protein	-0,85	0,49	0,26
ENSRNOG00000014604	Rn.1129	Oprs1	opioid receptor, sigma 1	-0,85	0,37	0,13
ENSRNOG00000019982	Rn.14691	Ethe1	ethylmalonic encephalopathy 1	-0,86	0,68	0,51
ENSRNOG00000001851	Rn.162778	FAR2	fatty acyl CoA reductase 2	-0,86	0,75	0,71
ENSRNOG00000019532	Rn.1470	MGC93975	similar to 2310044H10Rik protein	-0,86	0,44	0,13
ENSRNOG00000016620	Rn.10082	Csnk1g1	casein kinase 1, gamma 1	-0,87	0,48	0,19
ENSRNOG00000017223	Rn.20178	Plg	plasminogen	-0,88	0,25	0,00
ENSRNOG00000010935	Rn.12572	Cpb2	carboxypeptidase B2 (plasma)	-0,88	0,36	0,08
ENSRNOG00000003357	Rn.3247	Col3a1	collagen, type III, alpha 1	-0,89	0,31	0,00
ENSRNOG00000012929	Rn.18535	Wsb1	WD repeat and SOCS box-containing 1	-0,90	0,56	0,26
ENSRNOG00000017891	NM_134380.1	Ust5r	integral membrane transport protein UST5r (Ust5r)	-0,90	0,66	0,45
ENSRNOG00000002210	Rn.102059	Dhrs8	hydroxysteroid (17-beta) dehydrogenase 11	-0,90	0,23	0,00
ENSRNOG00000014766	Rn.102781	Galt	galactose-1-phosphate uridylyltransferase	-0,92	0,30	0,19
ENSRNOG00000001827	Rn.131140	Masp1	mannan-binding lectin serine peptidase 1	-0,93	0,28	0,00
ENSRNOG00000018937	Rn.6036	GST m3	glutathione S-transferase mu 3; GST mu 4; GST mu 7	-0,94	0,22	0,00
ENSRNOG00000024745	Rn.107041	LOC259245	alpha 2u globulin; major urinary protein 5 (MUP)	-0,94	1,08	1,22

ENSRNOG00000025764	NM_138826	MT1A	Metallothionein-1	-0,94	1,02	1,22
ENSRNOG00000002280	XM_217566	SH3BGRL	SH3 domain binding glutamic acid-rich protein like	-0,95	0,16	0,00
ENSRNOG00000017562	NM_001033958.2	Obp3	alpha-2u globulin PGCL4 (Obp3), transcript variant 2	-0,95	0,59	0,26
ENSRNOG00000003515	Rn.3603	Ephx1	epoxide hydrolase 1, microsomal	-0,95	0,39	0,04
ENSRNOG00000017236	NM_001033697.1	Akr1c1	aldo-keto reductase family 1, member C1 (Akr1c1)	-0,96	0,88	1,08
ENSRNOG00000015740	Rn.101837	RNF125	ring finger protein 125	-0,96	0,43	0,08
ENSRNOG00000027489	Rn.141215	MN1	meningioma 1	-0,96	0,86	0,63
ENSRNOG00000008638	NM_001025065	Angptl3	angiopoietin-like 3	-0,97	0,15	0,00
ENSRNOG00000019470	Rn.198611	Hao2	hydroxyacid oxidase 2 (long chain)	-0,98	0,40	0,00
ENSRNOG00000011269	Rn.9937	Sult1c1	sulfotransferase family, cytosolic, 1C, member 3	-0,98	0,43	0,08
ENSRNOG00000027129	NM_001109356.1	Sdr16c6	short chain dehydrogenase/reductase family 16C, member 6 (Sdr16c6)	-0,98	1,13	1,22
ENSRNOG00000004725	Rn.6044	Ela1	chymotrypsin-like elastase family, member 1	-0,99	0,13	0,00
ENSRNOG00000019459	Rn.266	ODC-az1	ornithine decarboxylase antizyme 1	-0,99	0,30	0,00
ENSRNOG00000008364	Rn.3001	Cat	catalase	-0,99	0,34	0,00
ENSRNOG00000010658	Rn.3881	Cth	cystathionase (cystathionine gamma-lyase)	-1,00	0,57	0,13
ENSRNOG00000016635	XM_003751985.1	LOC100910532	PREDICTED: Rattus norvegicus uncharacterized LOC100910532 (LOC100910532)	-1,00	0,69	0,26
ENSRNOG00000016614	NM_023101.1	Vti1a	vesicle transport through interaction with t-SNAREs homolog 1A (yeast)	-1,01	0,57	0,13
ENSRNOG00000006388	Rn.21399	Pygl	phosphorylase, glycogen, liver	-1,03	0,41	0,00
ENSRNOG00000005109	Rn.45197	Rprm	reprimo, TP53 dependent G2 arrest mediator candidate	-1,03	0,26	0,00
ENSRNOG00000013736	Rn.10252	C9	complement component 9	-1,03	0,13	0,00
ENSRNOG00000014565	Rn.162464	Lcmt1	leucine carboxyl methyltransferase 1	-1,03	0,09	0,00
ENSRNOG00000018756	NM_138826	MT	Metallothionein-I (MT-I)	-1,03	1,03	0,71
ENSRNOG00000005604	NM_019287	Apo	apolipoprotein BL (fragment)	-1,03	0,36	0,00
ENSRNOG00000009301	NM_001010921	GST	Glutathione S-transferase 8 (GST 8-8) (CHAIN 8) (GST class-alpha)	-1,06	0,38	0,00
ENSRNOG00000010040	NM_001012122.1	Tymp	thymidine phosphorylase	-1,09	0,19	0,00
ENSRNOG00000012458	Rn.1372	Cyp2e1	cytochrome P450 2E1	-1,09	0,32	0,00
ENSRNOG00000013004	Rn.25716	Akr1d1	aldo-keto reductase family 1, member D1 (delta 4-3-ketosteroid-5-beta-reductase)	-1,11	0,50	0,00
ENSRNOG00000001809	Rn.16100	Hrg	histidine-rich glycoprotein; similar to histidine-rich glycoprotein	-1,11	0,53	0,00
ENSRNOG00000020528	NM_001007722	Hba2	alpha-2-globin chain	-1,12	0,89	0,45
ENSRNOG00000018275	Rn.100336	Errfi1	ERBB receptor feedback inhibitor 1	-1,14	0,73	0,19
ENSRNOG00000025660	NM_138514	CYP2C13	Cytochrome P450 2C13, male-specific (CYPIIC13)	-1,14	0,41	0,00
ENSRNOG00000018237	Rn.87063	GST p2	glutathione-S-transferase, pi 1	-1,15	0,29	0,00
ENSRNOG00000011200	Rn.11406	Bhmt	betaine-homocysteine methyltransferase	-1,16	0,93	0,38
ENSRNOG00000020817	Rn.9867	Cyp2a2	cytochrome P450 2A1	-1,16	0,05	0,00
ENSRNOG00000002304	XM_002724613.2	Tnfrsf13b	PREDICTED: Rattus norvegicus tumor necrosis factor receptor superfamily, member 13B (Tnfrsf13b)	-1,17	0,11	0,00
ENSRNOG00000023116	Rn.152690	TMEM195	transmembrane protein 195	-1,20	0,13	0,00
ENSRNOG00000013464	Rn.9767	Spink1	serine peptidase inhibitor, Kazal type 3; serine peptidase inhibitor, Kazal type 1	-1,23	0,30	0,00
ENSRNOG00000000978	NM_153312	CP3A2	cytochrome P450 3A1 (CYPIIIA1) (P450-PCN1)	-1,26	0,30	0,00
ENSRNOG00000000768	NM_053299	UBD	ubiquitin D	-1,26	0,87	0,19
ENSRNOG00000005861	Rn.185798	Hsd11b1	hydroxysteroid 11-beta dehydrogenase 1	-1,27	0,31	0,00
ENSRNOG00000013279	NM_031841	Scd2	Acyl-CoA desaturase 2 (Stearoyl-CoA desaturase 2) (Fatty acid desaturase 2) (Delta(9)-desaturase 2)	-1,30	1,41	0,71
ENSRNOG00000020529	NM_013096.1	Hba1	hemoglobin, alpha 1 (Hba1)	-1,33	0,33	0,00
ENSRNOG00000009273	NM_001033959.2	Obp3	alpha-2u globulin PGCL4 (Obp3), transcript variant 3	-1,38	1,01	0,19
ENSRNOG00000000969	Rn.32085	Cyp3a18	cytochrome P450 3A18	-1,43	0,59	0,00
ENSRNOG00000023986	XM_003754685.1	LOC100912405	urinary protein 3-like (LOC100912405)	-1,47	0,58	0,00
ENSRNOG00000019417	Rn.197811	Hsd3b5	3 beta-hydroxysteroid dehydrogenase type 3 (3Beta-HSD III)	-1,52	0,60	0,00
ENSRNOG00000009438	Rn.2374	Serpina6	serine (or cysteine) peptidase inhibitor, clade A, member 6	-1,54	0,77	0,00
ENSRNOG00000013410	NM_152936.1	Spink1	serine peptidase inhibitor, Kazal type 1 (Spink1)	-2,36	0,76	0,00
ENSRNOG00000010079	NM_019292	Ca3	carbonic anhydrase 3	-3,15	0,60	0,00
ENSRNOG00000001957	NM_001007718	Ste	sulfotransferase family 1E; estrogen sulfotransferase	-3,80	0,68	0,00

Table S2. Hepatic genes regulated by E2 in hypothyroid-orchidectomized rats. The administrations of vehicle (TXOX) or E2 (TXOXE2) to TXOX rats were described in Material and Methods. Then, differently expressed genes in the livers were identified by DNA microarrays. The analysis is based on the SAM statistical technique and differentially-expressed genes were discovered using a FDR less than 5% and a mean ratio of log2 > |0.58|. The table shows ENSEMBL gene ID, Unigene/Refseq, gene symbol, gene description, R (TXOXE2/TXOX), SD, and q(%).
