# Supplementary material for: Lipid Profiling and Transcriptomic Analysis Reveals a Functional Interplay between Estradiol and Growth Hormone in Liver
Source: PLoS One. 2014 May 9;9(5):e96305. doi: 10.1371/journal.pone.0096305 (PMC4015979; doi:10.1371/journal.pone.0096305)
Supplement: Table S3 — Hepatic genes regulated by GH in hypothyroid-orchidectomized rats. The administrations of vehicle (TXOX) or GH (TXOXGH) to TXOX rats were described in Material and Methods. Then, differently expressed genes in the livers were identified by DNA microarrays. The analysis is based on the SAM statistical technique and differentially-expressed genes were discovered using a FDR less than 5% and a mean ratio of log2>|0.58|. The table shows ENSEMBL gene ID, gene symbol, gene description, R (TXOXGH/TXOX), SD, and q (%). (PDF) [file pone.0096305.s003.pdf]

ENSEMBL ID	Unigene/Refseq	Symbol	Gene description	R		q(%)
				mean ± SD		
Up-regulated						
ENSRNOG00000010478	NM_182474.1	SPI1	Serine protease inhibitor	4,5	1,0	0,00
ENSRNOG00000024772	XM_221075	Cpsf4l	cleavage and polyadenylation specific factor 4-like	4,3	1,3	0,00
ENSRNOG00000009142	NM_198784	MUP	alpha-2u globulin (MUP 4)	3,6	1,1	0,00
ENSRNOG00000005764	NM_147213	PGCL5	alpha-2u globulin PGCL5	3,6	0,9	0,00
ENSRNOG00000012896	NM_019184	Cyp2c11	cytochrome P450 2C11 (mephenytoin 4-hydroxylase)	3,3	0,6	0,00
ENSRNOG00000025056	XM_234428	CN16B	similar to chromosome 14 open reading frame 166B	3,3	1,0	0,00
ENSRNOG00000017489	XM_226533	Gse1	genetic suppressor element 1	3,2	1,0	0,00
ENSRNOG00000008965	NM_058208	SOCS-2	suppressor of cytokine signaling 2	3,0	1,1	0,00
ENSRNOG00000013093	NM_017158	Cyp2c7	cytochrome P450, family 2, subfamily c, polypeptide 7	2,3	0,8	0,00
ENSRNOG00000025106	XM_344693	nd	similar to peroxiredoxin 1	2,2	0,6	0,00
ENSRNOG00000027784	NM_001009965	TSKU	tsukushin; similar to hepatic protein EIIH	2,1	0,6	0,00
ENSRNOG00000021354	NM_017158.2	Cyp2c7	cytochrome P450, family 2, subfamily c, polypeptide 7	2,0	0,4	0,00
ENSRNOG00000006042	Rn.198813	Rup2	urinary protein 2	1,9	0,6	0,00
ENSRNOG00000020528	NM_001007722.1	Hba2	hemoglobin, alpha 2	1,9	0,5	0,00
ENSRNOG00000013275	NM_203325.1	Mup5	major urinary protein 5 (Mup5)	1,8	0,5	0,00
ENSRNOG00000019630	NM_053493	HACL1	2-hydroxyacyl-CoA lyase 1	1,8	0,2	0,00
ENSRNOG00000008999	NM_147212.1	PGCL3	alpha-2u globulin PGCL3 (LOC259244),	1,7	0,9	0,00
ENSRNOG00000025079	NM_001025710	FAM126B	family with sequence similarity 126, member B	1,7	0,5	0,00
ENSRNOG00000028734	NM_017158	Cyp2c7	cytochrome P450, family 2, subfamily c, polypeptide 7	1,6	0,4	0,00
ENSRNOG00000008095	NM_022671	Hnf6	one cut homeobox 1 (HNF-6)	1,6	0,5	0,00
ENSRNOG00000009160	ENSRNOG00000009160	nd	novel gene	1,5	0,5	0,00
ENSRNOG00000014447	NM_012565	GCK	glucokinase	1,5	0,5	0,00
ENSRNOG00000014365	NM_053765	Gne	glucosamine (UDP-N-acetyl)-2-epimerase/N-acetylmannosamine kinase	1,5	0,2	0,00
ENSRNOG00000021310	XM_220441	Slc36A3	solute carrier family 36 (proton/amino acid symporter), member 3	1,5	0,4	0,00
ENSRNOG00000012926	NM_001000754	Olr363	olfactory receptor 363	1,5	0,6	0,00
ENSRNOG00000001979	NM_153724	RCAN1	Down syndrome critical region homolog 1 (human)	1,5	0,6	0,00
ENSRNOG00000010298	NM_001004210	HTF	X-box binding protein 1	1,4	0,1	0,00
ENSRNOG00000020529	NM_013096	nd	hemoglobin alpha-1 and alpha-2 chains	1,4	0,3	0,00
ENSRNOG00000019470	NM_032082	HAO2	hydroxyacid oxidase 2 (long chain)	1,4	0,2	0,00
ENSRNOG00000025127	NM_017177.1	Chkb	choline kinase beta (Chkb)	1,4	0,5	0,00
ENSRNOG00000013090	XM_237999	Gadd45g	growth arrest and DNA-damage-inducible, gamma	1,4	0,9	0,77
ENSRNOG00000016791	NM_017127	CHKA	choline kinase alpha	1,3	0,2	0,00
ENSRNOG00000021336	NM_001107752.2	Ttc17	tetratricopeptide repeat domain 17 (Ttc17)	1,3	0,2	0,00
ENSRNOG00000022157	XM_225266.5	Serpinb6e	serine (or cysteine) peptidase inhibitor, clade B, member 6e (Serpinb6e)	1,3	0,9	0,77
ENSRNOG00000021380	Rn.163176	FADS6	fatty acid desaturase domain family, member 6	1,3	0,2	0,00
ENSRNOG00000009474	XM_221037	DDX42	DEAD (Asp-Glu-Ala-Asp) box polypeptide 42	1,3	0,5	0,00
ENSRNOG00000017914	NM_134449	Prkcdbp	protein kinase C, delta binding protein	1,2	0,3	0,00
ENSRNOG00000010337	NM_031746	Slc13A2	solute carrier family 13 (sodium-dependent dicarboxylate transporter), member 2 (Ntcp)	1,2	0,2	0,00
ENSRNOG00000026645	XM_003751433.1	LOC100911429	uncharacterized LOC100911429 (LOC100911429)	1,2	0,3	0,00
ENSRNOG00000016122	NM_013134	HMGCR	3-hydroxy-3-methylglutaryl-Coenzyme A reductase	1,1	0,4	0,00
ENSRNOG00000007949	NM_031546	RC	regucalcin (senescence marker protein-30)	1,1	0,4	0,00
ENSRNOG00000016595	NM_024385	HHEX	hematopoietically expressed homeobox	1,1	0,3	0,00
ENSRNOG00000016044	NM_001106456.1	Mab21l3	mab-21-like 3 (C. elegans) (Mab21l3),	1,1	0,2	0,00
ENSRNOG00000020928	NM_019303	Cyp2a1	cytochrome P450 2A1 (hepatic steroid hydroxylase IIA1) gene	1,1	0,2	0,00
ENSRNOG00000005661	NM_024351.2	Hspa8	heat shock 70kDa protein 8 (Hspa8)	1,1	0,3	0,00
ENSRNOG00000020480	NM_053445	Fads1	fatty acid desaturase 1	1,1	0,7	0,77
ENSRNOG00000017962	NM_199085	SERPINB6	serine (or cysteine) peptidase inhibitor, clade B, member 6a	1,0	0,7	1,02
ENSRNOG00000010269	NM_021653	nd	type I iodothyronine deiodinase (type-I 5'deiodinase) (DIOI) (TYPE 1 DI) (5DI)	1,0	0,1	0,00
ENSRNOG00000007395	NM_017300	BAAT	bile acid CoA: amino acid N-acyltransferase (glycine N-choloyltransferase)	1,0	0,3	0,16
ENSRNOG00000019656	NM_001012047	BTD	biotinidase	1,0	0,3	0,00
ENSRNOG00000022180	XM_002725097.2	LOC100364618	hypothetical protein LOC100364618 (LOC100364618)	1,0	0,6	0,77
ENSRNOG00000013409	NM_017305	GCLM	glutamate cysteine ligase, modifier subunit	1,0	0,3	0,00
ENSRNOG00000024848	NM_001008724	FGA	fibrinogen alpha chain	1,0	0,2	0,00
ENSRNOG00000009052	XM_231739	IGF2BP3	insulin-like growth factor 2 mRNA binding protein 3	1,0	0,6	1,02
ENSRNOG00000004332	NM_031507	EGFR	epidermal growth factor receptor	0,9	0,4	0,33
ENSRNOG00000020950	Rn.162486	SYVN1	synovial apoptosis inhibitor 1, synoviolin	0,9	0,3	0,00
ENSRNOG00000025087	NM_001008808	KRT73	keratin 73	0,9	0,5	0,71
ENSRNOG00000009284	NM_012742	Hnf3a	forkhead box A1	0,9	0,3	0,00
ENSRNOG00000004329	NM_199208.1	Rdh16	retinol dehydrogenase 16 (all-trans) (Rdh16)	0,9	0,4	0,33
ENSRNOG00000009303	ENSRNOG00000009303	ZWILCH	Zwilch, kinetochore associated, homolog (Drosophila)	0,9	0,4	0,62
ENSRNOG00000007420	NM_017018	HRH1	histamine receptor H 1	0,9	0,6	1,04
ENSRNOG00000010270	NM_019622	ESPN	espin	0,9	0,3	0,00
ENSRNOG00000009274	NM_173308	FUT11	fucosyltransferase 11 (alpha (1,3) fucosyltransferase)	0,9	0,5	0,77
ENSRNOG00000025140	Rn.33031	ZFAT	zinc finger and AT hook domain containing	0,9	0,3	0,33
ENSRNOG00000004517	NM_178866	IGF1	insulin-like growth factor 1	0,9	0,4	0,52
ENSRNOG00000018420	NM_053537	OAT2	solute carrier family 22 (organic anion transporter), member 7	0,9	0,3	0,00
ENSRNOG00000026448	XM_344296	nd	similar to RIKEN cDNA 1110003E01	0,9	0,2	0,00
ENSRNOG00000009434	XM_236574	nd	similar to RIKEN cDNA 1300017J02	0,9	0,3	0,00
ENSRNOG00000025510	NM_198776	nd	beta-glo; beta-globin; beta globin minor gene	0,9	0,4	0,33
ENSRNOG00000028096	XM_347304	Marveld1	MARVEL domain containing 1	0,9	0,6	1,47
ENSRNOG00000019568	NM_138875	JUND	Jun D proto-oncogene	0,9	0,6	1,04
ENSRNOG00000014900	NM_017334	CREM	cAMP responsive element modulator	0,9	0,3	0,33
ENSRNOG00000011269	NM_031732	SULT1C3	sulfotransferase family, cytosolic, 1C, member 3	0,9	0,4	0,33
ENSRNOG00000025440	NM_001000075.1	Olr1006	olfactory receptor 1006 (Olr1006)	0,9	0,4	0,71
ENSRNOG00000015767	XM_577371.4	LOC501945	uncharacterized LOC501945 (RGD1559628)	0,9	0,3	0,00
ENSRNOG00000018225	XM_342551	Trp53inp2	tumor protein p53 inducible nuclear protein 2	0,9	0,3	0,00
ENSRNOG00000023479	XM_229609	nd	similar to RIKEN cDNA 1700001E04	0,8	0,6	1,47
ENSRNOG00000017204	Rn.17290	PQLC1	PQ-loop repeat-containing protein 1	0,8	0,1	0,00
ENSRNOG00000012189	XM_341486	LECT2	leukocyte cell-derived chemotaxin 2	0,8	0,6	1,47
ENSRNOG00000005615	NM_024127	GADD45	growth arrest and DNA-damage-inducible, alpha	0,8	0,2	0,00
ENSRNOG00000000786	NM_001002831	RPP21	ribonuclease P 21 subunit (human)	0,8	0,4	0,71
ENSRNOG00000015061	NM_053329.2	IGF-ALS	insulin-like growth factor binding protein, acid labile subunit (Igfals)	0,8	0,6	1,29
ENSRNOG00000025074	NM_012559	FGG	fibrinogen gamma chain	0,8	0,1	0,00
ENSRNOG00000008988	NM_012730	Cyp2d2	cytochrome P450 2D2	0,8	0,5	1,04

ENSRNOG00000020420	NM_012624	PKLR	pyruvate kinase, liver and RBC	0,8	0,6	1,63
ENSRNOG00000008510	NM_134403	ABTB2	ankyrin repeat and BTB (POZ) domain containing 2	0,8	0,0	0,00
ENSRNOG00000008215	XM_221124	TRIM47	tripartite motif-containing 47	0,8	0,3	0,33
ENSRNOG00000012190	XM_236535	CLDN2	claudin 2	0,8	0,3	0,33
ENSRNOG00000019262	NM_001134565.1	Trak1	trafficking protein, kinesin binding 1 (Trak1)	0,8	0,1	0,00
ENSRNOG00000022268	Rn.162959	Pnpla3	patatin-like phospholipase domain containing 3	0,8	0,8	3,38
ENSRNOG00000026316	XM_343021	WDR43	WD repeat domain 43	0,8	0,3	0,33
ENSRNOG00000000221	NM_212524	ABHD5	abhydrolase domain containing 5	0,8	0,2	0,00
ENSRNOG00000015502	XM_215693	HIAT1	hippocampus abundant gene transcript 1	0,8	0,2	0,00
ENSRNOG00000012791	NM_053503	JUB	jub, ajuba homolog (Xenopus laevis)	0,8	0,4	0,77
ENSRNOG00000016496	NM_017097	CATC	cathepsin C	0,8	0,1	0,00
ENSRNOG00000013009	NM_017025	ldhA	lactate dehydrogenase A (LDH-A) (LDH muscle subunit) (LDH-M)	0,8	0,5	1,29
ENSRNOG00000010309	NM_031553	NFYA	nuclear transcription factor-Y beta	0,8	0,0	0,00
ENSRNOG00000019681	NM_021662	Pold1	polymerase (DNA directed), delta 1, catalytic subunit	0,8	0,2	0,25
ENSRNOG00000019996	NM_012716	Slc16A1	solute carrier family 16, member 1 (monocarboxylic acid transporter 1)	0,8	0,1	0,00
ENSRNOG00000017765	XM_225548	NET1	neuroepithelial cell transforming 1	0,8	0,3	0,33
ENSRNOG00000015456	XM_344855	Zfp787	zinc finger protein 787	0,8	0,2	0,00
ENSRNOG00000019284	NM_001134565.1	Trak1	trafficking protein, kinesin binding 1 (Trak1)	0,8	0,2	0,00
ENSRNOG00000024802	NM_058209.2	Zfp37	zinc finger protein 37 (Zfp37)	0,8	0,2	0,00
ENSRNOG00000013241	XM_003748909.1	Cyp2c7	cytochrome P450 2C7-like (LOC100911552)	0,8	0,5	1,04
ENSRNOG00000028653	XM_213423	nd	similar to ribosomal protein L35a	0,8	0,2	0,33
ENSRNOG00000013267	XM_230961	PRIC285	similar to Peroxisomal proliferator-activated receptor A interacting complex 285 kDa protein (PPAR-	0,7	0,4	0,77
ENSRNOG00000012882	Rn.105436	ANKRD27	ankyrin repeat domain 27 (VPS9 domain)	0,7	0,2	0,25
ENSRNOG00000008946	NM_024139	RGD1565588	similar to calcium binding protein P22	0,7	0,2	0,33
ENSRNOG00000017558	NM_001109119.1	Tubb2a	tubulin, beta 2a	0,7	0,4	1,29
ENSRNOG00000010275	XM_342601	RGD1311940	similar to bA305P22.2.1 (novel protein, isoform 1)	0,7	0,3	0,71
ENSRNOG00000015488	XM_341908	TEAD1	TEA domain family member 1	0,7	0,2	0,33
ENSRNOG00000010264	XM_342601	nd	similar to bA305P22.2.1 (novel protein, isoform 1) (predicted)	0,7	0,3	0,71
ENSRNOG00000008209	NM_001013219	ST3GAL1	ST3 beta-galactoside alpha-2,3-sialyltransferase 1	0,7	0,4	1,04
ENSRNOG00000012913	NM_199372	Eif4a1	eukaryotic translation initiation factor 4A, isoform 1	0,7	0,2	0,33
ENSRNOG00000017309	XM_342944	nd	similar to chromosome 1 open reading frame 63	0,7	0,1	0,00
ENSRNOG00000016091	XM_237238	TMEM169	transmembrane protein 169	0,7	0,3	0,77
ENSRNOG00000011111	XM_234430	nd	similar to mKIAA1737 protein	0,7	0,4	1,47
ENSRNOG00000007758	XM_345770	HSD17B6	hydroxysteroid (17-beta) dehydrogenase 6	0,7	0,2	0,33
ENSRNOG00000026558	NM_145878	FABP	fatty acid-binding protein, (E-FABP) (cutaneous fatty acid-binding protein) (C-FABP) (DA11)	0,7	0,3	0,77
ENSRNOG00000023633	XM_236253	crabp1	cellular retinoic acid binding protein 1	0,7	0,2	0,33
ENSRNOG00000020468	XM_214592	Stard4	StAR-related lipid transfer (START) domain containing 4	0,7	0,2	0,33
ENSRNOG00000023159	XM_221123	nd	40S ribosomal protein S18 (KE-3) (KE3)	0,7	0,2	0,62
ENSRNOG00000027339	XM_342164	nd	similar to Heph protein	0,7	0,3	0,77
ENSRNOG00000009088	NM_031614	TXNRD1	thioredoxin reductase 1	0,7	0,3	0,77
ENSRNOG00000006400	NM_001012152	TBC1D14	TBC1 domain family, member 14	0,7	0,1	0,00
ENSRNOG00000020351	NM_145678	VPS4A	vacuolar protein sorting 4 homolog A (S. cerevisiae)	0,7	0,3	1,02
ENSRNOG00000014285	XM_220743	SSH2	slingshot homolog 2 (Drosophila)	0,7	0,2	0,33
ENSRNOG00000020266	NM_017245	EEF2	similar to Elongation factor 2 (EF-2); eukaryotic translation elongation factor 2	0,7	0,1	0,00
ENSRNOG00000019213	NM_022215	Gpdh	glycerol-3-phosphate dehydrogenase 1 (soluble)	0,7	0,3	0,77
ENSRNOG00000017397	NM_001012159	UPF3A	UPF3 regulator of nonsense transcripts homolog A (yeast)	0,7	0,3	0,77
ENSRNOG00000022749	XM_212981	nd	cytochrome C, somatic	0,7	0,1	0,00
ENSRNOG00000002553	XM_344241	nd	similar to RIKEN cDNA 1110003E01	0,7	0,4	1,02
ENSRNOG00000013089	NM_213626	KIF13B	kinesin 13B	0,7	0,1	0,00
ENSRNOG00000022075	NM_001114391.1	Rpl7a	ribosomal protein L7a	0,7	0,2	0,33
ENSRNOG00000022856	XM_226891.6	Dnah5	PREDICTED: Rattus norvegicus dynein, axonemal, heavy chain 5	0,7	0,1	0,25
ENSRNOG00000025160	XM_345284	TMEM56	transmembrane protein 56	0,7	0,2	0,33
ENSRNOG00000007545	NM_199115	ANGPTL4	angiopoietin-like 4	0,7	0,3	1,04
ENSRNOG00000019834	NM_001004082	Hsp90ab1	similar to Heat shock protein HSP 90-beta (HSP 84); heat shock protein 90kDa alpha (cytosolic), clas	0,7	0,2	0,33
ENSRNOG00000025063	XM_003751764.1	LOC682469	lysine-specific demethylase 3B-like (LOC682469)	0,7	0,6	2,43
ENSRNOG00000028259	ENSRNOG00000028259	nd	similar to hypothetical protein LOC199675	0,7	0,1	0,25
ENSRNOG00000019934	NM_001014148.1	Paip2	poly(A) binding protein interacting protein 2 (Paip2)	0,7	0,1	0,00
ENSRNOG00000002005	XM_573578.4	Tmprss11f	transmembrane protease, serine 11f (Tmprss11f)	0,7	0,4	1,63
ENSRNOG00000022840	NM_001166307.1	LOC498592	similar to CG32662-PA (LOC498592)	0,7	0,3	1,04
ENSRNOG00000017330	ENSRNOG00000017330	Gm9905	predicted gene 9905 [Source:MGI Symbol;Acc:MGI:3642311]	0,7	0,2	0,33
ENSRNOG00000016123	Rn.163348	Rnf144b	ring finger protein 144B	0,7	0,4	1,63
ENSRNOG00000014055	NM_053853	NAT	N-Acetyltransferase-2 (arylamine N-acetyltransferase); N-acetyltransferase 1 (arylamine N-acetyltrar	0,7	0,3	1,02
ENSRNOG00000008079	XM_214108	UGP2	UDP-glucose pyrophosphorylase 2	0,7	0,5	2,15
ENSRNOG00000015544	NM_001011995	TUBA1C	tubulin, alpha 1C	0,7	0,2	0,62
ENSRNOG00000016151	XM_237094	ANKRD23	ankyrin repeat domain 23	0,7	0,2	0,71
ENSRNOG00000025185	XM_345335	Nup188	nucleoporin 188	0,6	0,1	0,33
ENSRNOG00000025879	XM_341252	nd	similar to ribosomal protein L13	0,6	0,1	0,25
ENSRNOG00000008993	NM_133514	Mmp10	stromelysin-1 precursor (matrix metalloproteinase-3) (MMP-3) (TRANSIN-1) (SL-1) (PTR1 PROTEIN)	0,6	0,1	0,33
ENSRNOG00000024015	XM_216900	nd	similar to inner nuclear membrane protein	0,6	0,2	0,62
ENSRNOG00000014727	XM_220229	FAHD1	fumarylacetoacetate hydrolase domain containing 1	0,6	0,4	1,63
ENSRNOG00000019244	NM_001007002	MXRA8	matrix-remodelling associated 8	0,6	0,2	0,77
ENSRNOG00000004006	NM_012699	DNAJB9	DnaJ (Hsp40) homolog, subfamily B, member 9	0,6	0,3	1,02
ENSRNOG00000017437	XM_003751278.1	LOC100359583	PREDICTED: Rattus norvegicus hypothetical protein LOC100359583 (LOC100359583)	0,6	0,2	0,77
ENSRNOG00000026293	NM_021835	JUN	Jun oncogene	0,6	0,5	2,43
ENSRNOG00000017090	XM_214649	nd	similar to RIKEN cDNA 4921524J17	0,6	0,3	0,77
ENSRNOG00000015107	XM_343326	MLL2	myeloid/lymphoid or mixed-lineage leukemia 2	0,6	0,4	1,47
ENSRNOG00000024713	ENSRNOG00000024713	nd	similar to 60S ribosomal protein L7a	0,6	0,0	0,00
ENSRNOG00000010222	NM_012570	GLUD1	glutamate dehydrogenase 1	0,6	0,3	1,02
ENSRNOG00000007700	NM_022614	INHBC	inhibin beta C	0,6	0,2	0,33
ENSRNOG00000026503	XM_344923	XRRA1	X-ray radiation resistance associated 1	0,6	0,4	1,63
ENSRNOG00000020347	NM_031074.2	Nup98	nucleoporin 98kDa	0,6	0,2	0,77
ENSRNOG00000012443	NM_012930	CPT-2	carnitine palmitoyltransferase 2	0,6	0,3	1,47
ENSRNOG00000017158	XM_341907	Q5BJU2	eukaryotic translation initiation factor 4, gamma 2 (predicted)	0,6	0,3	1,02
ENSRNOG00000020601	XM_343153	HMG20B	high mobility group 20 B (predicted)	0,6	0,5	2,43
ENSRNOG00000004459	XM_343224	SDRO	orphan short-chain dehydrogenase / reductase	0,6	0,3	0,77
ENSRNOG00000011052	XM_216152	garS	glycyl-tRNA synthetase	0,6	0,2	0,77
ENSRNOG00000021033	NM_001012001	Lrrc2	leucine rich repeat containing 2 (predicted)	0,6	0,4	2,15

ENSRNOG00000028038	NM_032062	nd	huntingtin-associated protein interacting protein (duo)	0,6	0,3	1,02
ENSRNOG00000025099	XM_215047	nd	similar to hypothetical protein FLJ21816 (predicted)	0,6	0,2	0,33
ENSRNOG00000026650	Rn.81627	TMPRSS4	similar to transmembrane protease, serine 4; transmembrane protease, serine 4	0,6	0,3	1,04
ENSRNOG00000010543	NM_001034150.1	Srpr	signal recognition particle receptor ('docking protein')	0,6	0,2	0,77
ENSRNOG00000000165	NM_012621	Pfkfb01	6-phosphofructo-2-kinase/fructose-2,6-biphosphatase 1	0,6	0,3	1,29
ENSRNOG00000019968	XM_217908	TRIM8	tripartite motif-containing 8	0,6	0,1	0,00
ENSRNOG00000012141	XM_234553	Xrcc3	X-ray repair complementing defective repair in Chinese hamster cells 3 (predicted)	0,6	0,3	1,29
ENSRNOG00000007797	XM_232595	RBPSUH	recombination signal binding protein for immunoglobulin kappa J region	0,6	0,3	1,47
ENSRNOG00000016987	NM_001271281.1	Pstpip2	proline-serine-threonine phosphatase-interacting protein 2	0,6	0,2	0,77
ENSRNOG00000027618	XR_146300.1	RGD1565725	PREDICTED: Rattus norvegicus similar to hypothetical protein FLJ23834 (RGD1565725)	0,6	0,5	3,11
ENSRNOG00000014549	XM_227201	SGEF	Src homology 3 domain-containing guanine nucleotide exchange factor	0,6	0,1	0,33
ENSRNOG00000018761	XM_215080	eif3c	eukaryotic translation initiation factor 3, subunit C	0,6	0,4	2,07
ENSRNOG00000027791	XM_003751367.1	Cox7a2l2	cytochrome c oxidase, subunit 7la 2; similar to cytochrome c oxidase, subunit 7a 3	0,6	0,2	0,71
ENSRNOG00000020060	NM_172336	ATF5	activating transcription factor 5	0,6	0,4	2,15
ENSRNOG00000022138	NM_024351	Hspa8	heat shock protein 8	0,6	0,4	2,07
ENSRNOG00000009505	XM_342935	CD164L2	CD164 sialomucin-like 2	0,6	0,6	3,11
ENSRNOG00000010496	XM_217086	MED17	mediator complex subunit 17	0,6	0,2	0,77
ENSRNOG00000018131	XM_215673	Slc16A4	solute carrier family 16, member 4 (monocarboxylic acid transporter 5)	0,6	0,2	0,77
ENSRNOG00000018649	XM_230856	Slc35C2	solute carrier family 35, member C2	0,6	0,3	1,47
ENSRNOG00000006302	NM_012815	GCLC	glutamate-cysteine ligase, catalytic subunit	0,6	0,4	2,15
ENSRNOG00000027367	XM_225091	nd	similar to testicular serine protease 1	0,6	0,3	1,29
ENSRNOG00000025459	XM_216910	MARS	methionine-tRNA synthetase (predicted)	0,6	0,2	0,77
ENSRNOG00000014948	NM_138504	OSGIN1	oxidative stress induced growth inhibitor 1	0,6	0,4	2,07
ENSRNOG00000018402	NM_001085352.1	ApoC2	apolipoprotein C-II	0,6	0,2	0,77
ENSRNOG00000009754	NM_177928	PBEF1	nicotinamide phosphoribosyltransferase	0,6	0,2	0,77
ENSRNOG00000008642	NM_001012012	SNX11	sorting nexin 11	0,6	0,3	1,47
Down-regulated						
ENSRNOG00000027498	XM_221141	ENGASE	endo-beta-N-acetylglucosaminidase	-0,6	0,3	2,07
ENSRNOG00000010658	NM_017074	CTH	cystathionase (cystathionine gamma-lyase)	-0,6	0,3	1,63
ENSRNOG00000012442	XM_218855	nd	similar to transmembrane protein 2	-0,6	0,3	1,47
ENSRNOG00000016895	XM_217314	nd	similar to RIKEN cDNA 2310045B01	-0,6	0,5	4,33
ENSRNOG00000018494	NM_001012072	PPP1R3C	protein phosphatase 1, regulatory (inhibitor) subunit 3C	-0,6	0,3	2,15
ENSRNOG00000003941	NM_057114.1	Prdx1	peroxiredoxin 1 (Prdx1)	-0,6	0,1	0,62
ENSRNOG00000000386	NM_138530	PBLD	phenazine biosynthesis-like protein domain containing	-0,6	0,1	0,48
ENSRNOG00000003723	NM_001109081.1	Wdr26	WD repeat domain 26	-0,6	0,2	0,80
ENSRNOG00000008755	NM_017340	ACOX1	acyl-Coenzyme A oxidase 1, palmitoyl	-0,6	0,3	2,15
ENSRNOG00000013945	NM_178094	Itpkc	inositol 1,4,5-trisphosphate 3-kinase C	-0,6	0,1	0,48
ENSRNOG00000003677	ENSRNOG00000003677	nd	AJ18 PROTEIN	-0,6	0,4	2,76
ENSRNOG00000002210	NM_001004209	HSD17B11	hydroxysteroid (17-beta) dehydrogenase 11	-0,6	0,3	2,07
ENSRNOG00000027489	ENSRNOG00000027489	Mn1	meningioma 1	-0,6	0,4	3,11
ENSRNOG00000016484	NM_181371	GSTK1	glutathione S-transferase kappa 1	-0,6	0,3	1,47
ENSRNOG00000020457	NM_001004418	TACC2	transforming, acidic coiled-coil containing protein 2	-0,6	0,2	0,80
ENSRNOG00000013765	NM_001000689	Olr34	olfactory receptor gene Olr34	-0,6	0,3	2,07
ENSRNOG00000009740	Rn.45744	Oatp14	solute carrier organic anion transporter family, member 1c1	-0,6	0,4	2,76
ENSRNOG00000021314	NM_019238	FDFT1	farnesyl diphosphate farnesyl transferase 1	-0,6	0,4	2,76
ENSRNOG00000003443	XM_222473	Klf1	Kruppel-like factor 1 (erythroid)	-0,6	0,4	2,76
ENSRNOG00000014515	XM_227618	CDC14A	CDC14 cell division cycle 14 homolog A (S. cerevisiae)	-0,6	0,2	0,80
ENSRNOG00000027853	XM_344598	nd	histone H4	-0,6	0,2	1,02
ENSRNOG00000000312	Rn.11982	FIG4	FIG4 homolog (S. cerevisiae)	-0,6	0,4	2,76
ENSRNOG00000004302	NM_012619	Pah	phenylalanine hydroxylase	-0,6	0,3	2,07
ENSRNOG00000001396	XM_213738	MOSPD3	motile sperm domain containing 3	-0,6	0,2	1,04
ENSRNOG00000003469	NM_181089	MAST1	microtubule associated serine/threonine kinase 1	-0,6	0,3	1,47
ENSRNOG00000000701	Rn.121	ISCU	iron-sulfur cluster scaffold homolog (E. coli)	-0,6	0,3	1,63
ENSRNOG00000017593	NM_030864	MTR	5-methyltetrahydrofolate-homocysteine methyltransferase	-0,6	0,2	0,80
ENSRNOG00000016099	NM_175582	Idb4	inhibitor of DNA binding 4	-0,6	0,3	2,07
ENSRNOG00000027192	NM_001012346	nd	similar to hypothetical protein FLJ14675 (predicted)	-0,6	0,1	0,62
ENSRNOG00000016526	XM_226112	DSG2	desmoglein 2	-0,6	0,4	2,07
ENSRNOG00000013956	NM_134467	Rnf38	ring finger protein 38	-0,6	0,4	2,76
ENSRNOG00000002265	NM_016996	CASR	calcium-sensing receptor	-0,6	0,3	2,07
ENSRNOG00000023142	XM_223474	nd	similar to aldehyde dehydrogenase family 9, subfamily A1; 4-trimethylaminobutyraldehyde dehydrog	-0,6	0,1	0,48
ENSRNOG00000020950	Rn.162486	SYVN1	synovial apoptosis inhibitor 1, synoviolin	-0,6	0,2	0,71
ENSRNOG00000000414	NM_001177829.1	LOC100365935	rCG22129-like (LOC100365935)	-0,6	0,2	1,02
ENSRNOG00000017056	XM_001071565.3	LOC684681	uncharacterized LOC684681 (LOC684681)	-0,6	0,5	3,69
ENSRNOG00000013291	NM_031839	Cyp2c23	cytochrome P450 2C23	-0,6	0,4	2,07
ENSRNOG00000021200	NM_001012080	HFE2	hemochromatosis type 2 (juvenile) (human homolog)	-0,6	0,1	0,48
ENSRNOG00000014490	XM_215705	BDH2	3-hydroxybutyrate dehydrogenase, type 2	-0,6	0,1	0,48
ENSRNOG00000009928	XM_343439	BCKDHB	branched chain keto acid dehydrogenase E1, beta polypeptide	-0,6	0,2	0,78
ENSRNOG00000014739	NM_173109	Wfdc2	WAP four-disulfide core domain 2	-0,7	0,1	0,64
ENSRNOG00000002951	XM_228779	CCNB3	similar to cyclin B3	-0,7	0,5	2,76
ENSRNOG00000005479	NM_017215	Slc1A2	solute carrier family 1 (glial high affinity glutamate transporter), member 2	-0,7	0,3	1,04
ENSRNOG00000000661	XM_222245	HPS4	Hermansky-Pudlak syndrome 4 homolog (human)	-0,7	0,3	1,29
ENSRNOG00000023060	XM_227742	nd	similar to 60S RIBOSOMAL PROTEIN L29 (P23)	-0,7	0,0	0,00
ENSRNOG00000001261	NM_053567	FTCD	formiminotransferase cyclodeaminase	-0,7	0,4	2,43
ENSRNOG00000017188	NM_178847	Cyp27a1	cytochrome P450 27A1	-0,7	0,3	1,04
ENSRNOG00000020992	NM_080892	SELENBP1	selenium binding protein 1	-0,7	0,2	0,75
ENSRNOG00000014560	NM_030826.3	Gpx1	glutathione peroxidase 1 (Gpx1)	-0,7	0,4	2,07
ENSRNOG00000012391	XM_575155.5	Ttn	titin (Ttn)	-0,7	0,3	1,29
ENSRNOG00000027249	XM_223701	FANCL	Fanconi anemia, complementation group L	-0,7	0,3	1,02
ENSRNOG00000004147	XM_221100	ABCA8a	ATP-binding cassette, sub-family A (ABC1), member 8a	-0,7	0,3	1,04
ENSRNOG00000027129	NM_001109356.1	Sdr16c6	short chain dehydrogenase/reductase family 16C, member 6 (Sdr16c6)	-0,7	0,4	2,07
ENSRNOG00000013304	NM_017134	ARG1	arginase, liver	-0,7	0,4	1,47
ENSRNOG00000002013	XM_221455	SIDT1	SID1 transmembrane family, member 1	-0,7	0,1	0,48
ENSRNOG00000010922	NM_001025418	PPP2R1B	protein phosphatase 2 (formerly 2A), regulatory subunit A (PR 65), beta isoform	-0,7	0,3	1,29
ENSRNOG00000005220	XM_216738	SYNE2	spectrin repeat containing, nuclear envelope 2	-0,7	0,2	0,80
ENSRNOG00000024796	Rn.162147	LRRC47	leucine rich repeat containing 47	-0,7	0,7	4,33
ENSRNOG00000006472	NM_021863	HSPA2	heat shock protein 2	-0,7	0,5	2,43
ENSRNOG00000007478	NM_133405	CRY2	cryptochrome 2 (photolyase-like)	-0,7	0,4	1,47

ENSRNOG00000020983	XM_001078193.3	LOC690930	PREDICTED: Rattus norvegicus membrane-spanning 4-domains subfamily A member 6C-like, transc	-0,7	0,3	1,02
ENSRNOG00000023219	NM_017218	Erbb3	v-erb-b2 erythroblastic leukemia viral oncogene homolog 3 (avian)	-0,7	0,2	0,71
ENSRNOG00000016339	NM_053768	UOX	urate oxidase	-0,7	0,3	1,29
ENSRNOG00000023465	ENSRNOG00000023465	Depp	similar to hypothetical protein MGC6835	-0,7	0,6	3,69
ENSRNOG00000004644	XM_228451	nd	similar to transcription factor znf6 - human	-0,7	0,6	3,69
ENSRNOG00000016573	NM_001012345	DGAT2	diacylglycerol O-acyltransferase homolog 2 (mouse)	-0,7	0,1	0,00
ENSRNOG00000021004	ENSRNOG00000021004	RASIP1	Ras interacting protein 1	-0,7	0,3	0,80
ENSRNOG00000000933	XM_221871	nd	similar to KIAA0774 protein	-0,7	0,1	0,00
ENSRNOG00000020924	NM_001012070	Ptgdr2	G protein-coupled receptor 44 (predicted)	-0,7	0,1	0,48
ENSRNOG00000019691	NM_053425	CCS	copper chaperone for superoxide dismutase	-0,7	0,1	0,00
ENSRNOG00000000903	NM_031330	ARLY	argininosuccinate lyase	-0,7	0,2	0,48
ENSRNOG00000013704	NM_017072.1	Cps1	carbamoyl-phosphate synthetase 1, mitochondrial	-0,7	0,1	0,00
ENSRNOG00000017327	XM_344625	nd	similar to Aldo-keto reductase family 1 member C3 (Trans-1,2-dihydrobenzene-1,2-diol dehydrogena	-0,7	0,2	0,48
ENSRNOG00000019503	NM_001011916	Rec8	REC8 homolog (yeast)	-0,8	0,2	0,48
ENSRNOG00000017808	NM_053536	KLF15	Kruppel-like factor 15	-0,8	0,1	0,00
ENSRNOG00000014876	XM_237521	LPIN2	lipin 2 (predicted)	-0,8	0,2	0,62
ENSRNOG00000017673	NM_001191824.1	Spata22	spermatogenesis associated 22 (Spata22)	-0,8	0,3	0,80
ENSRNOG00000004009	NM_057155	XPNPEP2	X-prolyl aminopeptidase (aminopeptidase P) 2, membrane-bound	-0,8	0,1	0,00
ENSRNOG00000016349	NM_017084	GNMT	glycine N-methyltransferase	-0,8	0,4	1,29
ENSRNOG00000014493	XM_231193	GOLGA1	golgi autoantigen, golgin subfamily a, 1 (predicted)	-0,8	0,3	0,78
ENSRNOG00000012929	XM_220736	wsb1	WD repeat and SOCS box-containing 1	-0,8	0,8	4,33
ENSRNOG00000000302	XM_215423	sesn1	sestrin 1	-0,8	0,2	0,48
ENSRNOG00000022292	NM_001014171.1	Veph1	ventricular zone expressed PH domain-containing 1 (Veph1)	-0,8	0,2	0,48
ENSRNOG00000006789	NM_001109986.1	Ddit3	DNA-damage inducible transcript 3 (Ddit3), transcript variant 1	-0,8	0,3	0,62
ENSRNOG00000004679	XM_229979	fign	fidgetin	-0,8	0,3	0,78
ENSRNOG00000008498	XM_216288	GABARAPL1	GABA(A) receptor-associated protein like 1	-0,8	0,3	0,71
ENSRNOG00000009438	NM_133617	SERPINA6	serine (or cysteine) peptidase inhibitor, clade A, member 6	-0,8	0,3	0,97
ENSRNOG00000006653	NM_130748	Slc38A4	solute carrier family 38, member 4	-0,8	0,3	0,48
ENSRNOG00000028199	NM_001001101	Olr1530	olfactory receptor (fragmente)	-0,8	0,8	4,33
ENSRNOG00000001496	XM_341791	nd	similar to Alcohol sulfotransferase (Hydroxysteroid sulfotransferase) (ST) (ST-60)	-0,8	0,7	2,76
ENSRNOG00000003370	NM_013078	OTC	ornithine carbamoyltransferase	-0,8	0,3	0,48
ENSRNOG00000016807	NM_022521	OAT	ornithine aminotransferase (gyrate atrophy)	-0,8	0,4	1,02
ENSRNOG00000013484	NM_031509	GSTA3	glutathione S-transferase A3	-0,8	0,3	0,77
ENSRNOG00000012123	NM_017126	FDX1	ferredoxin 1	-0,9	0,2	0,00
ENSRNOG00000001827	NM_022257	MASP1	mannan-binding lectin serine peptidase 1	-0,9	0,3	0,48
ENSRNOG00000012811	Rn.44189	SPINT1	serine peptidase inhibitor, Kunitz type 1	-0,9	0,2	0,00
ENSRNOG00000005905	NM_017031	PDE4B	phosphodiesterase 4B, cAMP specific	-0,9	0,4	0,78
ENSRNOG00000017672	NM_138547	Akr1c14	aldo-keto reductase family 1, member C14	-0,9	0,2	0,00
ENSRNOG00000020928	NM_019303	Cyp2a1	cytochrome P450 IIA1 (hepatic steroid hydroxylase IIA1) gene	-0,9	0,4	0,59
ENSRNOG00000000961	XM_222147	GLT1D1	similar to RIKEN cDNA 5730455A04 gene (predicted)	-0,9	0,3	0,48
ENSRNOG00000001983	XM_223299	nd	UDP-glucuronosyltransferase 2B3 precursor, microsomal (UDPGT) (testosterone, DHT, beta-estradi	-0,9	0,3	0,48
ENSRNOG00000028616	NM_198780	PEPCK	phosphoenolpyruvate carboxykinase 1 (soluble)	-0,9	0,9	3,69
ENSRNOG00000027313	NM_001013181	Zbtb16	zinc finger protein 145	-0,9	0,4	0,78
ENSRNOG00000015660	NM_031350	pex3	peroxisomal biogenesis factor 3	-1,0	0,3	0,48
ENSRNOG00000007886	NM_053288	ORM	orosomucoid 1	-1,0	0,4	0,71
ENSRNOG00000019924	NM_172075	THOP1	thimet oligopeptidase 1	-1,0	0,7	2,07
ENSRNOG00000008781	NM_001106749.1	RGD1310769	similar to HSPC288 (RGD1310769)	-1,0	0,1	0,00
ENSRNOG00000011612	NM_022403	TDO2	tryptophan 2,3-dioxygenase	-1,0	0,1	0,00
ENSRNOG00000011351	NM_012860	AdoMet	methionine adenosyltransferase I, alpha	-1,0	0,3	0,48
ENSRNOG00000016841	XM_226351	ALT2	glutamic pyruvate transaminase (alanine aminotransferase) 2	-1,0	0,2	0,00
ENSRNOG00000013728	XM_221047	POLG2	polymerase (DNA directed), gamma 2, accessory subunit	-1,0	0,1	0,00
ENSRNOG00000005341	XM_229938	UPP2	uridine phosphorylase 2	-1,0	0,3	0,00
ENSRNOG00000012464	NM_019286	Adh1	alcohol dehydrogenase 1 (class I)	-1,0	0,6	1,51
ENSRNOG00000002597	NM_173305	Hsd17b6	hydroxysteroid (17-beta) dehydrogenase 6	-1,0	0,4	0,48
ENSRNOG00000011200	NM_030850	BHMT	betaine-homocysteine methyltransferase	-1,0	0,7	1,63
ENSRNOG00000004821	XM_235312	SNTB1	syntrophin, basic 1	-1,1	0,3	0,00
ENSRNOG00000013302	NM_001009920	GST YC2	glutathione S-transferase YC-2 (chain 2) (GST YC2)	-1,1	0,4	0,48
ENSRNOG00000017236	XM_225538	3-alpha-Hsd3	similar to Aldo-keto reductase family 1 member C2 (Trans-1,2-dihydrobenzene-1,2-diol dehydrogena	-1,1	0,8	1,47
ENSRNOG00000011016	Rn.48707	Slc7A2	solute carrier family 7 (cationic amino acid transporter, y+ system), member 2	-1,1	0,3	0,24
ENSRNOG00000006893	XM_231833	PPM1K	protein phosphatase 1K (PP2C domain containing)	-1,2	0,5	0,48
ENSRNOG00000001388	NM_053962	sdh	serine dehydratase	-1,2	0,8	1,15
ENSRNOG00000014726	Rn.162462	ACSM4	acyl-CoA synthetase medium-chain family member 4	-1,2	0,3	0,00
ENSRNOG00000015740	XM_341581	RNF125	ring finger protein 125	-1,2	0,3	0,00
ENSRNOG00000000768	NM_053299	UBD	ubiquitin D	-1,2	0,4	0,00
ENSRNOG00000014990	NM_012695.3	Sult2a6	sulfotransferase family 2A, dehydroepiandrosterone (DHEA)-preferring, member 6 (Sult2a6)	-1,2	0,6	0,48
ENSRNOG00000019982	XM_214838	ETHE1	ethylmalonic encephalopathy 1	-1,3	0,8	1,02
ENSRNOG00000026722	NM_001025131.1	Sult2a2	sulfotransferase family 2A, dehydroepiandrosterone (DHEA)-preferring, member 2 (Sult2a2)	-1,3	0,6	0,48
ENSRNOG00000008837	NM_013157	Ass1	argininosuccinate synthetase 1	-1,3	0,4	0,00
ENSRNOG00000012787	XM_346288	Tmem164	transmembrane protein 164	-1,3	0,5	0,00
ENSRNOG00000017619	NM_022407	Aldh	aldehyde dehydrogenase 1 family, member A1	-1,4	0,4	0,24
ENSRNOG00000019481	NM_031241	Cyp8b1	cytochrome P450, family 8, subfamily b, polypeptide 1	-1,4	0,3	0,00
ENSRNOG00000013464	NM_012674.2	Spink3	serine peptidase inhibitor, Kazal type 3 (Spink3)	-1,4	0,5	0,00
ENSRNOG00000007507	XM_231524	Aass	aminoadipate-semialdehyde synthase (Aass), nuclear gene encoding mitochondrial protein	-1,5	0,1	0,00
ENSRNOG00000013410	NM_152936.1	Spink	serine peptidase inhibitor, Kazal type 1 (Spink1)	-1,6	0,6	0,00

Table S3. Hepatic genes regulated by GH in hypothyroid-orchidectomized rats. The administrations of vehicle (TXOX) or GH (TXOXGH) to TXOX rats were described in Material and Methods. Then, differently expressed genes in the livers were identified by DNA microarrays. The analysis is based on the SAM statistical technique and differentially-expressed genes were discovered using a FDR less than 5% and a mean ratio of log2 > |0.58|. The table shows ENSEMBL gene ID, gene symbol, gene description, R (TXOXGH/TXOX), SD, and q(%).
