# Supplementary material for: Lipid Profiling and Transcriptomic Analysis Reveals a Functional Interplay between Estradiol and Growth Hormone in Liver
Source: PLoS One. 2014 May 9;9(5):e96305. doi: 10.1371/journal.pone.0096305 (PMC4015979; doi:10.1371/journal.pone.0096305)
Supplement: Table S4 — Hepatic genes regulated by GH in E2-treated hypothyroid-orchidectomized rats. The administrations of vehicle (TXOX) or E2 plus GH (TXOXE2GH) in TXOX rats were described in Material and Methods. Then, differently expressed genes in the livers were identified by DNA microarrays. The analysis is based on the SAM statistical technique and differentially-expressed genes were discovered using a FDR less than 5% and a mean ratio of log2>|0.58|. The table shows ENSEMBL gene ID, gene symbol, gene description, R (TXOXE2GH/TXOX), SD, and q (%). (PDF) [file pone.0096305.s004.pdf]

ENSEMBL ID	Unigene	Symbol	GENE NAME	R mean ± SD	q(%)
**Up-regulated**					
ENSRNOG00000010478	NM_182474.1	LOC299282	Serine protease inhibitor (LOC299282)	0,7	0,00
ENSRNOG00000010410	NM_012657.2	Serpina3k	serine (or cysteine) peptidase inhibitor, clade A, member 3K	0,5	0,00
ENSRNOG00000024745	XM_003754035.1	Mup	major urinary protein-like (LOC688514)	1,4	0,00
ENSRNOG00000017562	NM_001033958.2	Obp3	alpha-2u globulin PGCL4 (Obp3), transcript variant 2	1,5	0,00
ENSRNOG00000013093	NM_017158	Cyp2c7	cytochrome P450, family 2, subfamily c, polypeptide 7	0,6	0,00
ENSRNOG00000024772	XM_001081632.1	Cpsf4l	cleavage and polyadenylation specific factor 4-like	0,7	0,00
ENSRNOG00000005764	NM_147213.1	PGCL5	alpha-2u globulin PGCL5 (LOC259245)	1,0	0,00
ENSRNOG00000009142	XM_003749949.1	PGCL4	alpha-2u globulin PGCL4	1,0	0,00
ENSRNOG00000021354	NM_017158.2	Cyp2c7	cytochrome P450, family 2, subfamily c, polypeptide 7	0,8	0,00
ENSRNOG00000025056	NM_001037783.2	LOC314328	similar to chromosome 14 open reading frame 166B (LOC314328)	0,4	0,00
ENSRNOG00000017489	XM_001078877.3	Gse1	genetic suppressor element 1	0,4	0,00
ENSRNOG00000028734	NM_017158	Cyp2c7	cytochrome P450, family 2, subfamily c, polypeptide 7	0,6	0,00
ENSRNOG00000008095	NM_022671.2	ONECUT1	one cut homeobox 1 (Onecut1)	0,8	0,00
ENSRNOG00000014116	ENSRNOG00000014116	ST-20	alcohol sulfotransferase (hydroxysteroid sulfotransferase) (ST) (ST-20)	0,6	0,00
ENSRNOG00000021310	NM_001024764.1	Slc36a3	solute carrier family 36 (proton/amino acid symporter), member 3	0,7	0,00
ENSRNOG00000008965	NM_058208	SOCS2	suppressor of cytokine signaling 2 (SOCS2)	1,2	0,00
ENSRNOG00000008999	NM_147212	PGCL3	alpha-2u globulin PGCL3	0,8	0,00
ENSRNOG00000021336	XM_230313	nd	tetratricopeptide repeat domain 17 (predicted)	0,6	0,00
ENSRNOG00000027784	NM_001009965	TSKU	tsukushin; similar to hepatic protein EIIH	0,7	0,00
ENSRNOG00000019630	NM_053493	Hacl1	2-hydroxyacyl-CoA lyase 1	0,3	0,00
ENSRNOG00000021380	XM_221105	FADS6	fatty acid desaturase domain family, member 6	0,7	0,00
ENSRNOG00000025106	XM_344693	LOC502175	similar to peroxiredoxin 1	0,5	0,00
ENSRNOG00000006042	ENSRNOG00000006042	Rup2	urinary protein 2	0,6	0,00
ENSRNOG00000012896	NM_019184.2	Cyp2c11	cytochrome P450, subfamily 2, polypeptide 11	0,9	1,22
ENSRNOG00000012443	NM_012930.1	Cpt2	carnitine palmitoyltransferase 2 (Cpt2), nuclear gene encoding mitochondrial protei	0,7	0,00
ENSRNOG00000014365	NM_053765	gne	glucosamine (UDP-N-acetyl)-2-epimerase/N-acetylmannosamine kinase	0,5	0,00
ENSRNOG00000019656	NM_001012047	BTD	biotinidase	0,3	0,00
ENSRNOG00000025079	XM_237184	FAM126B	family with sequence similarity 126, member B	0,6	0,31
ENSRNOG00000007545	NM_199115	ANGPTL4	angiopoietin-like 4	0,4	0,00
ENSRNOG00000019681	NM_021662	Pold1	polymerase (DNA directed), delta 1, catalytic subunit	0,2	0,00
ENSRNOG00000014447	NM_012565	gck	glucokinase	0,7	1,22
ENSRNOG00000009875	NM_053781	Akr1b7	aldo-keto reductase family 1, member B7	0,7	1,22
ENSRNOG00000003020	NM_001001509	Hdmcp	mitochondrial hepatocellular carcinoma-downregulated carrier protein	0,8	1,74
ENSRNOG00000009160	ENSRNOG00000009160	RGD1562494	similar to keratin 6 alpha	0,2	0,00
ENSRNOG00000017914	NM_134449	PRKCDBP	protein kinase C, delta binding protein	0,5	0,31
ENSRNOG00000015483	NM_019340	nd	regulator of G-protein signaling 3 (RGS3) (fragment)	0,3	0,00
ENSRNOG00000017210	XM_341519	SLC22A23	solute carrier family 22, member 23	0,2	0,00
ENSRNOG00000000786	NM_001002831	rpp21	ribonuclease P 21 subunit (human)	0,1	0,00
ENSRNOG00000013275	NM_203325.1	Mup5	major urinary protein 5	0,5	0,98
ENSRNOG00000005661	NM_024351.2	Hspa8	heat shock 70kDa protein 8 (Hspa8)	0,7	1,74
ENSRNOG00000013241	NM_017158	Cyp2c7	Cytochrome P450 2C7 (CYPIIC7) (P450F) (PTF1)	0,6	1,74
ENSRNOG00000008215	XM_221124	TRIM47	tripartite motif-containing 47	0,3	0,00
ENSRNOG00000020990	NM_130752	FGF21	fibroblast growth factor 21	0,7	1,99
ENSRNOG00000009754	NM_177928.3	Nampt	nicotinamide phosphoribosyltransferase	0,2	0,00
ENSRNOG00000025127	NM_017177.1	Chkb	choline kinase beta (Chkb)	0,4	0,55
ENSRNOG00000016924	NM_016987	ACLY	ATP citrate lyase	0,6	2,18
ENSRNOG00000008946	NM_024139	RGD1565588	similar to calcium binding protein P22	0,2	0,00
ENSRNOG00000010337	NM_031746	SLC13A2	solute carrier family 13 (sodium-dependent dicarboxylate transporter), member 2	0,1	0,00
ENSRNOG00000010298	NM_001004210	Xbp1	X-box binding protein 1	0,1	0,00
ENSRNOG00000004006	NM_012699	dnajb9	DnaJ (Hsp40) homolog, subfamily B, member 9	0,5	1,69
ENSRNOG00000028096	XM_347304	Marveld1	MARVEL domain containing 1	0,4	1,21
ENSRNOG00000001979	NM_153724	RCAN1	Down syndrome critical region homolog 1 (human)	0,5	1,91
ENSRNOG00000007060	NM_001007144	Adfp	adipose differentiation related protein	0,9	3,05
ENSRNOG00000010224	XM_218916	RAB30	RAB30, member RAS oncogene family	0,7	2,52
ENSRNOG00000016122	NM_013134	HMGCR	3-hydroxy-3-methylglutaryl-Coenzyme A reductase	0,8	2,52
ENSRNOG00000009284	NM_012742	foxa1	forkhead box A1	0,4	1,22
ENSRNOG00000011078	NM_053464	SRM	spermidine synthase	0,6	1,99
ENSRNOG00000007240	XM_232622	rrs1	RRS1 ribosome biogenesis regulator homolog (S. cerevisiae)	0,3	1,21
ENSRNOG00000019284	NM_001134565.1	Trak1	trafficking protein, kinesin binding 1 (Trak1)	0,6	1,99
ENSRNOG00000016044	XM_227522	RGD1308694	similar to hypothetical protein MGC47256	0,2	0,00
ENSRNOG00000004500	NM_012603	Myc	myelocytomatosis oncogene	0,8	3,05

ENSRNOG00000014201	XM_236614	ARMET	arginine-rich, mutated in early stage tumors	0,2	0,00
ENSRNOG00000025063	XM_003751764.1	LOC682469	lysine-specific demethylase 3B-like (LOC682469)	0,5	1,99
ENSRNOG00000015061	NM_053329	Igfals	insulin-like growth factor binding protein, acid labile subunit	0,5	1,74
ENSRNOG00000010222	NM_012570	glud1	glutamate dehydrogenase 1	0,2	0,31
ENSRNOG00000019262	XM_236715	RGD1307844	similar to 106 kDa O-GlcNAc transferase-interacting protein	0,4	1,56
ENSRNOG00000013963	NM_001008725	IL6ST	interleukin 6 signal transducer	0,2	0,55
ENSRNOG00000015236	NM_031668	mybbp1a	MYB binding protein (P160) 1a	0,6	2,28
ENSRNOG00000010321	NM_001024986.1	RGD1306186	similar to RIKEN cDNA 4930569K13 (RGD1306186)	0,2	0,31
ENSRNOG00000024802	NM_058209.2	Zfp37	zinc finger protein 37 (Zfp37)	0,1	0,00
ENSRNOG00000020608	NM_001011980	ppan	peter pan homolog (Drosophila)	0,1	0,00
ENSRNOG00000018168	NM_001009601	KLC4	kinesin light chain 4	0,4	1,74
ENSRNOG00000000165	NM_012621	pfkfb1	6-phosphofructo-2-kinase/fructose-2,6-biphosphatase 1	0,3	1,60
ENSRNOG00000012117	NM_001009478	nd	serum amyloid A 4 (predicted)	0,4	2,01
ENSRNOG00000016791	NM_017127	CHKA	choline kinase alpha	0,7	3,05
ENSRNOG00000018704	NM_022869	NOLC1	nucleolar and coiled-body phosphoprotein 1	0,5	2,08
ENSRNOG00000009432	NM_019356	EIF2S1	hypothetical gene supported by NM_019356; eukaryotic translation initiation factor	0,3	1,56
ENSRNOG00000009474	XM_221037	DDX42	DEAD (Asp-Glu-Ala-Asp) box polypeptide 42	0,2	0,55
ENSRNOG00000023633	XM_236253	CRABP1	cellular retinoic acid binding protein 1	0,4	1,99
ENSRNOG00000025087	NM_001008808	KRT73	keratin 73	0,4	1,99
ENSRNOG00000003620	NM_053433	fmo3	flavin containing monooxygenase 3	0,4	1,74
ENSRNOG00000009335	NM_001007797	nd	arrestin domain containing 3	0,3	1,49
ENSRNOG00000017640	NM_024139	Chp	calcium binding protein p22; similar to calcium binding protein P22	0,2	0,55
ENSRNOG00000019996	NM_012716	SLC16A1	solute carrier family 16, member 1 (monocarboxylic acid transporter 1)	0,5	2,08
ENSRNOG00000004100	XM_343250	TRIB1	tribbles homolog 1 (Drosophila)	0,1	0,00
ENSRNOG00000009052	XM_231739	igf2bp3	insulin-like growth factor 2 mRNA binding protein 3	0,5	2,08
ENSRNOG00000020420	NM_012624	PKLR	pyruvate kinase, liver and RBC	0,5	2,28
ENSRNOG00000024848	NM_001008724.1	Fga	fibrinogen alpha chain (Fga), transcript variant 1	0,4	1,99
ENSRNOG00000019834	NM_001004082	HSP90AB1	similar to heat shock protein 1, beta; similar to Heat shock protein HSP 90-beta (HSI	0,2	1,21
ENSRNOG00000012189	XM_341486	Lect2	leukocyte cell-derived chemotaxin 2	0,2	1,21
ENSRNOG00000007949	NM_031546	rgn	regucalcin (senescence marker protein-30)	0,4	2,08
ENSRNOG00000017397	NM_001012159	upf3a	UPF3 regulator of nonsense transcripts homolog A (yeast)	0,3	1,74
ENSRNOG00000007128	XM_342517	Nol5a	nucleolar protein 5A; similar to nucleolar protein 5A; similar to Nol5a	0,5	2,28
ENSRNOG00000010247	XM_230493	nd	similar to mKIAA1300 protein (predicted)	0,2	0,98
ENSRNOG00000022529	XM_341519	nd	ion transporter protein	0,2	0,55
ENSRNOG00000011552	XM_226493	MON1B	similar to hypothetical protein 5031407H10 (predicted)	0,1	0,00
ENSRNOG00000009274	NM_173308	FUT11	fucosyltransferase 11 (alpha (1,3) fucosyltransferase)	0,5	2,28
ENSRNOG00000023463	XM_221404	PARP9	poly (ADP-ribose) polymerase family, member 9	0,4	1,74
ENSRNOG00000022138	NM_024351	nd	heat shock protein 8	0,3	1,74
ENSRNOG00000026984	XM_220957	nd	similar to CCT (chaperonin containing TCP-1) zeta subunit	0,4	2,08
ENSRNOG00000015502	XM_215693	Hiat1	hippocampus abundant gene transcript 1	0,2	1,22
ENSRNOG00000009565	NM_053551	PDK4	pyruvate dehydrogenase kinase, isozyme 4	0,4	1,99
ENSRNOG00000020928	NM_019303	Cyp2a1	cytochrome P450 2A1 (hepatic steroid hydroxylase IIA1) gene	0,3	0,99
ENSRNOG00000019682	NM_145781	Timm13	translocase of inner mitochondrial membrane 13 homolog (yeast)	0,2	1,22
ENSRNOG00000010309	NM_031553	NFYB	nuclear transcription factor-Y beta	0,2	1,48
ENSRNOG00000022786	XM_345525	nd	similar to ribosomal protein S12; 40S ribosomal protein S12	0,3	1,74
ENSRNOG00000011052	XM_216152	Gars	glycyl-tRNA synthetase	0,2	1,22
ENSRNOG00000016390	XM_214451	EEF1E1	eukaryotic translation elongation factor 1 epsilon 1	0,2	1,49
ENSRNOG00000026645	XM_003751433.1	LOC100911429	uncharacterized LOC100911429 (LOC100911429)	0,4	2,08
ENSRNOG00000014055	NM_053853	NAT	N-Acetyltransferase-2 (arylamine N-acetyltransferase); N-acetyltransferase 1	0,0	0,00
ENSRNOG00000014254	NM_031559	Cpt1a	carnitine palmitoyltransferase 1a, liver	0,5	3,05
ENSRNOG00000016123	XM_344576	Rnf144b	ring finger protein 144B	0,1	0,00
ENSRNOG00000019358	NM_012689	Esr1	estrogen receptor 1	0,4	2,28
ENSRNOG00000014242	NM_001002286	Mrgprc	MAS-related G-protein coupled receptor, member C	0,3	1,99
ENSRNOG00000016987	NM_001271281.1	Pstpip2	proline-serine-threonine phosphatase-interacting protein 2 (Pstpip2)	0,1	0,55
ENSRNOG00000017120	XM_214979	ABHD2	abhydrolase domain containing 2	0,3	1,99
ENSRNOG00000010198	XM_234398	nd	cytosolic acyl CoA thioester hydrolase, inducible (Long chain acyl-CoA thioester hy	0,6	4,17
ENSRNOG00000010270	NM_019622	Espn	espin	0,1	0,98
ENSRNOG00000010543	NM_001034150.1	Srpr	signal recognition particle receptor subunit alpha	0,4	2,28
ENSRNOG00000004786	NM_019623	Cyp4f1	cytochrome P450 4F1	0,2	1,69
ENSRNOG00000017147	NM_001024791.1	Epn3	epsin 3 (Epn3)	0,4	2,28
ENSRNOG00000025312	NM_001001364	nd	olfactory receptor gene Olr1051	0,3	2,08
ENSRNOG00000020922	NM_138887	hspb6	heat shock protein, alpha-crystallin-related, B6	0,4	2,08
ENSRNOG00000012141	XM_234553	xrcc3	X-ray repair complementing defective repair in Chinese hamster cells 3	0,4	3,05
ENSRNOG00000017330	ENSRNOG00000017330	Gm9905	predicted gene 9905	0,2	1,22
ENSRNOG00000008411	XM_342531	SEC23B	Sec23 homolog B (S. cerevisiae)	0,3	2,08

ENSRNOG00000015767	XM_577371.4	LOC501945	uncharacterized LOC501945 (RGD1559628)	0,5	3,65
ENSRNOG00000021364	XM_002724803.2	LOC691311	hypothetical protein LOC691311 (LOC691311)	0,5	3,65
ENSRNOG00000000221	NM_212524	ABHD5	abhydrolase domain containing 5	0,2	1,49
ENSRNOG00000027644	NM_001109325.1	Krtap13-2	keratin associated protein 13-2 (Krtap13-2), nuclear gene encoding mitochondrial p	0,2	1,74
ENSRNOG00000017309	XM_342944	RGD1359529	similar to chromosome 1 open reading frame 63	0,2	1,74
ENSRNOG00000008250	XR_146028.1	LOC100363637	nucleophosmin 1-like (LOC100363637)	0,5	3,65
ENSRNOG00000021273	XM_345438	CRLS1	cardiolipin synthase 1	0,2	1,56
ENSRNOG00000025111	XM_218484	NFKBID	nuclear factor of kappa light polypeptide gene enhancer in B-cells inhibitor, delta	0,3	2,28
ENSRNOG00000023159	XM_221123	Rp18	similar to ribosomal protein S18	0,2	1,69
ENSRNOG00000007219	NM_175761	HSP90AA1	similar to heat shock protein 1, alpha; heat shock protein 90, alpha (cytosolic), clas	0,5	3,65
ENSRNOG00000025140	NM_001134957.1	Zfat	zinc finger and AT hook domain containing	0,3	2,08
ENSRNOG00000023303	NM_001011928	DPEP2	dipeptidase 2	0,4	2,52
ENSRNOG00000010264	XM_342601	nd	similar to bA305P22.2.1 (novel protein, isoform 1) (predicted)	0,5	3,65
ENSRNOG00000014160	NM_012670	TCP1	t-complex 1	0,5	4,17
ENSRNOG00000001466	XM_217906	LOC687842	Cytochrome P450 2C24 (CYPIIC24) (P450-PROS2)	0,2	1,56
ENSRNOG00000014812	XM_218181	Nalp12	NACHT, leucine rich repeat and PYD containing 12	0,5	3,65
ENSRNOG00000016595	NM_024385	hhex	hematopoietically expressed homeobox	0,5	3,65
ENSRNOG00000022509	XM_236813	LOC301165	similar to hypothetical protein 4932415M13	0,4	2,52
ENSRNOG00000004517	NM_178866	Igf1	insulin-like growth factor 1	0,4	3,35
ENSRNOG00000009857	NM_001012014	Ftsj3	FtsJ homolog 3 (E. coli)	0,5	3,65
ENSRNOG00000011384	XM_342344	AIMP1	small inducible cytokine subfamily E, member 1	0,3	2,08
ENSRNOG00000011421	XM_216529	SMAP2	small ArfGAP2	0,1	1,49
ENSRNOG00000022396	NM_001128065.1	Rpl36a	ribosomal protein L36a (Rpl36a)	0,3	1,99
ENSRNOG00000018453	XM_235295	nop2	nucleolar protein 1; similar to nucleolar protein 1	0,3	2,08
ENSRNOG00000012913	NM_199372.2	Eif4a1	eukaryotic translation initiation factor 4A1 (Eif4a1)	0,3	2,08
ENSRNOG00000016217	NM_053689.2	Gtpbp4	GTP binding protein 4 (Gtpbp4)	0,4	3,05
Down-regulated					
ENSRNOG00000008239	NM_001005893	REPIN1	replication initiator 1	0,4	2,28
ENSRNOG00000017133	XM_225174	LOC306766	hypothetical LOC306766	0,2	1,82
ENSRNOG00000000459	NM_012708	psmb9	proteasome (prosome, macropain) subunit, beta type 9 (large multifunctional peptic	0,4	2,52
ENSRNOG00000013484	XM_347371	GSTA3	glutathione S-transferase A3	0,5	3,05
ENSRNOG00000000127	XM_342926	KPNA6	karyopherin alpha 6 (importin alpha 7); similar to karyopherin (importin) alpha 6	0,4	2,52
ENSRNOG00000013659	XM_221043	tex2	testis expressed 2	0,3	2,08
ENSRNOG00000006396	NM_013026	sdc1	syndecan 1	0,3	1,82
ENSRNOG00000014779	NM_022265	PDCD4	programmed cell death 4	0,3	2,08
ENSRNOG00000007639	XM_233250	C8B	complement component 8, beta polypeptide	0,5	3,05
ENSRNOG00000005612	NM_019287.2	Apob	apolipoprotein B (Apob)	0,2	1,82
ENSRNOG00000014886	NM_001025034.1	fam107b	family with sequence similarity 107, member B	0,3	2,08
ENSRNOG00000003253	NM_022390	QDPR	quinoid dihydropteridine reductase	0,2	1,49
ENSRNOG00000016205	XM_237327	Rnf11l	ring finger protein 11-like	0,4	2,52
ENSRNOG00000022074	XM_345342.4	C5	similar to Complement C5 precursor (Hemolytic complement)	0,5	3,05
ENSRNOG00000016099	NM_175582	id4	inhibitor of DNA binding 4	0,2	1,82
ENSRNOG00000009404	NM_001127712.1	Bco2	beta-carotene oxygenase 2 (Bco2)	0,3	2,52
ENSRNOG00000004405	NM_012723	PIGR	polymeric immunoglobulin receptor	0,5	3,05
ENSRNOG00000005437	NM_031714	Hrsp12	heat-responsive protein 12	0,4	2,08
ENSRNOG00000025685	XM_003753726.1	Pkp4	plakophilin 4, transcript variant 2 (Pkp4)	0,3	1,82
ENSRNOG00000002052	NM_022543.2	Ccdc80	coiled-coil domain containing 80 (Ccdc80)	0,2	1,82
ENSRNOG00000002610	NM_152790	carhsp1	calcium regulated heat stable protein 1	0,3	1,82
ENSRNOG00000003709	NM_021593	kmo	kynurenine 3-monooxygenase (kynurenine 3-hydroxylase)	0,3	1,82
ENSRNOG00000017381	NM_019369.2	Itih4	inter-alpha-inhibitor H4 heavy chain	0,4	2,08
ENSRNOG00000002136	NM_001128311.1	Abcg3	ATP-binding cassette, subfamily G (WHITE), member 3 (Abcg3)	0,2	1,56
ENSRNOG00000002280	NM_001173339.1	Sh3bgrl	SH3 domain binding glutamic acid-rich protein like	0,2	1,49
ENSRNOG00000011630	NM_017135	AK3L1	adenylate kinase 3-like 1	0,0	0,00
ENSRNOG00000020684	XM_213484	vat1	vesicle amine transport protein 1 homolog (T californica)	0,4	2,08
ENSRNOG00000018553	NM_001008369	PITPNM1	phosphatidylinositol transfer protein, membrane-associated 1	0,3	1,82
ENSRNOG00000017799	ENSRNOG00000017799	LOC685895	similar to Thymidine kinase, cytosolic	0,2	1,56
ENSRNOG00000014571	NM_001047111.2	DBNDD2	dysbindin (dystrobrevin binding protein 1) domain containing 2	0,4	2,28
ENSRNOG00000011419	NM_031057	Aldh6a1	aldehyde dehydrogenase 6 family, member A1	0,5	2,52
ENSRNOG00000000304	NM_031812	CD164	CD164 molecule, sialomucin	0,2	1,49
ENSRNOG00000019556	XM_342760	CD9	CD9 molecule	0,6	4,17
ENSRNOG00000005195	NM_012837	Cst3	cystatin C	0,3	1,82
ENSRNOG00000005447	XM_346895	LOC688105	hypothetical protein LOC688105; LOC360590	0,4	2,08
ENSRNOG00000024170	NM_138888	phf5a	PHD finger protein 5A	0,6	4,17
ENSRNOG00000014652	NM_019158	AQP8	aquaporin 8	0,2	1,49

ENSRNOG00000007697	XM_233251	C8A	complement component 8, alpha polypeptide	0,3	1,82
ENSRNOG00000014129	NM_001106355.1	Hoga1	4-hydroxy-2-oxoglutarate aldolase 1 (Hoga1), nuclear gene encoding mitochondrial	0,3	1,82
ENSRNOG00000001921	NM_001013864.1	Lpp	LIM domain containing preferred translocation partner in lipoma (Lpp)	0,3	1,82
ENSRNOG00000003941	NM_057114.1	Prdx1	peroxiredoxin 1 (Prdx1)	0,4	1,99
ENSRNOG00000009488	NM_012942	Cyp7a1	cytochrome P450, family 7, subfamily a, polypeptide 1	0,6	3,65
ENSRNOG00000014504	NM_013123	Il1r1	interleukin 1 receptor, type I	0,4	2,28
ENSRNOG00000020947	NM_001004083	EGLN2	EGL nine homolog 2 (C. elegans)	0,5	3,05
ENSRNOG00000017056	NM_001106397.1	LOC684681	microtubule associated monoxygenase, calponin and LIM domain containing 1 (Mic	0,4	2,08
ENSRNOG00000014515	XM_227618	CDC14A	CDC14 cell division cycle 14 homolog A (S. cerevisiae)	0,3	1,99
ENSRNOG00000027129	NM_001109356.1	Sdr16c6	short chain dehydrogenase/reductase family 16C, member 6 (Sdr16c6)	0,1	1,21
ENSRNOG00000015491	NM_001013152	RCL1	RNA terminal phosphate cyclase-like 1	0,2	1,49
ENSRNOG00000004725	NM_012552	CELA1	chymotrypsin-like elastase family, member 1	0,3	1,82
ENSRNOG00000001229	XM_241632	COL18A1	collagen, type XVIII, alpha 1	0,3	1,82
ENSRNOG00000022360	NM_001177819.1	LOC100362110	LRRGT00155-like (LOC100362110)	0,6	4,17
ENSRNOG00000021366	NM_001166679.1	Tas2r116	Taste receptor, type 2, member 116 (Tas2r116)	0,3	1,82
ENSRNOG00000016526	XM_226112	DSG2	desmoglein 2	0,1	1,21
ENSRNOG00000010389	NM_133583	Ndrg2	N-myc downstream regulated gene 2	0,4	1,99
ENSRNOG00000000981	NM_031541	SCARB1	scavenger receptor class B, member 1	0,4	2,08
ENSRNOG00000004062	NM_012516	C4BPA	complement component 4 binding protein, alpha	0,3	1,82
ENSRNOG00000000028	NM_053347	NDE1	nudE nuclear distribution gene E homolog 1 (A. nidulans)	0,4	2,08
ENSRNOG00000017123	NM_012512	B2M	beta-2 microglobulin	0,6	3,65
ENSRNOG00000000068	XM_232413	PPP2R5A	protein phosphatase 2, regulatory subunit B', alpha isoform	0,0	0,00
ENSRNOG00000016955	XM_214513	fam188a	similar to RIKEN cDNA 2310047O13	0,2	1,52
ENSRNOG00000016691	XM_345167	RGD1306809	similar to hypothetical protein FLJ30596	0,2	1,56
ENSRNOG00000010633	NM_012820	Acsl1	acyl-CoA synthetase long-chain family member 1	0,3	1,69
ENSRNOG00000002304	XM_001079369.3	Tnfrsf13b	tumor necrosis factor receptor superfamily, member 13b	0,2	1,22
ENSRNOG00000011913	NM_012532	CP	ceruloplasmin	0,4	1,82
ENSRNOG00000003326	XM_222526	RRN3	RRN3 RNA polymerase I transcription factor homolog (S. cerevisiae)	0,4	1,99
ENSRNOG00000012440	NM_053307	msrA	methionine sulfoxide reductase A	0,1	0,31
ENSRNOG00000021200	NM_001012080	hfe2	hemochromatosis type 2 (juvenile) (human homolog)	0,4	2,08
ENSRNOG00000001980	NM_001004271	Ugt2b36	UDP glucuronosyltransferase 2 family, polypeptide B36	0,1	0,55
ENSRNOG00000000936	NM_012748	stx2	syntaxin 2	0,3	1,82
ENSRNOG00000009440	NM_012770	GUCY1B2	guanylate cyclase 1, soluble, beta 2	0,1	0,00
ENSRNOG00000002208	XM_223229	SHROOM3	shroom family member 3	0,1	0,98
ENSRNOG00000008374	XM_341249	NIPSNAP1	4-nitrophenylphosphatase domain and non-neuronal SNAP25-like protein homolog	0,3	1,69
ENSRNOG00000008781	XM_216756	RGD1310769	similar to HSPC288	0,4	2,28
ENSRNOG00000014806	NM_001134751.1	Pnkd	paroxysmal nonkinesigenic dyskinesia (Pnkd), nuclear gene encoding mitochondri	0,4	1,99
ENSRNOG00000003662	NM_024381	GK	glycerokinase (GK)	0,1	0,55
ENSRNOG00000005669	NM_001009662	Car8	carbonic anhydrase 8	0,3	1,56
ENSRNOG00000017305	NM_017248.1	Hnrnpa1	nuclear ribonucleoprotein A1	0,5	2,52
ENSRNOG00000004502	NM_001271212.1	hal	autophagy related 13	0,2	1,60
ENSRNOG00000012216	NM_053802.1	tgfbi	transforming growth factor, beta induced	0,2	1,56
ENSRNOG00000003515	NM_012844.3	ephX1	epoxide hydrolase 1, microsomal, transcript variant 2,	0,5	2,04
ENSRNOG00000018899	XM_345342	C5	complement component 5	0,4	1,76
ENSRNOG00000025555	NM_001012359	nd	angiogenin ribonuclease 2	0,3	1,82
ENSRNOG00000027520	XM_343776	PLS3	plastin 3 (T-isoform)	0,3	1,69
ENSRNOG00000023708	NM_001039008.1	Tmem176a	transmembrane protein 176A	0,1	0,55
ENSRNOG00000002240	NM_001012017	DIRC2	disrupted in renal carcinoma 2 (human)	0,1	0,31
ENSRNOG00000025460	NM_001271215.1	TMEM71	transmembrane protein 71	0,3	1,82
ENSRNOG00000005487	NM_001007750	CHPT1	choline phosphotransferase 1	0,3	1,69
ENSRNOG00000014540	XM_001075984.2	RGD1309621	similar to hypothetical protein FLJ10652 (RGD1309621)	0,5	2,28
ENSRNOG00000013291	NM_031839	Cyp2c23	cytochrome P450 2C23	0,3	1,69
ENSRNOG00000000025	NM_131906	nd	solute carrier family 21 member 5 (sodium-independent organic anion transporting	0,4	1,99
ENSRNOG00000019691	NM_053425	Ccs	copper chaperone for superoxide dismutase	0,2	1,22
ENSRNOG00000017777	NM_017201	ahcY	similar to Adenosylhomocysteinase (S-adenosyl-L-homocysteine hydrolase) (AdoH	0,1	0,98
ENSRNOG00000027466	NM_001024335.1	CD27	CD27 molecule	0,5	2,52
ENSRNOG00000004284	NM_017258	btg1	B-cell translocation gene 1, anti-proliferative	0,4	1,82
ENSRNOG00000020992	NM_080892	SELENBP1	selenium binding protein 1	0,5	2,28
ENSRNOG00000016967	NM_053301	HFE	hemochromatosis	0,2	1,21
ENSRNOG00000013302	NM_001009920	nd	glutathione S-tranasferase YC-2 (chain 2) (GST YC2) (GST class-alpha)	0,4	1,82
ENSRNOG00000007947	XM_342702	Fam13a1	family with sequence similarity 13, member A1	0,2	1,21
ENSRNOG00000012971	NM_130409	nd	Complement factor H-related protein	0,3	1,56
ENSRNOG00000020950	NM_001100739.1	SYVN1	synovial apoptosis inhibitor 1, synoviolin	0,4	1,82
ENSRNOG00000012475	NM_001004242	prr5	proline rich 5 (renal)	0,2	1,22
ENSRNOG00000016349	NM_017084	Gnmt	glycine N-methyltransferase	0,4	1,99

ENSRNOG00000012442	XM_218855	RGD1305254	similar to transmembrane protein 2	0,1	0,00
ENSRNOG00000002289	NM_020081	ildr1	immunoglobulin-like domain containing receptor 1	0,2	1,21
ENSRNOG00000014876	XM_237521	LPIN2	lipin 2	0,6	3,65
ENSRNOG00000003307	XM_344744	gcdH	glutaryl-Coenzyme A dehydrogenase	0,5	2,28
ENSRNOG00000023060	XM_227742	RpL29	60S ribosomal protein L29-like (LOC295487)	0,3	1,49
ENSRNOG00000019832	XM_219691	Bbs1	Bardet-Biedl syndrome 1 homolog (human)	0,3	1,69
ENSRNOG00000019982	XM_214838	Ethe1	ethylmalonic encephalopathy 1	0,7	3,65
ENSRNOG00000019466	XM_231089	AGPAT2	1-acylglycerol-3-phosphate O-acyltransferase 2 (lysophosphatidic acid acyltransfer	0,3	1,56
ENSRNOG00000027846	XM_225391	Hist1h4b	histone cluster 1, H4b (Hist1h4b)	0,5	2,28
ENSRNOG00000008498	XM_216288	GABARAPL1	GABA(A) receptor-associated protein like 1	0,3	1,49
ENSRNOG00000015032	XM_001059222.3	TEX13B	testis expressed 13B	0,4	1,82
ENSRNOG00000002210	NM_001004209	HSD17B11	hydroxysteroid (17-beta) dehydrogenase 11	0,3	1,49
ENSRNOG00000002039	NM_001271410.1	Evi5	ecotropic viral integration site 5	0,4	1,82
ENSRNOG00000004125	NM_016995	C4BPB	complement component 4 binding protein, beta	0,4	1,82
ENSRNOG00000018074	NM_133295.3	Ces1d	carboxylesterase 1D	0,7	3,65
ENSRNOG00000018967	XM_002728606.2	LOC690751	WD repeat-containing protein 59-like (LOC690751)	0,4	1,82
ENSRNOG00000011971	NM_138900	C1s	complement component 1, s subcomponent	0,4	1,82
ENSRNOG00000016271	NM_001006963	itm2b	integral membrane protein 2B	0,1	0,00
ENSRNOG00000019532	NM_001004221	MGC93975	similar to 2310044H10Rik protein	0,6	2,52
ENSRNOG00000015020	NM_031510	IDH1	isocitrate dehydrogenase 1 (NADP+), soluble	0,3	1,49
ENSRNOG00000005599	NM_023103	A1i3	alpha-1-inhibitor III	0,5	2,28
ENSRNOG00000024777	NM_175760	nd	cytochrome P450, family 4, subfamily a, polypeptide 14	0,6	3,05
ENSRNOG00000013704	NM_017072.1	Cps1	carbamoyl-phosphate synthetase 1 (Cps1), nuclear gene encoding mitochondrial pı	0,2	0,98
ENSRNOG00000000961	XM_001073476.3	Glt1d1	glycosyltransferase 1 domain containing 1	0,1	0,00
ENSRNOG00000009582	XM_003749692.1	RPE65	uncharacterized LOC100911535	0,3	1,56
ENSRNOG00000018542	XM_342960	nd	taste receptor, type 1, member 2	0,5	2,08
ENSRNOG00000002981	NM_031601	CACNA1G	calcium channel, voltage-dependent, T type, alpha 1G subunit	0,4	1,69
ENSRNOG00000015774	NM_001192002.1	Mreg	melanoregulin	0,4	1,82
ENSRNOG00000018740	NM_001039549.1	Ugt1a	UDP glucuronosyltransferase 1 family, polypeptide A5	0,3	1,63
ENSRNOG00000014560	NM_030826.3	Gpx1	glutathione peroxidase 1	0,5	2,08
ENSRNOG00000023491	NM_001013185.1	Adck3	aarF domain containing kinase 3 (Adck3), nuclear gene encoding mitochondrial pro	0,4	1,82
ENSRNOG00000000445	NM_031504.3	C4a	complement component 4A (Rodgers blood group) (C4a),	0,4	1,69
ENSRNOG00000001834	XM_213563	Fam128b	family with sequence similarity 128, member B	0,6	2,52
ENSRNOG00000019503	NM_001011916	Rec8	REC8 homolog (yeast)	0,2	1,21
ENSRNOG00000015660	NM_031350	pex3	peroxisomal biogenesis factor 3	0,2	0,00
ENSRNOG00000005906	NM_031561	FAT/CD36	fatty acid translocase/CD36 (thrombospondin receptor)	0,4	1,82
ENSRNOG00000004009	NM_057155	XPNPEP2	X-prolyl aminopeptidase (aminopeptidase P) 2, membrane-bound	0,4	1,69
ENSRNOG00000000302	XM_215423	sesn1	sestrin 1	0,4	1,69
ENSRNOG00000019094	NM_177426.1	Gstm2	Glutathione S-transferase Mu 2	0,3	1,22
ENSRNOG00000014493	XM_231193	Golga1	golgi autoantigen, golgin subfamily a, 1	0,4	1,82
ENSRNOG00000003370	NM_013078	OTC	ornithine carbamoyltransferase	0,4	1,69
ENSRNOG00000016737	NM_001130077.1	Tcerg1l	transcription elongation regulator 1-like	0,7	3,05
ENSRNOG00000000469	NM_001008841.2	RT1-CE3	RT1 class I, locus CE3 (RT1-CE3)	0,4	1,82
ENSRNOG00000004821	XM_235312	SNTB1	syntrophin, basic 1	0,2	1,21
ENSRNOG00000006653	NM_130748	Slc38a4	solute carrier family 38, member 4	0,1	0,00
ENSRNOG00000018267	NM_001012134	b3gnt7	UDP-GlcNAc:betaGal beta-1,3-N-acetylglucosaminyltransferase 7	0,5	1,82
ENSRNOG00000023599	NM_024157	Cfi	complement factor I	0,3	1,49
ENSRNOG00000012464	NM_019286	adh1	alcohol dehydrogenase 1 (class I)	0,6	2,35
ENSRNOG00000023142	XM_223474	nd	similar to aldehyde dehydrogenase family 9, subfamily A1; 4-trimethylaminobutyral	0,3	1,21
ENSRNOG00000005905	NM_017031	Pde4b	phosphodiesterase 4B, cAMP specific	0,2	0,31
ENSRNOG00000017808	NM_053536	klf15	Kruppel-like factor 15	0,3	1,21
ENSRNOG00000014700	NM_001005546	Ttc36	tetratricopeptide repeat domain 36	0,3	1,21
ENSRNOG00000012391	XM_575155.5	Ttn	titin (Ttn)	0,2	0,55
ENSRNOG00000005159	XM_001072719.3	RGD1308133	similar to RIKEN cDNA 1700010C24 (RGD1308133)	0,9	4,17
ENSRNOG00000001806	NM_053348	FetuB	fetuin B	0,4	1,69
ENSRNOG00000016386	NM_031975	PTMS	parathymosin	0,4	1,56
ENSRNOG00000021004	NM_001106261.2	Rasip1	Ras interacting protein 1 (Rasip1)	0,6	2,08
ENSRNOG00000011200	NM_030850	bhmT	betaine-homocysteine methyltransferase	0,7	2,28
ENSRNOG00000005220	XM_216738	SYNE2	spectrin repeat containing, nuclear envelope 2	0,4	1,69
ENSRNOG00000020776	NM_022389	DHCR7	7-dehydrocholesterol reductase	0,6	2,08
ENSRNOG00000018522	NM_078623	Echs1	enoyl Coenzyme A hydratase, short chain, 1, mitochondrial	0,5	1,82
ENSRNOG00000001338	NM_017233	hpd	4-hydroxyphenylpyruvate dioxygenase	0,3	1,21
ENSRNOG00000023587	XM_341557	DHTKD1	dehydrogenase E1 and transketolase domain containing 1	0,3	0,98
ENSRNOG00000007793	NM_173322	PNRC1	proline-rich nuclear receptor coactivator 1	0,4	1,49
ENSRNOG00000014739	NM_173109	WFDC2	WAP four-disulfide core domain 2	0,1	0,74

ENSRNOG00000021338	NM_178021	TMEM132A	transmembrane protein 132A	0,5	1,69
ENSRNOG00000007507	XM_231524	nd	aminoadipate-semialdehyde synthase (predicted)	0,8	2,52
ENSRNOG00000002783	NM_001012027	serpinc1	serine (or cysteine) peptidase inhibitor, clade C (antithrombin), member 1	0,6	1,99
ENSRNOG00000001344	NM_032416	ALDH2	aldehyde dehydrogenase 2 family (mitochondrial)	0,7	2,28
ENSRNOG00000006472	NM_021863	Hspa2	heat shock protein 2	0,9	3,65
ENSRNOG00000008465	NM_134390	TMEM176B	transmembrane protein 176B	0,1	0,00
ENSRNOG00000002207	XM_223381	Guf1	GUF1 GTPase homolog (S. cerevisiae)	0,5	1,69
ENSRNOG00000005861	NM_017080	HSD11B1	hydroxysteroid 11-beta dehydrogenase 1	0,4	1,49
ENSRNOG00000018494	NM_001012072	Ppp1r3c	protein phosphatase 1, regulatory (inhibitor) subunit 3C	0,7	2,08
ENSRNOG00000016460	NM_053021	clu	clusterin	0,5	1,69
ENSRNOG00000010714	NM_172010	FGL1	fibrinogen-like 1	0,6	1,82
ENSRNOG00000003443	XM_222473	Klf1	Kruppel-like factor 1 (erythroid)	0,8	2,52
ENSRNOG00000008638	NM_001025065.1	Angpt 3	angiopoietin-like 3	0,6	1,82
ENSRNOG00000000453	NM_001004084	nd	class II MHC RT1.D(N) beta chain precursor	0,3	0,91
ENSRNOG00000020983	XM_001078193.3	LOC690930	membrane-spanning 4-domains subfamily A member 6C-like, transcript variant 1 (L	0,5	1,69
ENSRNOG00000008364	NM_012520	cat	catalase	0,2	0,00
ENSRNOG00000006859	XR_146922.1	LOC100909424	uncharacterized LOC100909424 (LOC100909424)	0,5	1,56
ENSRNOG00000008837	NM_013157	ASS1	argininosuccinate synthetase 1	0,5	1,69
ENSRNOG00000017891	NM_134380	nd	integral membrane transport protein UST5R	0,8	2,52
ENSRNOG00000004147	XM_221100	Abca8a	ATP-binding cassette, sub-family A (ABC1), member 8a	0,2	0,31
ENSRNOG00000011351	NM_012860	mat1a	methionine adenosyltransferase I, alpha	0,2	0,00
ENSRNOG00000007457	NM_199093	SERPING1	serine (or cysteine) peptidase inhibitor, clade G, member 1	0,6	1,82
ENSRNOG00000012123	NM_017126	FDX1	ferredoxin 1	0,4	1,49
ENSRNOG00000012929	XM_220736	WSB1	WD repeat and SOCS box-containing 1	0,6	1,82
ENSRNOG00000005604	NM_019287	APOB	apolipoprotein B	0,4	1,49
ENSRNOG00000001827	NM_022257	masp1	mannan-binding lectin serine peptidase 1	0,2	0,00
ENSRNOG00000006893	XM_231833	Ppm1k	protein phosphatase 1K (PP2C domain containing)	0,2	0,00
ENSRNOG00000004302	NM_012619	pah	phenylalanine hydroxylase	0,5	1,69
ENSRNOG00000013736	NM_057146	C9	complement component 9	0,2	0,00
ENSRNOG00000001983	XM_223299	Ugt2b10	UDP glucuronosyltransferase 2 family, polypeptide B10 (Ugt2b10)	0,2	0,00
ENSRNOG00000020817	NM_012693	Cyp2a2	cytochrome P450, subfamily 2A, polypeptide 1	0,3	0,00
ENSRNOG00000021314	NM_019238	FDFT1	farnesyl diphosphate farnesyl transferase 1	0,5	1,56
ENSRNOG00000000443	NM_031504.3	C4a	complement component 4A (Rodgers blood group) (C4a)	0,9	2,05
ENSRNOG00000013956	NM_134467	Rnf38	ring finger protein 38	0,4	0,98
ENSRNOG00000012787	XM_346288	TMEM164	transmembrane protein 164	0,6	1,69
ENSRNOG00000027445	NM_022392.1	Insig1	insulin induced gene 1 (Insig1)	0,8	1,82
ENSRNOG00000001856	NM_001109612.1	Rps4y2	ribosomal protein S4, Y-linked 2 (Rps4y2)	0,6	1,69
ENSRNOG00000005341	XM_229938	UPP2	uridine phosphorylase 2	0,6	1,69
ENSRNOG00000028661	XM_230900	CTCFL	CCCTC-binding factor (zinc finger protein)-like	0,7	1,82
ENSRNOG00000002237	XM_340994	nd	similar to Protein disulfide isomerase-related	0,6	1,56
ENSRNOG00000009301	NM_001010921.1	Gsta5	glutathione S-transferase alpha 5 (Gsta5)	0,6	1,56
ENSRNOG00000018937	NM_031154	Gstm7	glutathione S-transferase mu 3; glutathione S-transferase mu 4; glutathione S-trans	0,2	0,00
ENSRNOG00000020928	NM_019303	Cyp2a1	cytochrome P450 IIA1 (hepatic steroid hydroxylase IIA1) gene	0,6	1,45
ENSRNOG00000013279	NM_031841	Scd2	stearoyl-coenzyme A desaturase 4 (Scd2)	0,8	1,82
ENSRNOG00000013765	NM_001000689	nd	olfactory receptor gene Olr34	0,6	1,49
ENSRNOG00000001990	NM_173295	Ugt2b1	UDP glucuronosyltransferase 2 family, polypeptide B17	0,5	1,22
ENSRNOG00000028638	XM_221655	Il10rb	interleukin 10 receptor, beta	0,6	1,49
ENSRNOG00000000978	NM_013105	nd	cytochrome P450, subfamily 3A, polypeptide 3	0,8	1,94
ENSRNOG00000011612	NM_022403	tdo2	tryptophan 2,3-dioxygenase	0,3	0,00
ENSRNOG00000016841	XM_226351	GPT2	glutamic pyruvate transaminase (alanine aminotransferase) 2	0,2	0,00
ENSRNOG00000010658	NM_017074	CTH	cystathionase (cystathionine gamma-lyase)	0,6	1,22
ENSRNOG00000014726	XM_219392	ACSM4	acyl-CoA synthetase medium-chain family member 4	0,5	0,55
ENSRNOG00000019481	NM_031241	Cyp8b1	cytochrome P450, family 8, subfamily b, polypeptide 1	0,3	0,00
ENSRNOG00000017619	NM_022407	aldh1a1	aldehyde dehydrogenase 1 family, member A1	0,4	0,61
ENSRNOG00000018735	NM_001134510.1	RGD1561161	similar to BC067074 protein (RGD1561161)	0,4	0,00
ENSRNOG00000011016	NM_001134686.2	Slc7a2	solute carrier family 7 (cationic amino acid transporter, y+ system), member 2 (Slc7a	0,6	0,98
ENSRNOG00000016807	NM_022521	OAT	ornithine aminotransferase (gyrate atrophy)	0,4	0,00
ENSRNOG00000018237	NM_138974	GSTP1	glutathione-S-transferase, pi 1	0,7	1,21
ENSRNOG00000009921	XM_216782	serpina3m	serine (or cysteine) proteinase inhibitor, clade A, member 3M	1,2	2,08
ENSRNOG00000028616	NM_198780	PCK1	phosphoenolpyruvate carboxykinase 1 (soluble)	0,7	0,98
ENSRNOG00000001831	NM_001107896.1	Ppfibp1	PTPRF interacting protein, binding protein 1 (liprin beta 1) (Ppfibp1)	0,9	1,49
ENSRNOG00000012458	NM_031543	CYP2E1	cytochrome P450 2E1	0,5	0,00
ENSRNOG00000015740	XM_341581	RNF125	ring finger protein 125	0,2	0,00
ENSRNOG00000001809	NM_133428	HRG	histidine-rich glycoprotein; similar to histidine-rich glycoprotein	0,3	0,00
ENSRNOG00000008371	NM_013144.1	IGFBP1	insulin-like growth factor binding protein 1 (Igfbp1)	1,4	2,28

**ENSRNOG00000018764**	**XM_224719**	**B3gnt3**	**UDP-GlcNAc:betaGal beta-1,3-N-acetylglucosaminyltransferase 3**	**0,8**	**0,98**
**ENSRNOG00000009438**	**NM_133617.2**	**Serpina10**	**serpin peptidase inhibitor, clade A (alpha-1 antiproteinase, antitrypsin), member 10**	**0,2**	**0,00**
**ENSRNOG00000013464**	**NM_012674.2**	**Spink3**	**serine peptidase inhibitor, Kazal type 3 (Spink3)**	**0,6**	**0,00**
**ENSRNOG00000001957**	**NM_001007718**	**SULT1E1**	**sulfotransferase family 1E, estrogen-preferring, member 1; estrogen sulfotransferas**	**0,7**	**0,00**
**ENSRNOG00000000768**	**NM_053299**	**UBD**	**ubiquitin D**	**1,8**	**2,08**
**ENSRNOG00000013410**	**NM_152936.1**	**Spink1**	**serine peptidase inhibitor, Kazal type 1 (Spink1)**	**0,6**	**0,00**
**ENSRNOG00000010079**	**NM_019292**	**CAR3**	**carbonic anhydrase 3**	**0,7**	**0,00**

Table S4. Hepatic genes regulated by GH in E2-treated hypothyroid-orchidectomized rats. The administrations of vehicle (TXOX) or E2 plus GH (TXOXE2GH) in TXOX rats were described in Material and Methods. Then, differently expressed genes in the livers were identified by DNA microarrays. The analysis is based on the SAM statistical technique and differentially-expressed genes were discovered using a FDR less than 5% and a mean ratio of log2 > |0.58|. The table shows ENSEMBL gene ID, gene symbol, gene description, R (TXOXE2GH/TXOX), SD, and q(%).
