# Supplementary material for: Lipid Profiling and Transcriptomic Analysis Reveals a Functional Interplay between Estradiol and Growth Hormone in Liver
Source: PLoS One. 2014 May 9;9(5):e96305. doi: 10.1371/journal.pone.0096305 (PMC4015979; doi:10.1371/journal.pone.0096305)
Supplement: Table S5 — Hepatic genes regulated by GH whose mean expression values are different from those in E2- and E2 plus GH-treated hypothyroid-orchidectomized rats. Hormonal replacements with GH (TXOXGH), E2 (TXOXE2) or E2 plus GH (TXOXE2GH) in TXOX rats were described in Material and Methods. Then, differently expressed genes in the livers were identified by DNA microarrays. SAM multiclass analysis was performed to identify GH regulated genes (TXOXGH) whose mean expression values were significantly different from E2 (TXOXE2)- or E2 plus GH (TXOXE2GH)-treated TXOX rats. The table shows ENSEMBL ID, Unigene/Refseq, gene symbol, gene description, R (mean expression), and q (%). (PDF) [file pone.0096305.s005.pdf]

ENSMBL	RefSeq/Unigene	Symbol	Gene description	R			q(%)
				GH	E2	E2-GH	
ENSRNOG00000001056	NM_001013121	Snapc2	small nuclear RNA activating complex, polypeptide 2	9,9	-16,7	9,3	0,0
ENSRNOG00000006400	NP_001012152.1	TBC1D14	TBC1 domain family, member 14	9,8	-3,3	-4,1	0,0
ENSRNOG00000023830	NM_138539	Dnase2a	deoxyribonuclease II alpha	8,9	-2,8	-3,8	1,0
ENSRNOG00000022707	NM_001109514.1	Pmp2	peripheral myelin protein 2	8,6	-2,8	-3,7	1,0
ENSRNOG00000014313	ENSRNOG00000014313	Myb	similar to Myb proto-oncogene protein (C-myb)	8,5	-2,7	-3,7	1,6
ENSRNOG00000026497	NM_001012207.1	Pigc	phosphatidylinositol glycan anchor biosynthesis, class C	8,4	-2,8	-3,5	0,6
ENSRNOG00000024809	NP_001020190.1	mettl11a	methyltransferase like 11A	7,4	-2,2	-3,3	4,5
ENSRNOG00000017883	XP_216007.3	CAMSAP1	calmodulin regulated spectrin-associated protein 1	6,8	-2,1	-3,1	5,1
ENSRNOG00000017967	XP_341552.2	ASB13	ankyrin repeat and SOCS box-containing 13	6,8	-2,1	-3,0	5,1
ENSRNOG00000014549	XP_227201.3	SGEF	Src homology 3 domain-containing guanine nucleotide exchange factor	6,5	-2,2	-2,8	0,6
ENSRNOG00000013441	NM_012767	GNRH1	gonadotropin-releasing hormone 1 (luteinizing-releasing hormone)	6,4	-2,1	-2,7	0,6
ENSRNOG00000024025	NP_001028240.1	EDC4	enhancer of mRNA decapping 4	6,2	-2,0	-2,6	1,7
ENSRNOG00000002985	NM_138922	nos1ap	nitric oxide synthase 1 (neuronal) adaptor protein	6,1	-1,8	-2,7	5,1
ENSRNOG00000025944	ENSRNOG00000025944	RB26	Ras-related protein Rab-26	5,8	-1,8	-2,5	2,6
ENSRNOG00000028544	XP_221795.1	Dppa1-ps1	developmental pluripotency associated 1, pseudogene 1	5,7	-1,9	-2,4	0,6
ENSRNOG00000021270	XP_230548.3	TRMT6	similar to CGI-09 protein; tRNA methyltransferase 6 homolog (S. cerevisiae)	5,4	-1,8	-2,3	1,4
ENSRNOG00000018662	NM_012816	AMACR	alpha-methylacyl-CoA racemase	5,4	-1,9	-2,1	0,0
ENSRNOG00000020817	NM_012693	Cyp2a1	cytochrome P450 2A1 (hepatic steroid hydroxylase IIA1)	5,0	-2,5	-1,2	0,0
ENSRNOG00000021233	XP_230604.1	itpa	inosine triphosphatase (nucleoside triphosphate pyrophosphatase)	5,0	-1,6	-2,1	1,7
ENSRNOG00000019470	NM_032082	hao2	hydroxyacid oxidase 2 (long chain)	4,6	-4,1	0,6	0,0
ENSRNOG00000013736	NM_057146	C9	complement component 9	4,5	-2,0	-1,4	0,0
ENSRNOG00000004549	NP_001005873.1	DHX40	DEAH (Asp-Glu-Ala-His) box polypeptide 40	4,5	5,2	-8,6	0,6
ENSRNOG00000010298	Q9R1S4	Xbp1	X-box binding protein 1	4,5	-3,8	0,5	0,0
ENSRNOG00000007036	NM_012775	tgfbr1	transforming growth factor, beta receptor 1	4,5	-1,4	-1,9	2,6
ENSRNOG00000010337	NM_031746.1	Slc13A2	solute carrier family 13 (sodium-dependent dicarboxylate transporter), member 2	4,4	-4,7	1,4	0,0
ENSRNOG00000026974	NP_001014178.1	Dbndd1	dysbindin (dystrobrevin binding protein 1) domain containing 1	4,3	-1,4	-1,8	2,1
ENSRNOG00000024990	XP_575157.1	Ppp1r1c	protein phosphatase 1, regulatory (inhibitor) subunit 1C	4,2	-1,4	-1,8	2,1
ENSRNOG00000009505	XP_342936.2	CD164L2	CD164 sialomucin-like 2	4,2	-1,4	-1,7	0,6
ENSRNOG00000003566	NM_001009626	Apoh	apolipoprotein H (beta-2-glycoprotein I) (Apoh)	4,1	-6,9	3,8	0,6
ENSRNOG00000020950	NM_001100739.1	Syvn1	synovial apoptosis inhibitor 1, synoviolin	4,1	-0,2	-2,9	0,0
ENSRNOG00000016151	XP_237094.2	Ankrd23	ankyrin repeat domain 23	4,0	-1,3	-1,7	1,4
ENSRNOG00000018952	Q5CCT7_RAT	SEMA3G	sema domain, immunoglobulin domain (Ig), short basic domain, secreted, (semaphorin) 3G	3,9	-1,3	-1,7	2,9
ENSRNOG00000010275	XP_342602.2	RGD1311940	similar to bA305P22.2.1 (novel protein, isoform 1)	3,7	-1,2	-1,5	1,0
ENSRNOG00000007395	NP_058996.1	baat	bile acid Co A: amino acid N-acyltransferase (glycine N-choloyltransferase)	3,7	-1,3	-1,5	0,0
ENSRNOG00000004014	XP_237816.2	CHMP6	chromatin modifying protein 6	3,7	-2,8	0,1	4,5
ENSRNOG00000011382	XP_226076.2	WDR33	WD repeat domain 33	3,6	-1,1	-1,5	4,5
ENSRNOG00000002304	XM_001079369.3	Tnfrsf13b	tumor necrosis factor receptor superfamily, member 13B	3,6	-3,1	0,4	0,0
ENSRNOG00000013570	XP_343472.1	RAD54L2	Rad54 like 2 (S. cerevisiae)	3,5	-2,6	0,0	1,4
ENSRNOG00000026503	XP_344924.2	XRRA1	X-ray radiation resistance associated 1	3,4	-1,2	-1,4	1,4
ENSRNOG00000010602	NM_021752	birc2	baculoviral IAP repeat-containing 2	3,4	-1,1	-1,4	1,7
ENSRNOG00000001957	NM_012883	SULT1E1	sulfotransferase family 1E (estrogen)	3,4	-3,2	0,7	0,0
ENSRNOG00000009303	ENSRNOG00000009303	Zwilch	Zwilch, kinetochore associated, homolog (Drosophila)	3,4	-1,2	-1,4	0,6
ENSRNOG00000012896	NM_019184	Cyp2c11	Cytochrome P450 2C11, male-specific (CYPIIC11)	3,3	-2,8	0,3	0,0

ENSRNOG00000015004	ENSRNOG00000015004	nd	similar to RAD54B homolog isoform 1	3,3	-1,1	-1,4	3,8
ENSRNOG00000011630	NM_017135	AK3L1	adenylate kinase 3-like 1	3,3	-1,1	-1,3	1,7
ENSRNOG00000002425	NM_017225	PCTP	phosphatidylcholine transfer protein	3,2	3,8	-6,2	1,6
ENSRNOG00000025625	NM_020082	Rnase4	ribonuclease, RNase A family 4	3,2	-1,9	-0,5	1,7
ENSRNOG00000014611	NM_017228	ATN1	atrophin 1	3,2	-1,9	-0,5	5,1
ENSRNOG00000016496	NM_017097.1	Ctsc	cathepsin C	3,2	-0,6	-1,7	1,6
ENSRNOG00000006925	XP_575458.1	Zfp786	Zfp786	3,1	-5,3	2,9	1,6
ENSRNOG00000001060	NP_001014149.1	U1snrnpbp	U11/U12 snRNP 35K	3,1	-5,3	3,0	0,0
ENSRNOG00000008642	XP_573185.1	Snx11	sorting nexin 11	3,0	-1,0	-1,3	2,6
ENSRNOG00000018712	ENSRNOG00000018712	CAMK2A	Calcium/calmodulin-dependent protein kinase type II alpha chain	2,9	3,3	-5,5	1,4
ENSRNOG00000022402	NM_030830	LUZP1	leucine zipper protein 1	2,8	-0,9	-1,2	5,1
ENSRNOG00000009160	NP_001008828.1	RGD1562494	similar to keratin 6 alpha	2,8	-2,7	0,6	0,0
ENSRNOG00000012146	NM_001047114.1	Pomt2	protein-O-mannosyltransferase 2; serine palmitoyltransferase, long chain base subunit 2	2,8	-4,7	2,6	0,6
ENSRNOG00000026116	XP_344709.2	sall3	sal-like 3 (Drosophila)	2,8	-4,7	2,6	0,0
ENSRNOG00000000304	NM_031812	CD164	CD164 molecule, sialomucin	2,7	-0,8	-1,3	2,1
ENSRNOG00000020773	NP_001013168.1	Map3k11	mitogen-activated protein kinase kinase kinase 11	2,7	-3,1	1,0	3,8
ENSRNOG00000001736	NM_053995	BDH1	3-hydroxybutyrate dehydrogenase, type 1	2,7	-1,4	-0,7	4,5
ENSRNOG00000011980	NM_057142	lrrc7	leucine rich repeat containing 7	2,6	-1,0	-0,9	3,8
ENSRNOG00000013323	NM_001106204.1	Rnf217	probable E3 ubiquitin-protein ligase RNF217	2,6	-3,0	1,0	1,0
ENSRNOG00000009474	XP_221037.2	DDX42	DEAD (Asp-Glu-Ala-Asp) box polypeptide 42	2,6	-2,4	0,4	0,0
ENSRNOG00000025074	NM_012559	FGG	fibrinogen gamma chain	2,6	-1,9	0,0	2,1
ENSRNOG00000016346	NM_133307	PRKCD	protein kinase C, delta	2,6	-4,3	2,4	3,8
ENSRNOG00000008510	NM_134403	ABTB2	ankyrin repeat and BTB (POZ) domain containing 2	2,5	-2,4	0,5	3,8
ENSRNOG00000026455	XP_343499.2	GPD1L	glycerol-3-phosphate dehydrogenase 1-like	2,5	-2,1	0,2	3,5
ENSRNOG00000024772	XM_221075.5	Cpsf4l	cleavage and polyadenylation specific factor 4-like	2,5	-3,2	1,4	0,0
ENSRNOG00000002878	NM_172320	AFM	afamin	2,5	-3,2	1,3	0,0
ENSRNOG00000005931	NP_113828.1	Pgcp	plasma glutamate carboxypeptidase	2,5	-2,3	0,4	2,6
ENSRNOG00000017765	NM_001039023.1	Net1	neuroepithelial cell transforming 1	2,4	-2,8	1,0	1,4
ENSRNOG00000017489	XP_226533.3	Gse1	genetic suppressor element 1	2,4	-3,1	1,3	0,0
ENSRNOG00000001764	XM_221315.6	vps8	vacuolar protein sorting 8 homolog (S. cerevisiae)	2,4	-3,9	2,2	2,9
ENSRNOG00000002203	NM_001100564.1	Lin54	lin-54 homolog (C. elegans)	2,4	-4,0	2,2	1,0
ENSRNOG00000011111	XP_234430.2	RGD1309492	similar to mKIAA1737 protein	2,3	-0,8	-1,0	3,5
ENSRNOG00000001229	Q9QZD2	COL18A1	collagen, type XVIII, alpha 1	2,3	-0,6	-1,2	4,5
ENSRNOG00000010079	NM_019292	CAR3	carbonic anhydrase 3	2,2	-1,1	-0,6	0,6
ENSRNOG00000004685	ENSRNOG00000004685	Mta3	metastasis associated 1 family, member 3	2,2	-3,8	2,1	0,6
ENSRNOG00000018899	XM_003749455.1	C5	complement component 5	2,2	-0,4	-1,2	3,8
ENSRNOG00000016595	NM_024385	hhex	hematopoietically expressed homeobox	2,2	-2,3	0,6	0,0
ENSRNOG00000004601	XP_230613.2	HAO1	hydroxyacid oxidase (glycolate oxidase) 1	2,2	-3,4	1,8	0,0
ENSRNOG00000001407	XP_222022.3	tfr2	transferrin receptor 2	2,2	0,9	-2,6	3,5
ENSRNOG00000002280	XP_217566.2	SH3BGRL	SH3 domain binding glutamic acid-rich protein like	2,1	-1,9	0,3	5,1
ENSRNOG00000012190	XP_236535.1	CLDN2	claudin 2	2,1	-0,7	-0,9	5,1
ENSRNOG00000004786	NM_019623.2	Cyp4f1	cytochrome P450 4F1	2,1	-2,3	0,7	2,6
ENSRNOG00000012878	NM_012511	Atp7b	ATPase, Cu++ transporting, beta polypeptide	2,1	-3,4	1,8	1,6
ENSRNOG00000020928	NM_012542	Cyp2a1	cytochrome P450 2A1 (hepatic steroid hydroxylase IIA1)	2,1	-1,4	-0,1	5,1
ENSRNOG00000009549	NM_001109606.1	Fbxo3	F-box protein 3	2,1	-0,3	-1,3	5,1

ENSRNOG00000014743	NM_033349	HAGH	hydroxyacyl glutathione hydrolase	2,0	-1,5	0,0	5,1
ENSRNOG00000000033	XP_223107.3	Tmcc2	transmembrane and coiled-coil domain family 2	2,0	-3,4	1,9	1,6
ENSRNOG00000020928	NM_134369	Cyp2a1	cytochrome P450 2A1 (hepatic steroid hydroxylase IIA1)	2,0	-0,8	-0,7	2,9
ENSRNOG00000011026	NP_001012488.1	Pqcp	polyglutamine-containing protein	2,0	-2,0	0,5	4,5
ENSRNOG00000003357	NM_032085.1	Col3a1	collagen, type III, alpha 1	2,0	-1,9	0,4	2,1
ENSRNOG00000025510	NP_942071.1	Q62669	beta-globin	2,0	-0,6	-0,8	1,4
ENSRNOG00000020637	XP_219327.3	HMX3	H6 family homeobox 3	1,9	-3,2	1,8	5,1
ENSRNOG00000001979	NM_153724	RCAN1	regulator of calcineurin 1	1,9	-2,1	0,7	0,0
ENSRNOG00000025056	ENSRNOG00000025056	LOC314328	similar to chromosome 14 open reading frame 166B	1,9	-1,9	0,5	0,0
ENSRNOG00000014565	NP_955437.1	LCMT1	leucine carboxyl methyltransferase 1	1,8	-2,3	0,9	1,4
ENSRNOG00000019467	NM_001106534.2	Fer1l4	fer-1-like 4 (C. elegans)	1,8	-2,2	0,8	2,6
ENSRNOG00000012926	NP_001000754.1	Olr363	olfactory receptor 363	1,8	-1,1	-0,3	2,1
ENSRNOG00000001475	ENSRNOG00000001475	wbscr27	Williams Beuren syndrome chromosome region 27	1,8	-3,1	1,7	3,5
ENSRNOG00000025106	ENSRNOG00000025106	LOC502175	similar to peroxiredoxin 1	1,8	-2,2	0,8	0,0
ENSRNOG00000023116	XP_343055.2	Tmem195	transmembrane protein 195	1,8	-3,1	1,8	0,0
ENSRNOG00000027742	XP_231125.3	ADAMTSL2	ADAMTS-like 2	1,7	0,2	-1,6	3,5
ENSRNOG00000006042	Q91XP0	Rup2	urinary protein 2	1,7	-2,6	1,3	0,0
ENSRNOG00000020676	NM_130403	Ppp1r14a	protein phosphatase 1, regulatory (inhibitor) subunit 14A	1,7	-2,4	1,1	1,6
ENSRNOG00000003456	NM_001013961.1	Actrt1	actin-related protein T1	1,7	2,0	-3,2	2,9
ENSRNOG00000011269	NM_031732	SULT1C3	sulfotransferase family, cytosolic, 1C, member 3	1,7	-1,8	0,5	0,6
ENSRNOG00000024848	NM_052797.1	Fga	fibrinogen alpha chain	1,7	-1,9	0,6	1,7
ENSRNOG00000016791	NM_017127.1	ChkA	choline kinase alpha	1,7	-1,9	0,6	0,6
ENSRNOG00000004332	NM_031507	egfr	epidermal growth factor receptor	1,6	-1,5	0,2	3,5
ENSRNOG00000019441	NM_017265.4	Hsd3b6	hydroxy-delta-5-steroid dehydrogenase, 3 beta- and steroid delta-isomerase 6	1,6	-2,7	1,5	0,6
ENSRNOG00000010478	NM_182474	SPI1	serine protease inhibitor	1,6	-2,5	1,3	0,0
ENSRNOG00000019417	NM_012584	Hsd3b5	hydroxy-delta-5-steroid dehydrogenase, 3 beta- and steroid delta-isomerase 5	1,6	-2,5	1,3	0,0
ENSRNOG00000000419	NP_997631.1	CFB	complement factor B	1,6	0,5	-1,7	4,5
ENSRNOG00000011864	NM_172335	gm2a	GM2 ganglioside activator	1,6	-1,5	0,3	2,9
ENSRNOG00000025286	XP_215826.3	PANK2	pantothenate kinase 2 (Hallervorden-Spatz syndrome)	1,5	-2,6	1,4	3,8
ENSRNOG00000014900	NM_001271248.1	Crem	cAMP responsive element modulator	1,5	-1,6	0,5	2,6
ENSRNOG00000022239	XP_340856.1	TUSC5	tumor suppressor candidate 5	1,5	-2,5	1,4	2,6
ENSRNOG00000001823	NM_001113344.1	St6gal1	ST6 beta-galactosamide alpha-2,6-sialyltranferase 1	1,5	-2,5	1,4	2,1
ENSRNOG00000010309	NM_031553	NFYB	nuclear transcription factor-Y beta	1,4	-2,2	1,1	2,1
ENSRNOG00000003763	NM_053512	Prdx4	peroxiredoxin 4	1,4	-2,5	1,4	3,5
ENSRNOG00000012315	NM_001048185	agmaT	agmatine ureohydrolase (agmatinase)	1,4	-2,0	1,0	4,5
ENSRNOG00000017843	NP_001014281.1	POLR3K	polymerase (RNA) III (DNA directed) polypeptide K, 12.3 kDa	1,4	-2,3	1,3	4,5
ENSRNOG00000016620	NM_022288.1	Csnk1g1	casein kinase 1, gamma 1	1,4	-1,5	0,5	4,5
ENSRNOG00000018299	XP_225875.3	RGD1564677	similar to transcription factor ONECUT2	1,4	-2,0	1,0	4,5
ENSRNOG00000006709	NM_145779	PZP	pregnancy-zone protein	1,3	-2,2	1,2	2,9
ENSRNOG00000008145	XP_343114.2	TRAF3	Tnf receptor-associated factor 3	1,3	1,5	-2,5	5,1
ENSRNOG00000000604	Q99PA1	HDAC2	similar to Histone deacetylase 2 (HD2); histone deacetylase 2	1,3	-3,2	2,3	0,0
ENSRNOG00000014285	NM_001107024.1	Ssh2	slingshot protein phosphatase 2	1,3	-2,0	1,0	4,5
ENSRNOG00000019996	NM_012716	SLC16A1	solute carrier family 16, member 1 (monocarboxylic acid transporter 1)	1,2	-2,2	1,3	1,0
ENSRNOG00000002021	NM_001170328.1	Son	Son DNA binding protein	1,2	1,1	-2,0	5,1
ENSRNOG00000009582	NM_053562	RPE65	retinal pigment epithelium 65	1,2	0,7	-1,6	2,9

ENSRNOG00000017223	NM_053491	plg	plasminogen	1,2	-1,7	0,8	4,5
ENSRNOG00000025019	ENSRNOG00000025019	DIO1	deiodinase, iodothyronine, type I	1,2	-2,3	1,5	2,9
ENSRNOG00000001062	NM_001246665	Setd8	SET domain containing (lysine methyltransferase) 8	1,2	-2,0	1,2	4,5
ENSRNOG00000016123	XP_344577.2	Rnf144b	ring finger protein 144B	1,1	-1,8	1,0	3,5
ENSRNOG00000025079	NP_001020881.1	FAM126B	family with sequence similarity 126, member B	1,1	-1,6	0,7	2,6
ENSRNOG00000019262	XP_236715.3	RGD1307844	similar to 106 kDa O-GlcNAc transferase-interacting protein	1,1	-2,0	1,2	2,1
ENSRNOG00000005109	NM_001044276.1	Rprm	reprimo, TP53 dependent G2 arrest mediator candidate	1,1	-1,5	0,7	5,1
ENSRNOG00000018322	NM_053554	PICALM	phosphatidylinositol binding clathrin assembly protein	1,1	-2,2	1,4	3,8
ENSRNOG00000017923	NM_134468	pncK	pregnancy upregulated non-ubiquitously expressed CaM kinase	1,1	-1,4	0,6	5,1
ENSRNOG00000004329	XM_002726903	Rdh2	Rattus norvegicus Rdh2 protein-like, transcript variant 1 (LOC100365958)	1,1	-1,1	0,3	5,1
ENSRNOG00000009284	NM_012742	foxa1	forkhead box A1	1,1	-2,1	1,3	1,0
ENSRNOG00000019460	NM_017199	SSRD	signal sequence receptor, delta (Ssr4),	1,0	-1,9	1,2	5,1
ENSRNOG00000000028	NM_053347	NDE1	nudE nuclear distribution gene E homolog 1 (A. nidulans)	1,0	1,1	-1,9	3,8
ENSRNOG00000018340	XP_345146.2	MARVELD2	MARVEL domain containing 2	1,0	-2,1	1,3	4,5
ENSRNOG00000014766	NM_001013089.2	Galt	galactose-1-phosphate uridylyltransferase	1,0	-2,0	1,2	1,7
ENSRNOG00000027026	XM_001065610.3	JAZF1	JAZF zinc finger 1	1,0	-2,1	1,4	5,1
ENSRNOG00000009609	ENSRNOG00000009609	GTPBP4	Nucleolar GTP-binding protein 1 (Chronic renal failure gene protein)	1,0	-2,6	1,9	0,6
ENSRNOG00000002001	NM_019227	ITSN1	intersectin 1 (SH3 domain protein)	0,9	-2,2	1,5	4,5
ENSRNOG00000009273	NP_671745.1	MUP	alpha 2U globulin; Major urinary protein precursor (MUP)	0,9	-1,6	0,9	1,7
ENSRNOG00000006388	NM_022268	PYGL	phosphorylase, glycogen, liver	0,8	-2,4	1,8	0,0
ENSRNOG00000000786	NP_001002831.1	rpp21	ribonuclease P 21 subunit (human)	0,8	-1,8	1,1	2,6
ENSRNOG00000002104	NM_001037347.2	Scaf4	splicing factor, arginine/serine-rich 15	0,8	-1,8	1,1	5,1
ENSRNOG00000020608	NP_001011980.1	ppan	peter pan homolog (Drosophila)	0,8	-2,7	2,1	0,6
ENSRNOG00000018764	XP_224719.1	B3gnt3	UDP-GlcNAc:betaGal beta-1,3-N-acetylglucosaminyltransferase 3	0,8	1,0	-1,6	1,6
ENSRNOG00000001064	ENSRNOG00000001064	Sbno1	strawberry notch homolog 1 (Drosophila)	0,8	-2,1	1,5	5,1
ENSRNOG00000013520	NM_012860	mat2a	methionine adenosyltransferase II, alpha	0,8	-2,6	2,0	1,7
ENSRNOG00000000978	NM_153312	CYP3A2	Cytochrome P450 3A2	0,8	0,8	-1,3	4,5
ENSRNOG00000007240	XP_232622.2	rrs1	RRS1 ribosome biogenesis regulator homolog (S. cerevisiae)	0,8	-3,3	2,7	0,0
ENSRNOG00000015772	NM_022539	RGD1560341	similar to Methionine aminopeptidase 2 (MetAP 2)	0,7	-3,0	2,4	1,0
ENSRNOG00000027002	XM_003749177	LOC100909748	PREDICTED: microtubule-associated serine/threonine-protein kinase 4-like	0,7	-2,0	1,4	4,5
ENSRNOG00000020793	NP_001020576.1	RUVBL2	RuvB-like 2 (E. coli); similar to RuvB-like 2; similar to RuvB-like 2 (p47 protein)	0,7	-2,8	2,3	0,6
ENSRNOG00000007177	NP_001020294.2	kdelc2	KDEL (Lys-Asp-Glu-Leu) containing 2	0,7	-1,7	1,2	5,1
ENSRNOG00000017946	NM_139262	Ctsq	cathepsin Q	0,7	-1,4	0,9	5,1
ENSRNOG00000003625	NP_001012025.1	UBXN4	UBX domain protein 4	0,6	-2,0	1,5	5,1
ENSRNOG00000017397	NP_001012159.1	upf3a	UPF3 regulator of nonsense transcripts homolog A (yeast)	0,6	-1,9	1,5	3,8
ENSRNOG00000000064	NM_080481	Atp5i	ATP synthase, H+ transporting, mitochondrial F0 complex, subunit E	0,6	-2,2	1,7	2,1
ENSRNOG00000015989	XP_214491.2	mRpL32	mitochondrial ribosomal protein L32	0,6	-2,4	2,0	2,1
ENSRNOG00000027392	ENSRNOG00000027392	RGD1565512	similar to hypothetical protein 4932418E24	0,6	-1,9	1,5	5,1
ENSRNOG00000010756	NM_030989	tp53	tumor protein p53	0,6	-2,3	1,8	3,8
ENSRNOG00000026638	NM_031726.1	Scamp5	secretory carrier membrane protein 5	0,5	1,4	-1,8	3,5
ENSRNOG00000006217	NM_001108064.1	Wiz	widely-interspaced zinc finger motifs	0,5	-3,1	2,7	1,7
ENSRNOG00000019630	NM_053493	Hacl1	2-hydroxyacyl-CoA lyase 1	0,5	-2,4	2,0	0,0
ENSRNOG00000018168	NP_001009601.1	KLC4	kinesin light chain 4	0,5	-1,8	1,5	1,6
ENSRNOG00000004035	XP_235128.2	Hrb2	HIV-1 Rev binding protein 2	0,5	-2,8	2,4	2,1
ENSRNOG00000001338	NM_017233	hpd	4-hydroxyphenylpyruvate dioxygenase	0,4	1,3	-1,6	4,5

ENSRNOG00000011913	NM_012532	CP	ceruloplasmin (ferroxidase)	0,4	1,2	-1,5	4,5
ENSRNOG00000027761	XP_229199.3	htatsf1	HIV-1 Tat specific factor 1	0,4	-2,2	1,9	4,5
ENSRNOG00000013004	NM_138884	AKR1D1	aldo-keto reductase family 1, member D1 (delta 4-3-ketosteroid-5-beta-reductase)	0,4	-1,5	1,3	2,9
ENSRNOG00000001379	NM_147206	Cyp3a9	cytochrome P450 3A9	0,3	1,3	-1,5	3,8
ENSRNOG00000018704	NM_022869	NOLC1	nucleolar and coiled-body phosphoprotein 1	0,3	-1,6	1,4	3,5
ENSRNOG00000020194	NM_001013179.1	Hes6	hairy and enhancer of split 6 (Drosophila)	0,3	1,0	-1,2	4,5
ENSRNOG00000004006	NM_012699	dnajb9	DnaJ (Hsp40) homolog, subfamily B, member 9	0,3	-1,5	1,2	3,8
ENSRNOG00000024745	XM_003749950	MUP	alpha 2U globulin; Major urinary protein precursor (MUP)	0,3	-2,6	2,4	0,0
ENSRNOG00000011784	NM_001106881.1	Mocs1	molybdenum cofactor synthesis 1	0,3	1,7	-1,9	3,8
ENSRNOG00000001990	NM_173295	Ugt2b1	UDP glucuronosyltransferase 2 family, polypeptide B17	0,2	1,6	-1,8	1,4
ENSRNOG00000011728	NM_001039713.1	Fto	fat mass and obesity associated	0,2	-2,4	2,2	4,5
ENSRNOG00000009329	NM_145775	Nr1d1	nuclear receptor subfamily 1, group D, member 1 (REV-ERBalpha)	0,2	1,3	-1,4	3,8
ENSRNOG00000011414	NM_031595.1	Psmc3	proteasome (prosome, macropain) 26S subunit, ATPase 3	0,1	1,4	-1,5	5,1
ENSRNOG00000005064	NM_031138	ube2b	ubiquitin-conjugating enzyme E2B, RAD6 homology (S. cerevisiae)	0,1	-2,1	2,0	4,5
ENSRNOG00000026880	XM_003751862.1	Usp38	ubiquitin specific peptidase 38	0,1	-1,9	1,8	3,5
ENSRNOG00000015654	NM_017094.1	GHR	growth hormone receptor	0,1	2,3	-2,3	1,0
ENSRNOG00000019656	NP_001012047.1	BTD	biotinidase	0,1	-1,8	1,8	2,1
ENSRNOG00000020928	NM_012692	Cyp2a1	cytochrome P450 2A1 (hepatic steroid hydroxylase IIA1)	-0,2	1,3	-1,2	4,5
ENSRNOG00000027116	XR_146223	LOC100362689	PREDICTED: Rattus norvegicus keratin 8-like	-0,2	2,3	-2,2	0,6
ENSRNOG00000001515	XP_230983.3	RGD1561394	similar to MLTK-beta	-0,2	2,1	-2,0	3,5
ENSRNOG00000027142	XP_578505.1	RGD1565680	similar to Hypothetical protein DJ1198H6.2	-0,2	1,7	-1,5	3,8
ENSRNOG00000000658	NM_053922.1	Acacb	acetyl-Coenzyme A carboxylase beta	-0,3	1,8	-1,6	3,5
ENSRNOG00000011635	NM_001100477.1	Ces2e	carboxylesterase 5	-0,3	1,2	-1,0	3,8
ENSRNOG00000013956	NM_134467	Rnf38	ring finger protein 38	-0,3	1,4	-1,2	5,1
ENSRNOG00000009779	NM_199370.1	krt8	keratin 8	-0,3	1,7	-1,5	2,6
ENSRNOG00000014886	NP_001020205.1	fam107b	family with sequence similarity 107, member B	-0,3	1,6	-1,4	4,5
ENSRNOG00000015741	NM_133611	SLC2A13	solute carrier family 2 (facilitated glucose transporter), member 13	-0,3	-1,8	2,1	4,5
ENSRNOG00000027844	ENSRNOG00000027844	CWC22	similar to CG12750-PA; hypothetical protein LOC500684	-0,3	2,3	-2,0	5,1
ENSRNOG00000005434	NP_001013148.1	SPTBN1	spectrin, beta, non-erythrocytic 1	-0,3	1,9	-1,6	5,1
ENSRNOG00000016841	NM_031039	GPT2	glutamic pyruvate transaminase (alanine aminotransferase) 2	-0,4	1,5	-1,3	5,1
ENSRNOG00000023411	nd	RGD1564982	RGD1564982	-0,4	2,6	-2,4	2,6
ENSRNOG00000008755	NM_145770.2	ACOX1	acyl-CoA oxidase 1, palmitoyl	-0,4	1,8	-1,5	2,1
ENSRNOG00000001240	ENSRNOG00000001240	GSTT3	glutathione S-transferase, theta 3	-0,5	1,2	-0,8	5,1
ENSRNOG00000010663	ENSRNOG00000010663	RGD1307220	similar to RIKEN cDNA E330026B02	-0,5	2,1	-1,7	2,9
ENSRNOG00000020928	NM_012692.1	Cyp2a1	cytochrome P450 2A1 (hepatic steroid hydroxylase IIA1)	-0,6	1,4	-0,9	4,5
ENSRNOG00000013178	XP_214698.3	cmip	c-Maf-inducing protein	-0,6	2,0	-1,5	5,1
ENSRNOG00000020266	Q8K1G4	Eef2,LOC305181	similar to Elongation factor 2 (EF-2); eukaryotic translation elongation factor 2	-0,7	2,1	-1,6	2,9
ENSRNOG00000022047	XM_224924	nd	PREDICTED: Rattus norvegicus 60S ribosomal protein L9-like (RGD1561928)	-0,7	2,2	-1,7	2,9
ENSRNOG00000019319	XP_218965.3	fchsd2	FCH and double SH3 domains 2	-0,7	1,8	-1,3	5,1
ENSRNOG00000019735	NP_001015007.1	DPH2	DPH2 homolog (S. cerevisiae)	-0,7	2,2	-1,6	3,8
ENSRNOG00000018567	NP_112410.1	SLC20A1	solute carrier family 20 (phosphate transporter), member 1	-0,8	1,6	-1,1	3,8
ENSRNOG00000018450	XP_219484.2	SLC25A22	solute carrier family 25 (mitochondrial carrier, glutamate), member 22	-0,8	2,1	-1,5	0,6
ENSRNOG00000016023	XP_219925.3	KANK1	ankyrin repeat domain 15	-0,8	1,7	-1,1	5,1
ENSRNOG00000018566	NM_013156	CTSL1	cathepsin L1	-0,8	1,6	-1,0	4,5
ENSRNOG00000013241	ENSRNOG00000013241	LOC363010	similar to Cytochrome P450 2C7 (CYPIIC7) (P450F) (PTF1)	-0,9	1,3	-0,7	4,5

ENSRNOG00000019383	NM_019194	Tef	thyrotrophic embryonic factor	-0,9	2,0	-1,3	2,6
ENSRNOG00000017601	NM_017070	srd5a1	steroid-5-alpha-reductase, alpha polypeptide 1	-0,9	2,2	-1,5	0,0
ENSRNOG00000009001	NM_001130563.1	Mtss1	metastasis suppressor 1	-0,9	1,8	-1,1	4,5
ENSRNOG00000011708	NM_031572	CYP2c12	Cytochrome P450 2C12, female-specific (CYPIIC12)	-0,9	1,4	-0,7	1,6
ENSRNOG00000022655	XP_573575.1	LOC501166	similar to Hnrpa3 protein	-0,9	1,9	-1,2	5,1
ENSRNOG00000009486	NM_001044770	Cyp4a2	cytochrome P450 4a2	-0,9	1,4	-0,7	3,8
ENSRNOG00000008065	XP_220308.3	WWC1	WW and C2 domain containing 1	-0,9	1,9	-1,2	3,8
ENSRNOG00000017093	ENSRNOG00000017093	RGD1311307	similar to 1300014I06Rik protein	-0,9	2,2	-1,5	2,9
ENSRNOG00000006570	XP_234320.3	PLEKHG3	pleckstrin homology domain containing, family G (with RhoGef domain) member 3	-1,0	2,0	-1,3	4,5
ENSRNOG00000019503	NP_001011916.1	Rec8	REC8 homolog (yeast)	-1,0	2,0	-1,3	2,6
ENSRNOG00000005906	NM_031561	CD36	FAT/CD36 (thrombospondin receptor)	-1,0	2,1	-1,4	0,0
ENSRNOG00000021095	NM_172317	fxyd3	FXYD domain-containing ion transport regulator 3	-1,0	2,5	-1,7	4,5
ENSRNOG00000015701	XP_225250.3	Rreb1	ras responsive element binding protein 1	-1,0	2,2	-1,5	2,1
ENSRNOG00000001205	XP_215367.2	AGPAT3	1-acylglycerol-3-phosphate O-acyltransferase 3	-1,0	1,8	-1,0	3,8
ENSRNOG00000013469	XP_225257.2	TXNDC5	thioredoxin domain containing 5	-1,0	2,8	-2,0	1,7
ENSRNOG00000016818	NM_053429	fgfr3	fibroblast growth factor receptor 3	-1,0	2,9	-2,2	0,6
ENSRNOG00000019832	XP_219691.3	Bbs1	Bardet-Biedl syndrome 1 homolog (human)	-1,1	2,3	-1,6	1,4
ENSRNOG00000019369	NP_001009292.1	Giyd2	GIY-YIG domain containing 2	-1,1	2,4	-1,6	4,5
ENSRNOG00000021102	NM_017288	SCN1B	sodium channel, voltage-gated, type I, beta	-1,1	2,1	-1,3	2,1
ENSRNOG00000011351	NM_012860.2	Mat1a	methionine adenosyltransferase I, alpha	-1,1	1,7	-0,9	2,1
ENSRNOG00000008116	NM_138905	Ppap2b	phosphatidic acid phosphatase type 2B	-1,1	1,7	-0,9	3,5
ENSRNOG00000019308	NM_012911	arrb2	arrestin, beta 2	-1,1	2,2	-1,4	4,5
ENSRNOG00000017188	NM_178847.2	Cyp27a1	cytochrome P450 27A1 (sterol 26-hydroxylase, mitochondrial)	-1,2	1,4	-0,5	3,5
ENSRNOG00000005357	XP_220595.3	RGD1563106	similar to novel protein	-1,2	2,6	-1,7	2,6
ENSRNOG00000004474	XP_216721.2	Klhdc2	kelch domain containing 2	-1,2	2,1	-1,2	4,5
ENSRNOG00000021027	NM_012543	dbp	D site of albumin promoter (albumin D-box) binding protein	-1,2	1,7	-0,8	2,9
ENSRNOG00000002358	NM_133383	Scpep1	serine carboxypeptidase 1	-1,2	2,1	-1,2	3,5
ENSRNOG00000027302	ENSRNOG00000027302	Pcdhgb2	protocadherin gamma subfamily B, 2	-1,2	1,9	-0,9	5,1
ENSRNOG00000019494	NP_001020808.1	PSMB10	proteasome (prosome, macropain) subunit, beta type 10	-1,2	1,9	-1,0	4,5
ENSRNOG00000020029	ENSRNOG00000020029	nd	hypothetical protein LOC682248; similar to RIKEN cDNA 9530058B02	-1,2	2,8	-1,8	0,6
ENSRNOG00000010048	NM_024130	DCTN1	dynactin 1	-1,3	2,1	-1,1	3,8
ENSRNOG00000017749	NM_133540	Nkg7	natural killer cell group 7 sequence	-1,4	1,3	-0,3	3,8
ENSRNOG00000017808	NM_053536	klf15	Kruppel-like factor 15	-1,4	2,7	-1,7	1,0
ENSRNOG00000008167	NP_001007681.1	ABHD6	abhydrolase domain containing 6	-1,4	2,5	-1,5	1,6
ENSRNOG00000028377	ENSRNOG00000028377	Mpl	myeloproliferative leukemia virus oncogene	-1,4	2,9	-1,8	1,0
ENSRNOG00000001442	NM_031576	por	P450 (cytochrome) oxidoreductase	-1,4	1,7	-0,7	1,0
ENSRNOG00000021463	NM_013196	PPARA,	peroxisome proliferator activated receptor alpha	-1,4	2,6	-1,5	1,0
ENSRNOG00000012622	XP_238034.3	mmp15	matrix metallopeptidase 15	-1,4	2,6	-1,5	0,6
ENSRNOG00000015002	XP_220745.2	ABHD15	abhydrolase domain containing 15	-1,4	2,2	-1,1	3,8
ENSRNOG00000028504	XP_575987.1	SOCS5	suppressor of cytokine signaling 5	-1,5	1,7	-0,6	5,1
ENSRNOG00000017882	NM_001107365	Camk1d	calcium/calmodulin-dependent protein kinase ID	-1,5	2,1	-1,0	2,9
ENSRNOG00000023238	XP_347259.2	RGD1563309	similar to diacylglycerol kinase, delta 130kDa isoform 1	-1,5	1,8	-0,7	5,1
ENSRNOG00000016541	NP_001003401.1	Enc1	ectodermal-neural cortex 1	-1,5	1,4	-0,3	4,5
ENSRNOG00000019956	NM_001107966.1	Kdm4A	lysine (K)-specific demethylase 4A	-1,5	1,6	-0,5	3,5
ENSRNOG00000010635	NM_001004274.2	igfbp4	insulin-like growth factor binding protein 4	-1,5	3,1	-1,9	0,0

ENSRNOG00000015082	NM_001106291.1	Nlrp10	NLR family, pyrin domain containing 10	-1,5	2,4	-1,2	5,1
ENSRNOG00000010224	NM_001015012.1	Rab30	RAB30, member RAS oncogene family	-1,5	1,5	-0,3	1,6
ENSRNOG00000011201	NP_942049.1	CMTM8	CKLF-like MARVEL transmembrane domain containing 8	-1,5	5,3	-4,1	1,0
ENSRNOG00000022067	NM_001145828.1	Tlr5	toll-like receptor 5	-1,6	1,8	-0,7	4,5
ENSRNOG00000023718	XP_218099.3	Vom2r2	similar to vomeronasal 2, receptor, 1; similar to vomeronasal 2, receptor, 2	-1,6	2,2	-1,0	5,1
ENSRNOG00000020930	XP_213493.3	RGD1311493	similar to CG13379-PA	-1,6	2,8	-1,6	2,9
ENSRNOG00000019481	NM_031241	Cyp8b1	cytochrome P450 8B1	-1,6	2,8	-1,6	0,0
ENSRNOG00000021380	XP_221105.3	FADS6	fatty acid desaturase domain family, member 6	-1,6	1,4	-0,2	2,1
ENSRNOG00000008190	NM_144738	PNPLA7	patatin-like phospholipase domain containing 7	-1,6	2,1	-0,9	1,6
ENSRNOG00000001404	ENSRNOG00000001404	AGFG2	ArfGAP with FG repeats 2	-1,6	2,0	-0,8	5,1
ENSRNOG00000023465	ENSRNOG00000023465	LOC500300	similar to hypothetical protein MGC6835	-1,6	1,4	-0,1	2,1
ENSRNOG00000010216	ENSRNOG00000010216	RGD1308772	similar to KIAA0564 protein	-1,6	3,0	-1,8	1,6
ENSRNOG00000004262	NM_022799	Nucks1	nuclear casein kinase and cyclin-dependent kinase substrate 1	-1,6	2,2	-1,0	2,6
ENSRNOG00000001312	NM_012801	PDGA	Platelet-derived growth factor subunit A	-1,7	2,4	-1,2	2,1
ENSRNOG00000018145	NM_001004085.2	Crat	carnitine acetyltransferase	-1,7	1,5	-0,3	2,1
ENSRNOG00000001259	ENSRNOG00000001259	Cux2	similar to Homeobox protein cut-like 2 (Homeobox protein Cux-2) (Cut-like 2)	-1,7	3,1	-1,8	0,6
ENSRNOG00000015409	XP_238380.2	USP5	ubiquitin specific peptidase 5 (isopeptidase T)	-1,7	3,3	-2,0	2,1
ENSRNOG00000014168	NP_001028876.1	xylB	xylulokinase homolog (H. influenzae)	-1,8	1,7	-0,4	3,5
ENSRNOG00000019691	NM_053425	Ccs	copper chaperone for superoxide dismutase	-1,8	2,8	-1,5	2,1
ENSRNOG00000020308	NM_022594	ech1	enoyl coenzyme A hydratase 1, peroxisomal	-1,8	1,7	-0,4	1,6
ENSRNOG00000007104	NM_001270597.1	Itpr1	inositol 1,4,5-triphosphate receptor, type 1	-1,8	3,3	-2,0	1,7
ENSRNOG00000020238	XP_340910.2	Plekhh3	pleckstrin homology domain containing, family H (with MyTH4 domain) member 3	-1,8	1,9	-0,5	1,7
ENSRNOG00000026073	NP_001008948.1	V1ra8	vomeronasal 1 receptor, A8	-1,8	2,6	-1,3	3,8
ENSRNOG00000005341	NM_001106481.1	Upp2	uridine phosphorylase 2	-1,9	3,2	-1,8	0,0
ENSRNOG00000008498	NM_001044294.1	Gabarapl1	GABA(A) receptor-associated protein like 1	-1,9	2,2	-0,7	2,6
ENSRNOG00000017613	XP_225072.3	Pols	polymerase (DNA directed) sigma	-2,0	2,1	-0,6	2,9
ENSRNOG00000016678	NM_133569	ANGPTL2	angiopoietin-like 2	-2,0	2,7	-1,2	2,9
ENSRNOG00000001197	NM_031769	pdxK	pyridoxal (pyridoxine, vitamin B6) kinase	-2,0	2,6	-1,1	1,6
ENSRNOG00000016097	NM_001013164.3	Ccbl1	cysteine conjugate-beta lyase, cytoplasmic	-2,0	2,5	-1,0	1,0
ENSRNOG00000004294	NM_022384	Ascl1	achaete-scute complex homolog 1 (Drosophila)	-2,1	3,7	-2,1	0,0
ENSRNOG00000013728	XP_221047.2	POLG2	polymerase (DNA directed), gamma 2, accessory subunit	-2,2	1,7	-0,1	4,5
ENSRNOG00000008046	XP_234295.1	TMEM30B	transmembrane protein 30B	-2,2	2,0	-0,4	2,1
ENSRNOG00000002013	NM_001100653.1	Sidt1	SID1 transmembrane family, member 1	-2,2	2,6	-0,9	2,6
ENSRNOG00000009005	NM_022667.2	Slco2a1	solute carrier organic anion transporter family, member 2a1	-2,2	2,4	-0,7	1,0
ENSRNOG00000016573	NP_001012345.1	DGAT2	diacylglycerol O-acyltransferase homolog 2 (mouse)	-2,2	2,9	-1,2	0,0
ENSRNOG00000018736	XP_341961.1	PNPLA2	patatin-like phospholipase domain containing 2	-2,3	2,0	-0,3	1,6
ENSRNOG00000016551	NM_171988	BCL2L11	BCL2-like 11 (apoptosis facilitator)	-2,4	2,9	-1,1	1,0
ENSRNOG00000011696	NM_031048	Lifr	leukemia inhibitory factor receptor alpha	-2,4	3,0	-1,2	0,0
ENSRNOG00000012464	NM_134329	adh1	alcohol dehydrogenase 1 (class I)	-2,4	1,9	-0,1	2,6
ENSRNOG00000005479	NM_017215	Slc1a2	solute carrier family 1 (glial high affinity glutamate transporter), member 2	-2,4	3,0	-1,2	0,0
ENSRNOG00000027024	ENSRNOG00000027024	RGS16	regulator of G-protein signaling 16	-2,6	4,6	-2,7	0,0
ENSRNOG00000012134	NM_013178	SCN4A	sodium channel, voltage-gated, type 4, alpha subunit	-2,7	0,4	1,7	4,5
ENSRNOG00000004821	XP_235312.3	SNTB1	syntrophin, basic 1	-3,1	2,8	-0,5	0,0
ENSRNOG00000003872	NM_133315	SLC40A1	solute carrier family 39 (iron-regulated transporter), member 1	-3,1	4,0	-1,7	0,0
ENSRNOG00000017727	NM_012630	PRLR	prolactin receptor	-3,3	4,0	-1,5	0,0

**ENSRNOG00000013687**	**XM_003749149**	**ABLIM1**	**actin-binding LIM protein 1**	**-3,6**	**3,0**	**-0,3**	**2,1**
**ENSRNOG00000011526**	**NM_012999**	**Pcsk6**	**proprotein convertase subtilisin/kexin type 6**	**-3,7**	**4,3**	**-1,5**	**0,0**
**ENSRNOG00000004692**	**NM_022258**	**A1bg**	**alpha-1-B glycoprotein**	**-4,0**	**7,0**	**-4,0**	**0,0**
**ENSRNOG00000017593**	**NP_110491.1**	**mtr**	**5-methyltetrahydrofolate-homocysteine methyltransferase**	**-4,4**	**4,0**	**-0,7**	**0,0**
**ENSRNOG00000019859**	**NP_001004277.1**	**PLA2G15**	**phospholipase A2, group XV**	**-5,2**	**9,7**	**-5,8**	**2,6**
**ENSRNOG00000017672**	**NM_138547**	**Akr1c14**	**aldo-keto reductase family 1, member C14**	**-6,2**	**4,9**	**-0,3**	**0,0**
**ENSRNOG00000001763**	**XM_221315.5**	**Vps8**	**vacuolar protein sorting 8 homolog (S. cerevisiae)**	**-10,8**	**4,6**	**3,5**	**0,6**

Table S5. Hepatic genes regulated by intermittent GH administration whose mean expression values are different from those in E2 - and E2 plus GH-treated hypothyroid-orchidectomized rats. Hormonal replacements with GH (TXOXGH), E2 (TXOXE2) or E2 plus GH (TXOXE2GH) in TXOX rats were described in Material and Methods. Then, differently expressed genes in the livers were identified by DNA microarrays. SAM multiclass analysis was performed to identify GH regulated genes (TXOXGH) whose mean expression values were significantly different from E2 (TXOXE2)- or E2 plus GH (TXOXE2GH)-treated TXOX rats. The table shows ENSEMBL ID, Unigene/Refseq, gene symbol, gene description, R (mean expression), and q(%).
